# Multi-domain probiotic consortium as an alternative to chemical remediation of oil spills at coral reefs and adjacent sites

**DOI:** 10.1186/s40168-021-01041-w

**Published:** 2021-05-21

**Authors:** Denise P. Silva, Helena D. M. Villela, Henrique F. Santos, Gustavo A. S. Duarte, José Roberto Ribeiro, Angela M. Ghizelini, Caren L. S. Vilela, Phillipe M. Rosado, Carolline S. Fazolato, Erika P. Santoro, Flavia L. Carmo, Dalton S. Ximenes, Adriana U. Soriano, Caio T. C. C. Rachid, Rebecca L. Vega Thurber, Raquel S. Peixoto

**Affiliations:** 1 LEMM, Laboratory of Molecular Microbial Ecology, Institute of Microbiology Paulo de Góes, Federal University of Rio de Janeiro (UFRJ), Rio de Janeiro, Brazil; 2 Department of Marine Biology, Fluminense Federal University (UFF), Niterói, Brazil; 3 Processes Laboratory, Leopoldo Américo Miguez de Mello Research Center (CENPES), Petrobras, Rio de Janeiro, Brazil; 4 Environmental Treatments, Wastes and Water Resources, Leopoldo Américo Miguez de Mello Research Center (CENPES), Petrobras, Rio de Janeiro, Brazil; 5 LABEM, Paulo de Góes Institute of Microbiology, Federal University of Rio de Janeiro (UFRJ), Rio de Janeiro, Brazil; 6 Department of Microbiology, Oregon State University, Nash Hall 226, OSU, Corvallis, OR 97331 USA; 7 Division of Biological and Environmental Science and Engineering (BESE), Red Sea Research Center, King Abdullah University of Science and Technology (KAUST), Thuwal, 23955-6900 Kingdom of Saudi Arabia

**Keywords:** Oil bioremediation, Probiotics, Coral-associated microbiome, Coral reefs, Restoration, Chemical dispersant, Corexit 9500, *Millepora alcicornis*

## Abstract

**Background:**

Beginning in the last century, coral reefs have suffered the consequences of anthropogenic activities, including oil contamination. Chemical remediation methods, such as dispersants, can cause substantial harm to corals and reduce their resilience to stressors. To evaluate the impacts of oil contamination and find potential alternative solutions to chemical dispersants, we conducted a mesocosm experiment with the fire coral *Millepora alcicornis*, which is sensitive to environmental changes. We exposed *M*. *alcicornis* to a realistic oil-spill scenario in which we applied an innovative multi-domain bioremediator consortium (bacteria, filamentous fungi, and yeast) and a chemical dispersant (Corexit® 9500, one of the most widely used dispersants), to assess the effects on host health and host-associated microbial communities.

**Results:**

The selected multi-domain microbial consortium helped to mitigate the impacts of the oil, substantially degrading the polycyclic aromatic and n-alkane fractions and maintaining the physiological integrity of the corals. Exposure to Corexit 9500 negatively impacted the host physiology and altered the coral-associated microbial community. After exposure, the abundances of certain bacterial genera such as *Rugeria* and *Roseovarius* increased, as previously reported in stressed or diseased corals. We also identified several bioindicators of Corexit 9500 in the microbiome. The impact of Corexit 9500 on the coral health and microbial community was far greater than oil alone, killing corals after only 4 days of exposure in the flow-through system. In the treatments with Corexit 9500, the action of the bioremediator consortium could not be observed directly because of the extreme toxicity of the dispersant to *M*. *alcicornis* and its associated microbiome.

**Conclusions:**

Our results emphasize the importance of investigating the host-associated microbiome in order to detect and mitigate the effects of oil contamination on corals and the potential role of microbial mitigation and bioindicators as conservation tools. Chemical dispersants were far more damaging to corals and their associated microbiome than oil, and should not be used close to coral reefs. This study can aid in decision-making to minimize the negative effects of oil and dispersants on coral reefs.

Video abstract

**Supplementary Information:**

The online version contains supplementary material available at 10.1186/s40168-021-01041-w.

## Introduction

Coral reefs are especially sensitive to environmental changes [1], which is becoming apparent as reefs experience increasing mass-bleaching events worldwide [2]. Corals “bleach” when they expel the microalgae living in their cells, without which the host cannot maintain a minimal energy input and will die if conditions are not stabilized [3, 4]. Although climate change is presumed to be the main reason for coral bleaching and the disappearance of modern reefs [2, 5, 6], other factors such as poor water quality and pollution [7–11] can also cause bleaching and damage to coral cells.

Oil spills occur worldwide in marine environments [12–15]. Exposure to chronic oil contamination can impair biological functions in corals, including reproduction and recruitment [16]. Chemical dispersants consist of a mixture of emulsifiers and solvents able to break oil into smaller droplets [17–19]. Previous studies have reported substantial declines in the health of corals in response to short-term exposure (0–96 h) to dispersants, and more severe impacts in response to oil-dispersant mixtures [20]. Among chemical dispersants, Corexit® products are the most commonly used worldwide, applied in some of the largest oil spills and cleanup operations. Oil and dispersants may also disturb the symbioses between corals and a diverse range of associated microorganisms (i.e., viruses, dinoflagellates, archaea, bacteria, and fungi) that are essential for host homeostasis [21–23]. Except for microalgae, symbiotic interactions between corals and other microbial-associated groups are only beginning to be revealed, but studies suggest that they play roles in nutrient cycling [24, 25,26], antibiotic production [27], UV-damage protection [28], the production of photosynthate in the skeleton [29], and coral tissues [30].

Marine host-associated microbes are therefore key drivers of the structuring and functioning of ecosystems [31], and as either single strains or microbial consortia show potential applications as probiotics in conservation endeavors. Neutralization of toxic compounds is an important probiotic trait, with several potential applications for corals, as some coral-associated microbes can serve as oil-bioremediation agents. For example, Santos et al. [32] manipulated bacterial strains to protect corals against oil impacts by developing an oil-degrading bacteria consortium isolated from the coral *Mussismilia harttii* [32]. The authors assessed hydrocarbon degradation through the culture medium, with crude oil as the sole carbon source. Based on the success of this bioremediation study, a strategy for the manipulation of coral microbes was later proposed [21] and validated [33], which used “beneficial microorganisms for corals” (BMCs) to increase overall coral fitness through specific mechanisms. This new research field of coral probiotics opened several possibilities for mitigating threatening impacts on corals, including impacts from oil industry activities. Although past BMC experiments have used only bacteria to defend against pathogen and temperature stress [33], previous research has shown that specific hydrocarbon fractions can be more effectively degraded using a multi-domain coculture of bacteria and fungi [34]. Therefore, our main objectives were to (1) develop an environmentally friendly oil-mitigation alternative to chemical dispersants, i.e., bioremediation, through a multi-domain consortium (putative BMC-bioremediator consortium or pBMC-BC) composed of filamentous fungi, yeasts, and bacteria; (2) evaluate the effects of oil, dispersants, and pBMC-BC on corals; (3) investigate host-associated bacteria to evaluate their response to treatments, and identify microbial indicators for each treatment, thereby increasing our knowledge of potential targets for further surveys.

## Methods

### Selection of an oil-degrading multi-domain microbial consortium

To isolate oil-degrading microorganisms that are representative of coral reefs at Armação dos Búzios, Rio de Janeiro, Brazil, seawater and coral nubbins from *M*. *alcicornis* and *Siderastrea stellata* were collected at Ossos Beach in that municipality (22° 44′ 45″ S, 41° 52′ 54″ W) in September 2014 (3 nubbins of each species of coral) and January 2015 (3 nubbins of *M*. *alcicornis*). The coral fragments were placed in seawater from the sampling site and kept at 4 °C until processed in the laboratory 4 h later. From each species, 5-g fragments were macerated in 45 mL of sterile saline solution (0.85% NaCl) and then shaken with glass beads overnight at 24 °C with constant agitation at 180 rpm. Bacteria were isolated from 100-μL subsamples of dilutions from 10^–6^ to 10^–3^ and from seawater samples of dilutions of 10^–9^ to 10^–1^. We inoculated 4 different Bushnell-Haas (BH) agar media (Sigma-Aldrich, St. Louis, Missouri, USA) in triplicate, and then supplemented the media with water-soluble and insoluble oil fractions (oWSF and oWIF, respectively), each at two salinities, 2.5% and 8% NaCl (details of media preparation in Supplementary Methods). For fungi isolation, 400 mL of water was filtered in a 0.2-μm membrane and poured over a filter pad soaked with BH oWSF and oWIF broth (supplemented with 0.1% glucose and 0.05% yeast extract) in Petri dishes. Aliquots (0.2 mL) of coral macerate were spread on a Petri dishes containing the 4 different BH agar media with 2% malt extract, in triplicate. Petri dishes were incubated at 25 °C and observed after 3, 5, 7, 14, 21, and 28 days. A different morphotype on agar or the filter was transferred to a new dish of BH until single colonies developed. Fungal isolates were then grown in BH liquid media containing oil as the only carbon source, to test the oil-degrading capacity of the strains. Colonies able to grow on the selective media were considered oil-degrading fungi and were classified by colony morphology, color, and visual appearance after 5 days of growth. One representative of each morphotype was selected for identification using DNA sequencing data. The isolates were stored in glycerol (80% v/v) at − 80 °C.

Selected strains (based on different morphology) were submitted to genomic DNA extraction (Wizard Genomic DNA Purification kit; Promega, San Luis Obispo, California, USA) followed by PCR amplification with the primers 27f and 1492r (bacteria) [35]; internal transcribed spacer (ITS) 1 and ITS4 [36] (filamentous fungi); and ITS1 and NL4 [37] (yeast). Purified PCR products were quantified using a Qubit 2.0 Fluorometer dsDNA-type fluorometer (Invitrogen, Carlsbad, California, USA). Amplicons were sequenced (Macrogen, Seoul, South Korea) with the primers 27f, 1492r, 532f, and 907r for bacteria; ITS1 and ITS4 for filamentous fungi; and ITS1, ITS4, NL1, and NL4 for yeast strains. Sequences were processed with the Ribosomal Database Project II and further assembled with the program Bioedit 7.0.5.355 (details in Supplementary Methods). Sequence similarities were annotated using BLASTn and compared with the NCBI database. All sequences were deposited in GenBank under the accession numbers shown in Table S1.

To evaluate the bacterial growth, we inoculated 1% of the 5-mL culture into flasks containing 100 mL of marine broth (MB) medium (Marine Broth 2216, Himedia Laboratories) in triplicate. The flasks were placed in an incubator under constant agitation (150×*g*) at 26 °C and aliquots of 1 mL were taken every 6 h for 48 h. From each aliquot, two measurements were taken: (1) optical density (OD) estimation at 600-nm wavelength, and (2) colony-forming units (CFU) counted from serial dilutions (100 μL was plated in each Petri dish and normalized to a volume of 1 mL). The OD-to-CFU correlation of each individual strain was used to calculate the number of cells, based on the OD values. Bacteria showing low growth rates were not considered for consortium assembly.

**Fig. 1:**
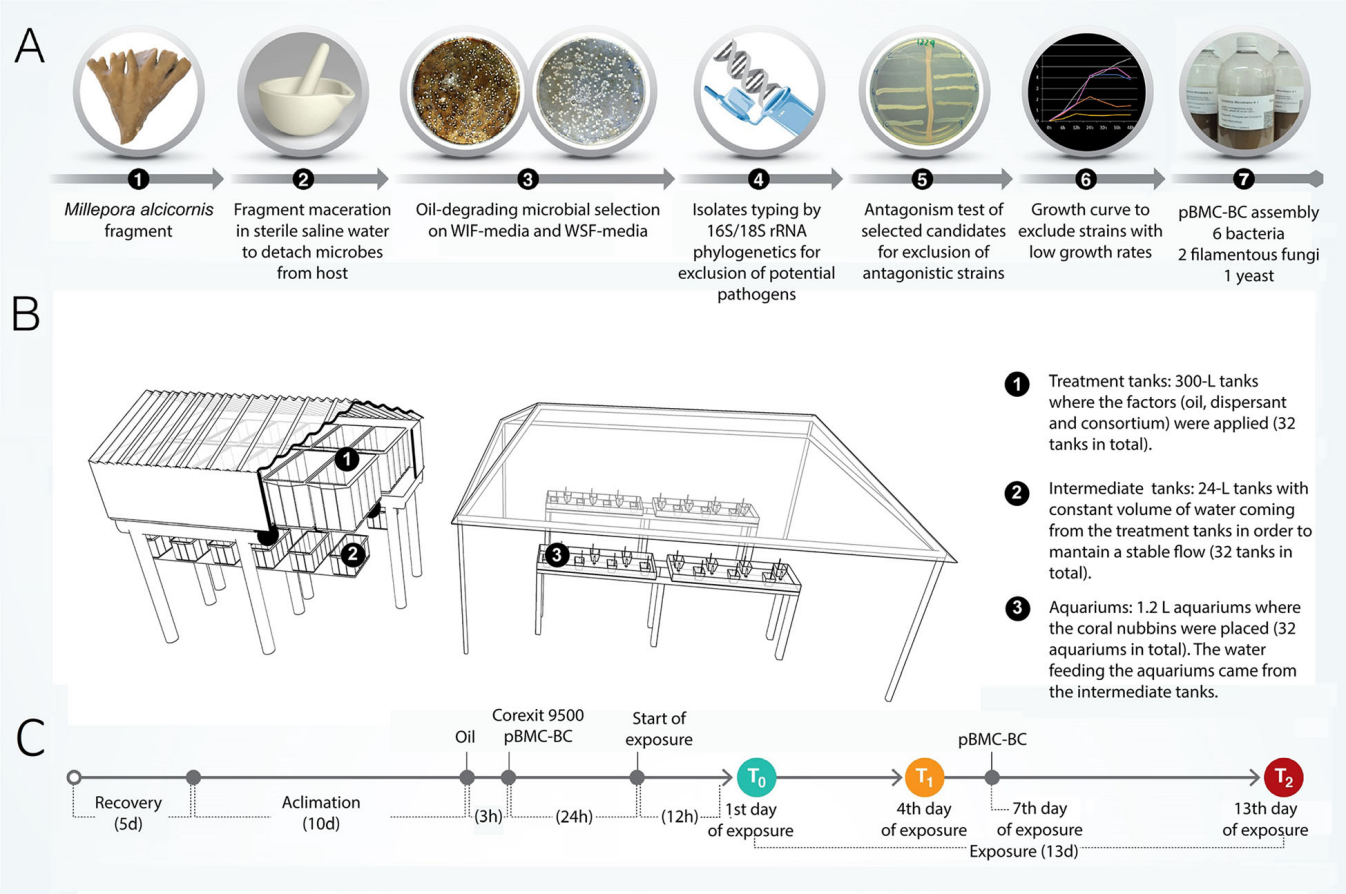
**a** Steps for pBMC-BC selection and timeline of the experiment. **b** Procorais mesocosm structure [38]. **c** Timeline of the experiment showing the collection sample times (*T*_0_, *T*_1_, and *T*_2_)

Paired strains were inoculated cross-wise in the middle of dishes containing Marine Agar (MA) medium, to test for antagonism. The dishes were kept at 28 °C and monitored daily for up to 7 days for antagonistic activity (Fig. 1a).

### Preparation of oil-degrading consortium

Oil-degrading strains from different taxonomic groups that showed no antagonism against each other and that were identified (based on the literature) as neutral or beneficial for corals were selected to compose the pBMC-BC consortium. To prepare the oil-degrading consortium, selected strains were grown individually in 3 L of MB medium for bacteria; 2% malt extract, 2% agar with 2.5% NaCl (MS) for the filamentous fungi; and MS broth for yeast, in sterile 5-L bubble-column bioreactors with a flow rate of 14 L 10^–1^ h. Liquid media with bacteria, yeast, or fungi were centrifuged, and the cell pellet (or fungus propagule) was washed 3 times in sterile saline solution. The pellets from each individual culture were resuspended in saline solution and the OD was measured to estimate the number of bacterial cells. For fungi, the number of propagules was determined by dilution and counting in a Neubauer chamber. Calculations were performed for each culture to reach a final concentration of 10^7^ cells mL^–1^ for bacteria and 10^4^ cells mL^–1^ for fungi. Finally, an equal volume of each isolated culture was mixed in sterile 1-L flasks and aliquoted in 50-mL sterile tubes that were kept at 4 °C until inoculation. The consortium was always prepared 2 days before inoculation, to guarantee the viability of cells.

### Experimental design

The reef-building hydrocoral *M*. *alcicornis* was selected for experimentation. Four individual colonies were collected from different sampling sites at João Fernandes Beach, Armação dos Búzios [38] (details in Supplementary Methods). The four colonies of *M*. *alcicornis* were fragmented into 32 nubbins, each approximately 5 cm in length, totaling 128 nubbins. Four nubbins (replicates per treatment) were glued onto tiles and placed in each of 32 aquariums, where each replicate tank received a nubbin from each one of the four different coral colonies. Therefore, each replicate per treatment is represented by a different coral colony. Treatment blocks and aquariums were then randomly distributed.

For this experiment, we used Marlim crude oil from an oil platform (p-47) located in the Campos Basin, approximately 140 km off Armação dos Búzios. For the dispersant treatment, we used Corexit 9500, one of the most commonly used dispersants worldwide due to its effectiveness in solubilizing oil. The experiment was conducted at the Center for the Study of Oil Bioremediation in Marine Environments, Armação dos Búzios (22° 45′ 44.22′′ S; 41° 53′ 3.97′′ W) in the Procorais mesocosm system. The mesocosm was designed to realistically simulate marine environments and to assess the effects of accidental oil spills as well as remediation, as described by Silva et al. [38]. The treatments were applied in 32 300-L tanks, each containing 250 L of seawater with an air-bubble homogenization system. Then, through gravity flow, the treatments were distributed to 32 24-L polypropylene intermediate tanks and then to each of the 32 aquaria (1.2-L capacity) with a constant flow. The Procorais mesocosm system is adapted to accommodate eight treatments with four true replicates each, for a total of 32 completely independent 2500-L treatment tanks (Fig. 1b).

The experimental treatments were (1) control (seawater only); (2) oWSF (crude oil 1% v/v); (3) pBMC-BC; (4) oWSF + pBMC-BC; (5) Corexit 9500; (6) oWSF + Corexit 9500; (7) pBMC-BC + Corexit 9500; and (8) oWSF + pBMC-BC + Corexit 9500. The dispersant Corexit 9500 was applied at a concentration of 0.05% v/v, following the Brazilian standard [39]. pBMC-BC was applied twice during the experiment (on days 1 and 7 of exposure). To inoculate the consortium, freshly grown bacteria were washed to remove the traces of culture medium and resuspended in sterile saline solution (2.5% NaCl) to a concentration of 10^7^ cells mL^–1^ (Fig. 1a). We then added 250 mL of the 10^7^ cells mL^–1^ solution to each 250-L treatment tank (Fig. 1b). No new water was added to the system during the exposure period; seawater flowed from the pBMC-BC treatment tanks to the intermediate tanks, reaching the aquariums at a final concentration of 10^6^ cells mL^–1^, based on Santos et al. [32]. A timeline of the experiment is detailed in Fig. 1c.

The experiment was conducted for a total of 28 days, including 5 days for *M*. *alcicornis* recovery after fragmentation with high seawater flow (4× the aquarium volume), and 10 days for acclimatization under experimental conditions (0.5× the aquarium volume). These days were defined based on the physical-chemical parameters of the seawater in the aquarium and on an evaluation of the physiology of nubbins through the photosynthetic quantum yield (*F*_*v*_/*F*_*m*_) of the associated photosynthetic algae, as described by Silva et al. [38].

Following the standard procedure for a rapid oil-spill response, we applied the two remediation approaches (Corexit 9500 and pBMC-BC) 3 h after the spill initiation, to simulate a real response team arriving at the affected site. The remediation products acted for 24 h on the oil in a closed system, and after this time, we reopened the system, simulating the time needed for the treatments to reach a nearby reef. The exposure to the treatments lasted 13 days, and pBMC-BC was reinoculated on day 7 of exposure. All samples, corals and water, were collected on days 1 (*T*_0_, the first day after acclimatization), 4 (*T*_1_), and 13 (*T*_2_) of exposure (Fig. 1c). In each sampling time (*T*_0_, *T*_1_, and *T*_2)_, we sampled a nubbin from one of the four different colonies from each aquarium.

### Physico-chemical parameters

Salinity, pH, temperature, dissolved organic carbon (DOC), nitrogen compounds (nitrite, nitrate, and ammonium), and phosphate were sampled, stored, and quantified as described by Silva et al. [38]. Polycyclic aromatic hydrocarbon (PAH) was collected from 1 L of seawater from each replicate, in sterile amber glass bottles with Teflon™ caps. Water samples were stored at 4 °C, and after 24 h the hydrocarbons were extracted and analyzed according to US Environmental Protection Agency (EPA) method 3510C [40]. PAH was detected by gas chromatography/mass spectrometry, following EPA method 8270D [41].

The maximum photosynthetic quantum yield (*F*_*v*_/*F*_*m*_) of the associated photosynthetic algae was measured and related to changes in gross morphology, to estimate impacts on the health of the coral metaorganism. Samples were collected on days 1 (*T*_0_), 4 (*T*_1_), 7, 9, and 13 (*T*_2_) of exposure, using a sub-aquatic pulse and amplitude-modulated fluorometer (Diving-PAM; Heinz Walz, Effeltrich, Germany) .

For each physico-chemical parameter, including *F*_*v*_*/F*_*m*_, we developed mixed-effects models (nlme package [42]; R statistical software [43]) with time and treatment as interactive fixed effects and aquarium identity as a random effect. We calculated least-square means to identify statistically significant (*p* < 0.05) pairwise interactions between specific times and treatments (emmeans package [44] with post-hoc Tukey tests).

### Bacterial community associated with *Millepora alcicornis*

Methods for 16S rRNA gene extraction, amplicon sequencing, and bioinformatics are detailed in Supplementary Methods. Briefly, the total DNA was extracted from 0.5 g of macerated coral nubbins from each aquarium. To sequence the V4 variable region of the 16S rRNA gene, single-step PCR amplification was performed with the primers 515F and 806R  [45]. The multiplexed libraries were sequenced on the Illumina MiSeq platform (Illumina, San Diego, California, USA).

The raw data were analyzed using Mothur version 1.39.5. The paired-end sequences were aligned, pre-clustered, and normalized. To improve the quality of the sequences, the chimeras were removed. Sequences were taxonomically classified with the Ribosomal Database Project 16S rRNA version. The sequences were grouped into Operational Taxonomic Units (OTUs) with a dissimilarity threshold of 3%.

Measurements of α-diversity, i.e., the Shannon and Chao indices, were calculated using Mothur version 1.39.5 and analyzed in PAST, using the ANOVA test. Three-way PERMANOVAs with the Bray-Curtis dissimilarity matrix were performed to evaluate the differences in the structural composition (β-diversity) between treatments, using oil, Corexit, and pBMC-BC as factors, with PRIMER-e version 7.

We created a Bray-Curtis similarity matrix of microbial OTU data, which we subjected to non-parametric multidimensional scaling (NMDS) with normalized OTU abundances to reduce the dimensionality of the OTU data to two axes of primary information [46]. We projected the abiotic parameters onto these axes to identify relationships to microbial composition. This statistical analysis was performed in the Paleontological Statistics Software (PAST) version 3.20.

To evaluate the impacts of oWSF, Corexit, and pBMC-BC on the relative abundance of the OTUs, we performed a blocked indicator species analysis [47] with the PC-ORD software version 6.0. This analysis determines an indicator value for each OTU from 0 (not indicator) to 100 (strong indicator). A value of 100 occurs when a OTU is present in high abundance in all samples of one treatment and at the same time is absent from all samples of the other treatment. A value equal to zero occurs when the distribution is equal regardless of the treatment. For this study, all OTUs with an indicator value above 60 and *p* < 0.05 (Monte Carlo test).

## Results

### Selection of coral-associated microbial assemblage

A total of 57 bacterial isolates were obtained from culture media containing oil hydrocarbons. Isolates identified based on partial 16S rRNA gene sequencing as the genus *Vibrio* or other possible opportunists for any organism were excluded, due to their potential associations with disease and bleaching. The selected bacterial strains showed no antagonistic activity toward each other. The members of the bacterial consortium were identified as *Halomonas aquamarina*, *Pseudoalteromonas shioyasakiensis*, two strains of *Cobetia marina*, *Shewanella* sp., and *Ochrobactrum anthropi*.

A total of 161 fungal (filamentous and yeast) strains were isolated. Of these, 37 showed growth within 5 days of incubation in both fractions (oWSF and oWIF) and were clustered into 9 distinct morphotypes, which were taxonomically identified and predicted to be of low risk to corals or other organisms, based on literature reports. The fungal strains selected for the pBMC-BC assemblage were *Geotrichum* sp., *Rhodotorula mucilaginosa*, and *Penicillium citrinum* (Table S1).

### Exposure to different treatments alters coral physiology and pBMC-BC protects *M*. *alcicornis* against oil impacts

**Fig. 2:**
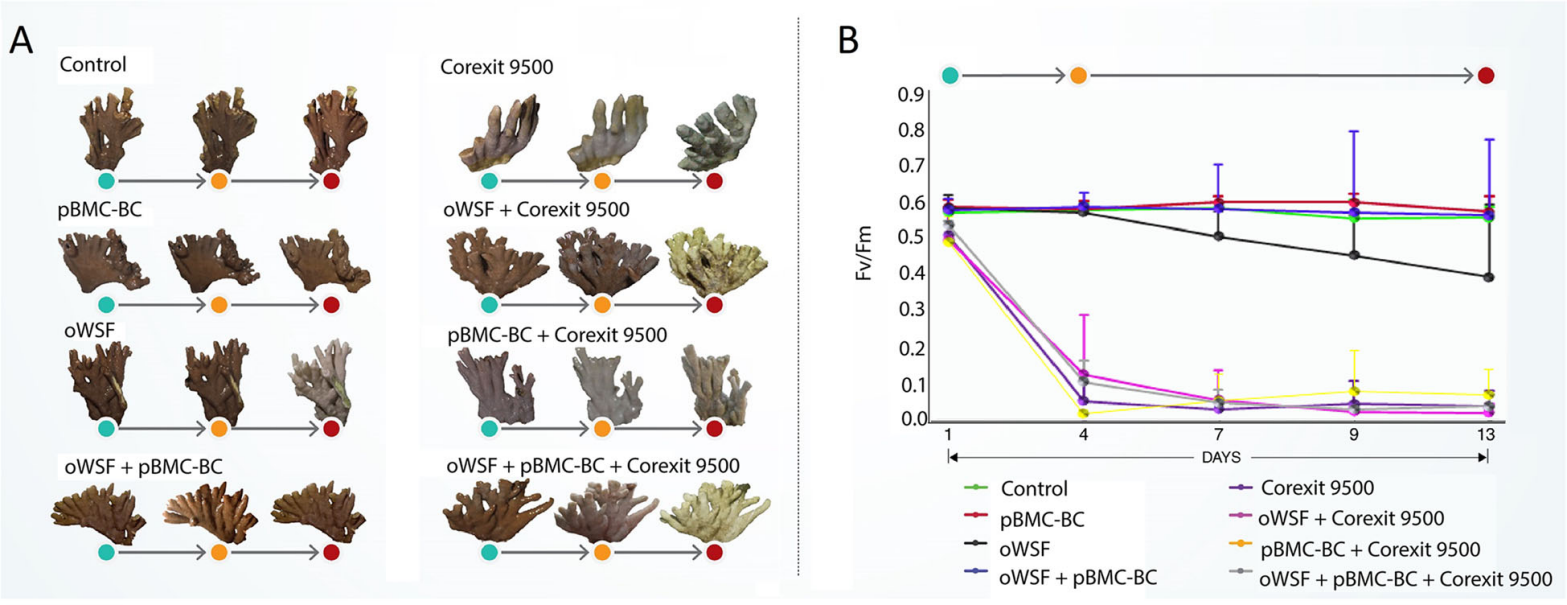
**a** Morphological changes assessed by photodocumentation in *Millepora alcicornis* fragments exposed to the treatments at *T*_0_, *T*_1_, and *T*_2_. **b** Photosynthetic efficiency measured by *F*_*v*_/*F*_*m*_ on five different days of the experiment, including *T*_0_, *T*_1_, and *T*_2_

The *M*. *alcicornis* holobiont was investigated using the maximum photosynthetic capacity of the associated Symbiodiniaceae (*F*_*v*_*/F*_*m*_) and the changes in gross morphology. At day 4 of exposure (*T*_1_), corals exposed to dispersants showed visually perceptible physiological impacts. At *T*_2_, coral fragments in treatments containing Corexit 9500 were dead, and some fragments exposed to oWSF were paler than those not exposed to this chemical dispersant (Fig. 2a; Fig. S1A and S1B). The *F*_*v*_/*F*_*m*_ data corroborated these gross morphological observations.

For corals not exposed to Corexit 9500 (including controls), the least-square means of *F*_*v*_/*F*_*m*_ values ranged from 0.55 to 0.63 ± 0.03 (mean ± standard error) among all treatments (Fig. 2b) throughout the experiment, still in the healthy range of > 0.5. The exception was the oWSF treatment without pBMC-BC, which at *T*_2_ had a mean value of *F*_*v*_/*F*_*m*_ that was 30% lower (below the healthy range of > 0.5) than those from the other non-Corexit 9500 treatments. In addition, from *T*_0_ to *T*_2_, the *F*_*v*_/*F*_*m*_ in oWSF samples decreased significantly (*p* = 0.01), while the remaining treatments without dispersant, including oWSF + pBMC-BC, did not show significant decreases.

Treatments with exposure to Corexit 9500, i.e., oWSF + Corexit 9500, pBMC-BC + Corexit 9500, and oWSF + pBMC-BC + Corexit 9500, showed *F*_*v*_/*F*_*m*_ values lower than the control (*p* < 0.0001) throughout the experiment. By *T*_2_, 88–97% of the photosynthetic efficiency was lost in the dispersant treatments relative to the control (*p* < 0.0001). Considering the treatments with Corexit 9500 as one group and the treatments without Corexit 9500 as a second group, the former had mean values of *F*_*v*_/*F*_*m*_ lower than the latter at each sampling time: 12% at *T*_0_, 87% at *T*_1_, and 92% at *T*_2_. In agreement with the *F*_*v*_/*F*_*m*_ results, gross morphological changes were observed in the dispersant treatments (Fig. 2a; Fig. S1B). At *T*_1_, corals exposed to dispersant were bleached, and by *T*_2_ many showed tissue-sloughing necrosis. Coral fragments exposed to oWSF were visually pale and negatively affected compared to the other non-dispersant treatments, but showed less visually perceptible stress, based on coral morphology and bleaching, than fragments exposed to the dispersant (Fig. S1A).

### Exposure to different treatments altered local physico-chemical conditions

Throughout the experiment, the temperature remained at 24 °C in the aquariums across all treatments. The pH differed between treatments, with Corexit 9500 treatments having lower pH levels than treatments without Corexit 9500 (*p* < 0.001; Supplementary Information SI). DOC concentrations increased across all treatments, with DOC concentrations significantly higher (*p* < 0.0001) at *T*_1_ and *T*_2_ than at *T*_0_ (Supplementary Information SI). Differences in salinity levels were significant over time (*p* < 0.0001), showing an increase by *T*_2_. Ammonium and nitrate levels decreased at *T*_2_ in both treatments (*p* < 0.0001 and *p* = 0.0024, Supplementary Information SI). Phosphate concentrations were higher at *T*_2_ than at *T*_0_ in all treatments over time (*p* < 0.001) (Table 1; Supplementary Information SI).

**Table 1:** Repeated measure ANOVA results and post-hoc means of the physicochemical conditions to all treatments throughout time

Parameters		*F* value	*p* value	Post-Hoc (Mean ± SE)
pH	Time	2.03	0.14	
	**Treatments**	**32.02**	**< 0.0001**	
	Time:Treatments	0.58	0.86	
	Post-hoc comparison			Corexit 9500 ( 8.05 ± 0.04)
				oWSF + Corexit 9500 (7.99 ± 0.04)
				oWSF + pBMC-BC + Corexit 9500 (8.06 ± 0.04)
				oWSF (8.34 ± 0.04)
				pBMC-BC (8.54 ± 0.04)
				oWSF + pBMC-BC (8.61 ± 0.04)
				Control (8.47 ± 0.04)
DOC	**Time**	**11.24**	**< 0.001**	
	Treatments	1.51	0.21	
	Time:Treatments	1.22	0.29	
	Post-hoc comparison			T0 (3.24 μg/mL ± 0.26)
				T1 (4.44 mg/mL ± 0.26)
				T2 (4.53 μg/mL ± 0.26)
Salinity	**Time**	**78.9**	**< 0.0001**	
	**Treatments**	**2.8**	**0.03**	
	Time:Treatments	1.6	0.11	
	Post-hoc comparison			T0 (36.22 mg/L ± 0.07)
				T1 (36.17 mg/L ± 0.07)
				T2 (37.25 mg/L ± 0.07)
				Control (36.70 mg/L ± 0.12)
				oWSF (36.42 mg/L ± 0.12)
				pBMC-BC (36.74 mg/L ± 0.12)
				Corexit 9500 (36.60 mg/L ± 0.12)
				oWSF + pBMC-BC (36.20 mg/L ± 0.14)
				oWSF + Corexit 9500 (36.38 mg/L ± 0.12)
				pBMC-BC + Corexit 9500 (36.85 mg/L ± 0.12)
				oWSF + pBMC-BC + Corexit 9500 (36.47 mg/L ± 0.12)
Ammonium	**Time**	**14.86**	**< 0.0001**	
	Treatments	1.33	0.28	
	Time:Treatments	1.07	0.41	
	Post-hoc comparison			T0 (28.10 μg/L ± 19.22)
				T1 (160.09 μg/L ± 19.22)
				T2 (99.83 μg/L ± 19.22)
Nitrate	**Time**	**6.88**	**0.002**	
	Treatments	0.35	0.92	
	Time:Treatments	0.72	0.74	
	Post-hoc comparison			T0 (6.56 μg/L ± 10.76)
				T1 (58.11 μg/L ± 10.76)
				T2 (34.65 μg/L ± 10.76)
Phosphate	**Time**	**20.21**	**< 0.0001**	
	**Treatments**	**5.59**	**< 0.001**	
	**Time:Treatments**	**3.37**	**< 0.001**	
	Post-hoc comparison			Control in ***T***_**0**_ (18.65 μg/L ± 23.19), ***T***_**1**_ (61.80 μg/L ± 23.19) and ***T***_**2**_ (33.82 μg/L ± 23.19)
				oWSF in ***T***_**0**_ (13.96 μg/L ± 23.19), ***T***_**1**_ (25.24 μg/L ± 23.19) and ***T***_**2**_ (17.26 μg/L ± 23.19)
				pBMC-BC in ***T***_**0**_ (5.97 μg/L ± 23.19), ***T***_**1**_ (51.55 μg/L ± 23.19) and ***T***_**2**_ (70.67 μg/L ± 23.19)
				Corexit 9500 in ***T***_**0**_ (3.22 μg/L ± 23.19), ***T***_**1**_ (234.27 μg/L ± 23.19) and ***T***_**2**_ (136.47 μg/L ± 23.19)
				oWSF + pBMC-BC in ***T***_**0**_ (6.76 μg/L ± 23.19), ***T***_**1**_ (56.20 μg/L ± 23.19) and ***T***_**2**_ (97.50 μg/L ± 23.19)
				oWSF + Corexit 9500 in ***T***_**0**_ (0 μg/L ± 23.19), ***T***_**1**_ (83.35 μg/L ± 23.19) and ***T***_**2**_ (53.00 μg/L ± 23.19)
				pBMC-BC + Corexit 9500 in ***T***_**0**_ (3.22 μg/L ± 23.19), ***T***_**1**_ (3.77 μg/L ± 23.19) and ***T***_**2**_ (109.82 μg/L ± 23.19)
				oWSF + pBMC-BC + Corexit 9500 in ***T***_**0**_ (3.77 μg/L ± 23.19), ***T***_**1**_ (78.75 μg/L ± 23.19) and ***T***_**2**_ (34.20 μg/L ± 23.19)
PAH	**Time**	**7.86**	**0.001**	
	**Treatments**	**20.86**	**< 0.0001**	
	**Time:Treatments**	**3.31**	**0.001**	
	Post-hoc comparison			Control in ***T***_**0**_ (1.63 μg/L ± 467.05), ***T***_**1**_ (0.48 μg/L ± 467.05) and ***T***_**2**_ (0.58 μg/L ± 467.05)
				oWSF in ***T***_**0**_ **(**4.36 μg/L ± 467.05), ***T***_**1**_ (0.61 μg/L ± 467.05) and ***T***_**2**_ (1.26 μg/L ± 467.05)
				pBMC-BC in ***T***_**0**_ (0.08 μg/L ± 467.05), ***T***_**1**_ (0.06 μg/L ± 467.05) and ***T***_**2**_ (0.10 μg/L ± 467.05)
				Corexit 9500 in ***T***_**0**_ (17.72 μg/L ± 467.05), ***T***_**1**_ (5.99 μg/L ± 467.05) and ***T***_**2**_ (4.99 μg/L ± 467.05)
				oWSF + pBMC-BC in ***T***_**0**_ (3.39 μg/L ± 539.30), ***T***_**1**_ (0.03 μg/L ± 539.30) and ***T***_**2**_ (0.14 μg/L ± 539.30)
				oWSF + Corexit 9500 in ***T***_**0**_ (803.19 μg/L ± 467.05), ***T***_**1**_ (4665.46 μg/L ± 467.05) and ***T***_**2**_ (2711.75 μg/L ± 467.05)
				pBMC-BC + Corexit 9500 in ***T***_**0**_ (8.60 μg/L ± 467.05), ***T***_**1**_ (9.39 μg/L ± 467.05) and ***T***_**2**_ (3.94 μg/L ± 467.05)
				oWSF + pBMC-BC + Corexit 9500 in ***T***_**0**_ (1162.94 μg/L ± 467.05), ***T***_**1**_ (4559.76 μg/L ± 467.05) and ***T***_**2**_ (2165.55 μg/L ± 467.05)
N-alkanes	**Time**	**4.84**	**0.013**	
	**Treatments**	**9.98**	**< 0.0001**	
	**Time:Treatments**	**2.21**	**0.022**	
	Post-hoc comparison			Control in ***T***_**0**_ (1.63 μg/L ± 467.05), ***T***_**1**_ (0.48 μg/L ± 467.05) and ***T***_**2**_ (0.58 μg/L ± 467.05)
				oWSF in ***T***_**0**_ **(**4.36 μg/L ± 467.05), ***T***_**1**_ (0.61 μg/L ± 467.05) and ***T***_**2**_ (1.26 μg/L ± 467.05)
				pBMC-BC in ***T***_**0**_ (0.08 μg/L ± 467.05), ***T***_**1**_ (0.06 μg/L ± 467.05) and ***T***_**2**_ (0.10 μg/L ± 467.05)
				Corexit 9500 in ***T***_**0**_ (17.72 μg/L ± 467.05), ***T***_**1**_ (5.99 μg/L ± 467.05) and ***T***_**2**_ (4.99 μg/L ± 467.05)
				oWSF + pBMC-BC in ***T***_**0**_ (3.39 μg/L ± 539.30), ***T***_**1**_ (0.03 μg/L ± 539.30) and ***T***_**2**_ (0.14 μg/L ± 539.30)
				oWSF + Corexit 9500 in ***T***_**0**_ (803.19 μg/L ± 467.05), ***T***_**1**_ (4665.46 μg/L ± 467.05) and ***T***_**2**_ (2711.75 μg/L ± 467.05)
				pBMC-BC + Corexit 9500 in ***T***_**0**_ (8.60 μg/L ± 467.05), ***T***_**1**_ (9.39 μg/L ± 467.05) and ***T***_**2**_ (3.94 μg/L ± 467.05)
				oWSF + pBMC-BC + Corexit 9500 in ***T***_**0**_ (1162.94 μg/L ± 467.05), ***T***_**1**_ (4559.76 μg/L ± 467.05) and ***T***_**2**_ (2165.55 μg/L ± 467.05)

**Fig. 3:**
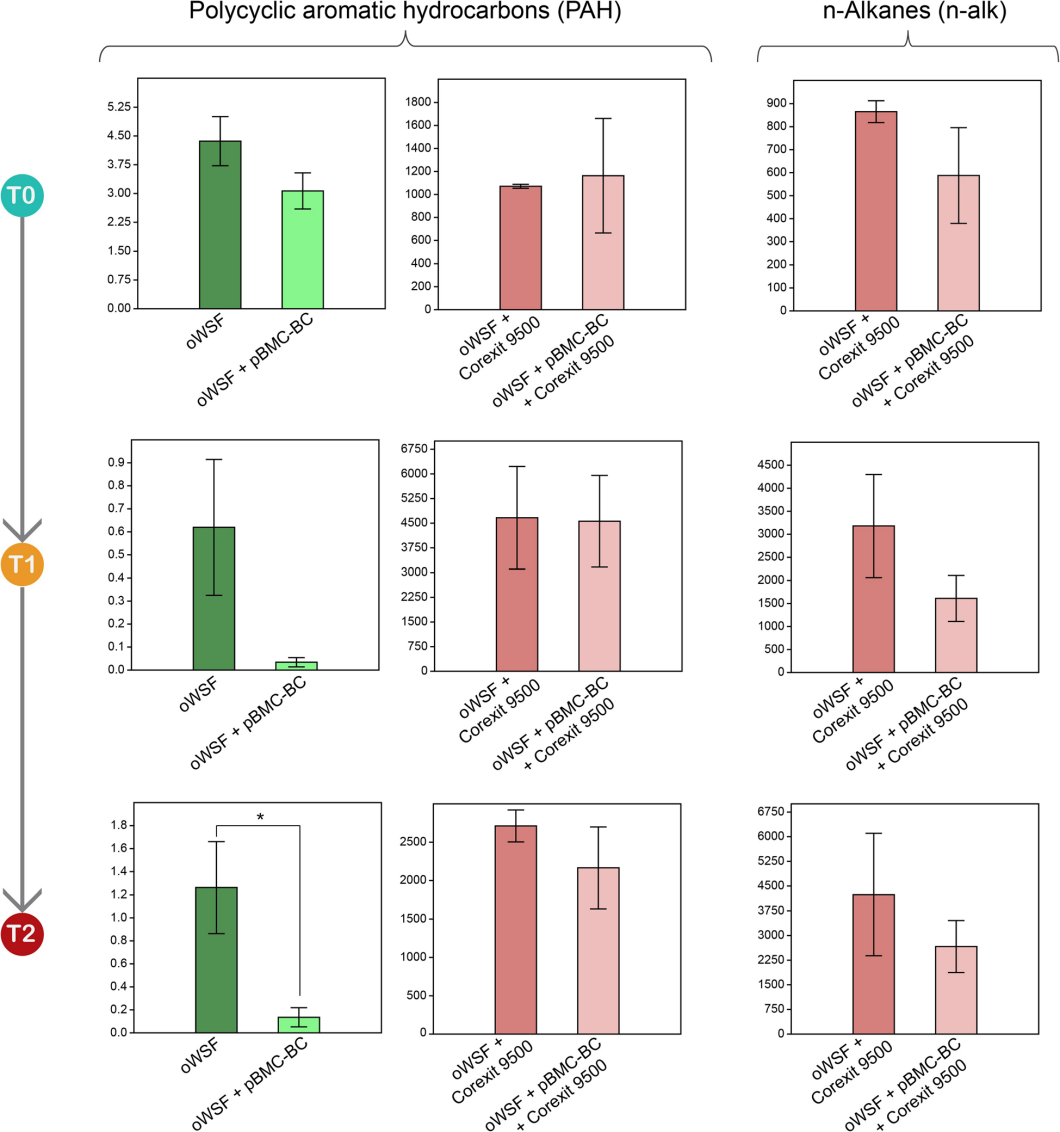
Quantification of PAH and *n*-alkanes in samples where they were detectable, at *T*_0_, *T*_1_, and *T*_2_. In all treatments not shown (including the control treatment), PAH and *n*-alkanes were undetectable

Levels of PAHs increased significantly in the treatments with oil and dispersants (both oWSF + Corexit 9500 and oWSF + pBMC-BC +Corexit 9500). In the treatments with oil but without dispersant (oWSF and oWSF + pBMC-BC), PAH levels at *T*_2_ were significantly lower in the treatments containing pBMC-BC than with oil only (*p* = 0.032) at *T*_2_, indicating the degradation efficiency of the consortium (see Supplementary Information SI). N-alkane levels were detectable only in oWSF + Corexit 9500 and oWSF + pBMC-BC + Corexit 9500. The concentration of *n*-alkane in both treatments increased at *T*_2_ compared to *T*_0_ (*p* = 0.013; Supplementary Information SI). However, at time *T*_2_, *n*-alkanes in the treatments with pBMC-BC inoculation were 38% lower than in the treatment containing dispersant and oil only. The repeated-measure ANOVA results and post-hoc comparisons of the physico-chemical conditions are shown in Table 1, and a summary of oil hydrocarbon that could be quantified is shown in Fig. 3.

### Exposure to different treatments alters coral microbiome

The 16S amplicon analysis assessed a total of 397,454 sequences that clustered into 5986 OTUs (within the 3% dissimilarity threshold). The rarefaction curve approached an asymptote (Fig. S2), with most samples showing coverage values between 96.75 and 98.75%, which indicates a satisfactory sampling effort for this community (Fig. S3). However, part of the rare biosphere would be better comprehended with a greater sequencing depth.

Species diversity, represented by the Shannon index, did not differ significantly among the treatments over time (Fig. S4). The Chao index showed decreased richness from *T*_0_ to *T*_1_ in all treatments, except in the Corexit 9500 treatment, which increased (Fig. S5). However, the Chao index was lower in oWSF than in oWSF + pBMC-BC. β-diversity analyses indicated that Corexit 9500 had a significant effect (three-way PERMANOVA, *p* < 0.05) on the structure of the bacterial community. In addition, microbial communities exposed to the dispersant were significantly different (three-way PERMANOVA, *p* < 0.05) between *T*_0_ and *T*_1_ and between *T*_0_ and *T*_2_ (pairwise three-way PERMANOVA, *p* < 0.001) (Tables S2 and S3).

**Fig. 4:**
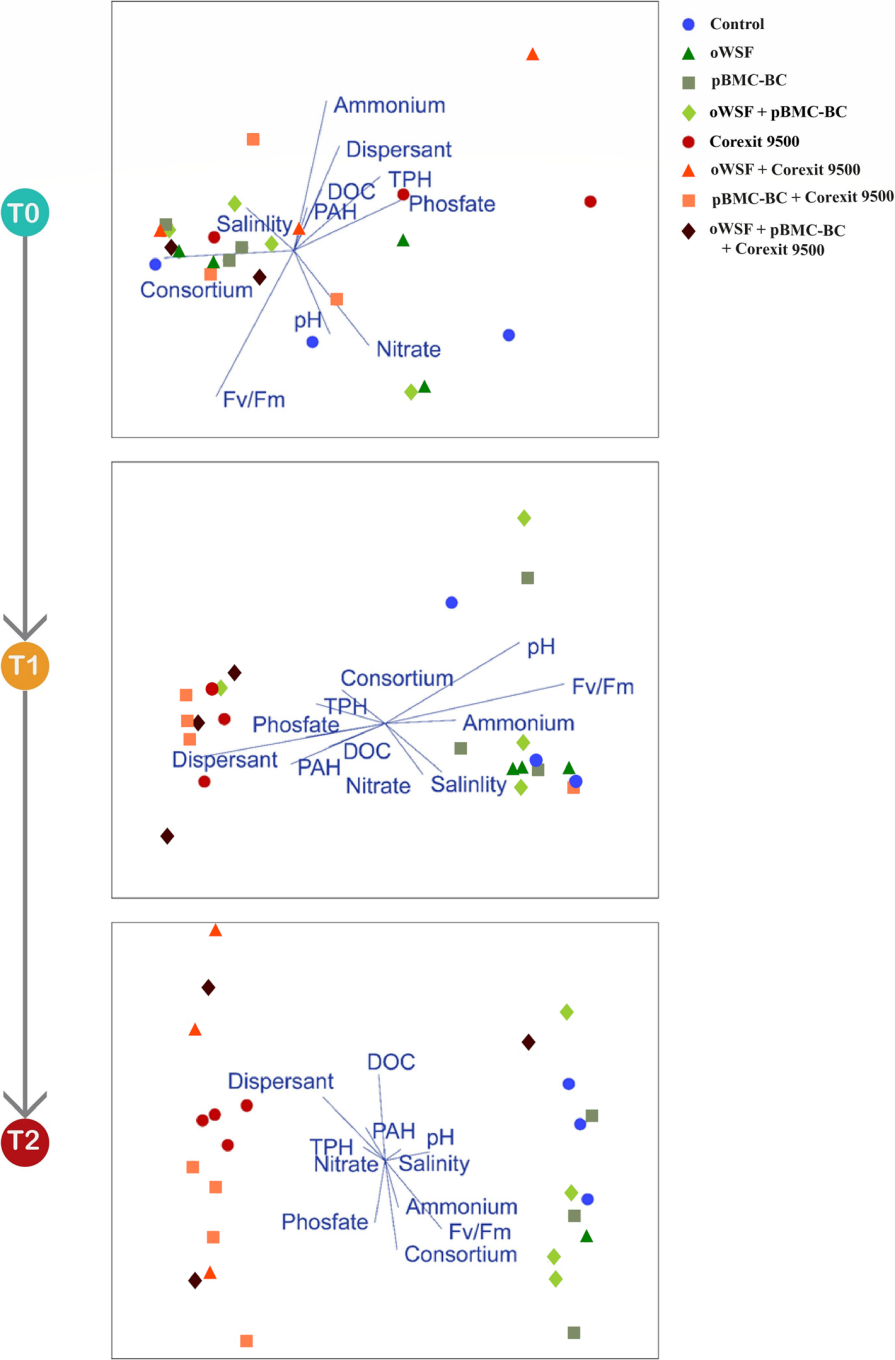
Non-metric multidimensional scaling (nMDS) ordination of the *Millepora alcicornis* bacterial community composition in the different treatments over time, using the Bray-Curtis similarity coefficient based on OTU distribution. The angles and lengths of radiating blue lines indicate the direction and strength of the relationships between the abiotic variables and the ordination scores

**Fig. 5:**
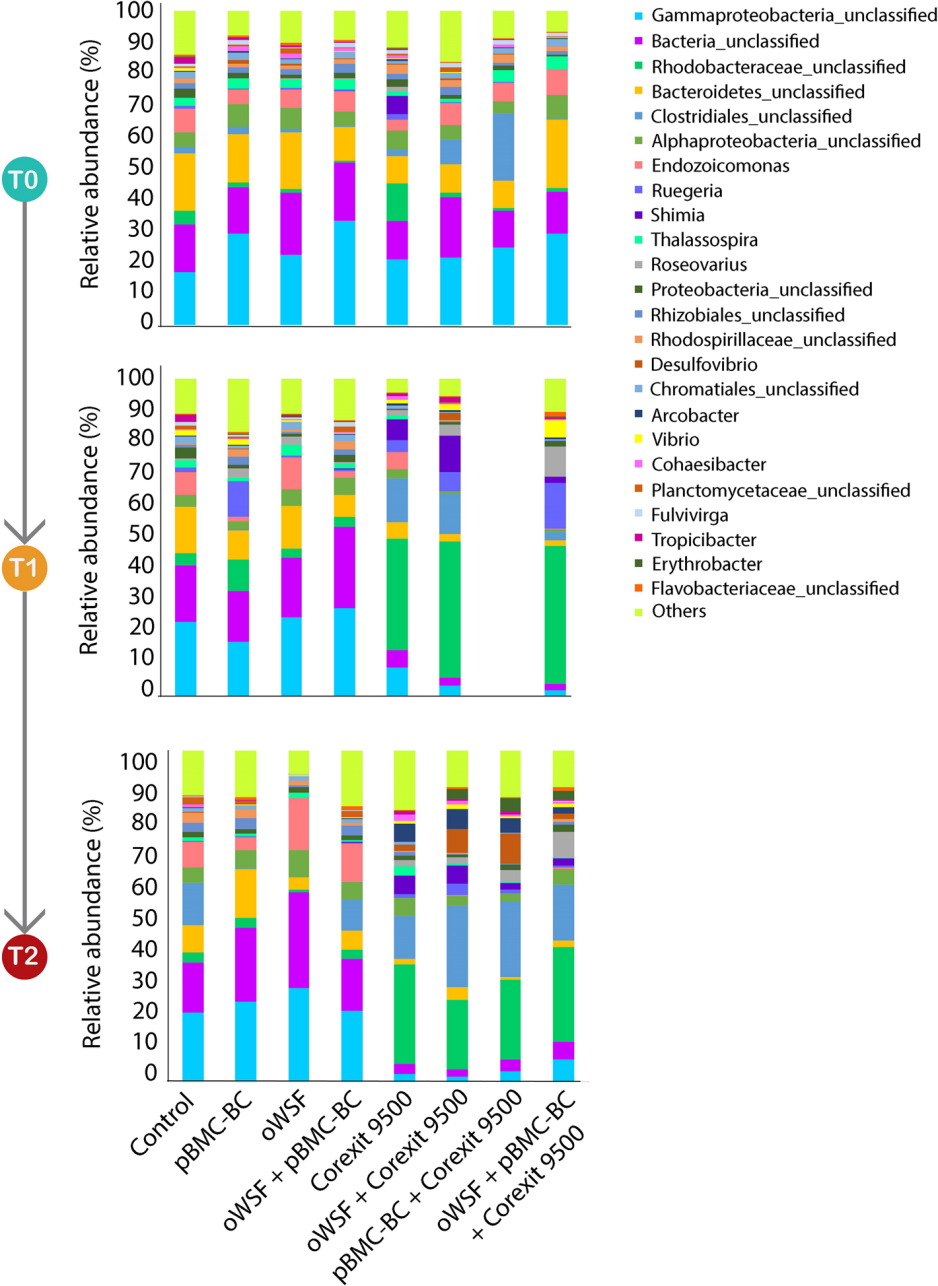
Taxonomic comparison of the bacterial genera based on the DNA sequences obtained from the partial sequence of the 16S subunit of ribosomal RNA in all treatments over time. Note: the pBMC-BC + Corexit9500 sample in T1 is missing, due to the low-quality sequences

The impact of the chemical dispersant on the community structure was also corroborated by the NMDS analysis, which showed that two distinct bacterial community clusters formed over time: one in the treatments with dispersant and another in the treatments without dispersant (Fig. 4). The bacterial community profile was also analyzed, based on the relative abundances in each treatment over time. We identified 17 bacterial phyla and 27 classes associated with the *M*. *alcicornis* samples. At the genus level, all treatments had a similar bacterial community. Beginning with *T*_1_, these profiles changed between the treatments with and without Corexit 9500. *Endozoicomona*s sp. and *Thalassospira* sp. were present in the treatments without Corexit, whereas unclassified Rhodobacteraceae and unclassified Clostridiales, *Desulfovibrio* sp., *Roseovarius* sp., and *Shimia* sp. were present in the treatments with Corexit and increased in relative abundance over time (Fig. 5).

The phylum Proteobacteria dominated in all treatments (Fig. S6). Classes Alpha- and Gammaproteobacteria dominated in all treatments at *T*_0_, but Gammaproteobacteria became more abundant than Alphaproteobacteria in the treatments without Corexit, varying between 23–46% and 15–21% over time, respectively (Fig. S7). We cannot confirm that pBMC-BC isolates were present at the order level, but it is possible to infer that the pBMC-BC was represented at this level through the presence of Oceanospirillales, Alteromonadales, and Rhizobiales. These orders maintained their relative abundances in the treatments with pBMC-BC and decreased in abundance in the treatments with dispersant over time (Fig. S8).

**Fig. 6:**
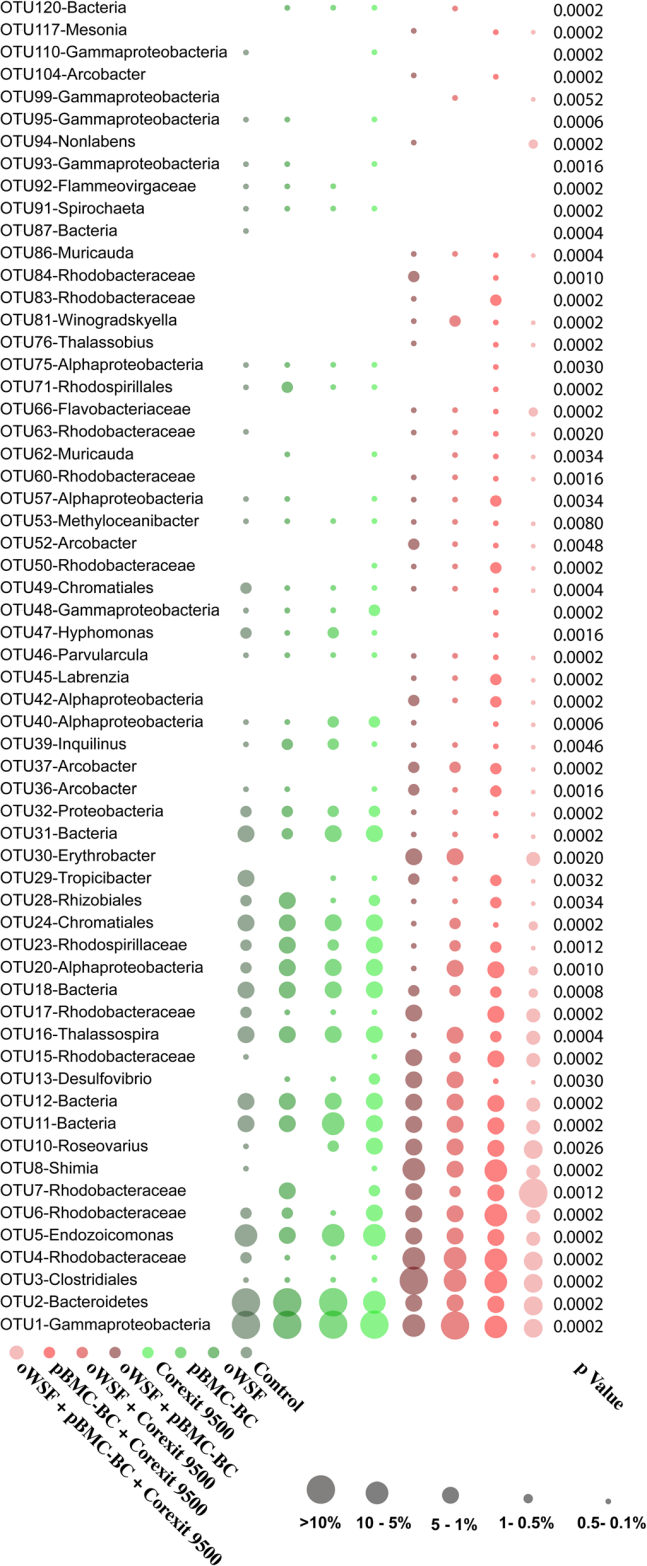
Bubble graph of relative abundances of indicator OTUs in each treatment. The percentage of relative abundance is shown below the graph for all indicator OTUs. Bubbles shown for statistically significant OTUs (*p* < 0.05) and with indicator value > 60

Analysis of indicator species showed that some OTUs were associated with pBMC-BC, oWSF, or Corexit, with 95 significant OTUs identified (*p* < 0.05) (Fig. 6). Among the three factors, the largest number of potential indicator bacteria was found in the dispersant samples.

## Discussion

Water pollution is one of the three main causes of reef loss globally [23]. Local management to minimize stressors can increase the ability of corals to cope with global impacts by reducing the synergistic effects caused by several stressors [48, 49]. To this end, the United Nations recently emphasized the need to reduce marine pollution and protect and restore coral ecosystems in the “Global Goals for Sustainable Development”  [50]. Recently, a committee of the National Academies of Sciences, Engineering, and Medicine reviewed possible local and global interventions to increase the resilience of coral reefs  [51]. Among these interventions, the manipulation of beneficial microorganisms  [21] and the development of pollution remediation approaches were listed as possible strategies to help coral persistence.

Bioremediation methods have advantages compared with other oil cleanup techniques, which include sustainability, lower costs, and applicability across different ecosystems with minimal impacts  [32, 52, 53]. The use of oil-degrading bacteria to remediate oil contamination may have benefits in addition to the degradation of compounds. For instance, Santos et al. [32] successfully minimized the toxicity of oil to the coral *Mussismilia harttii* with a bacterial probiotic consortium. Probiotics were initially defined as live microbes that can benefit human health [54]. This definition was later extended to include any host system, including corals [55]. One of the benefits provided by microbial probiotics is neutralization of toxic compounds [56, 57], protecting the hosts against their harmful effects. Therefore, the use of coral-associated microbes to mitigate oil contamination and its consequent impact on coral health can be considered a probiotic approach. However, uptake of a specific inoculated oil-degrading strain by corals is not crucial for defining it as a probiotic, since oil is often degraded in the surrounding water. Our results showed that, although the pBMC-BC consortium members could not be detected in the coral-microbiome assays, inoculation of the consortium was able to mitigate the negative physiological effects observed from the application of oWSF, as indicated by our indirect proxy (*F*_*v*_/*F*_*m*_ rates) and visible physiological responses (death and bleaching).

The use of coral-associated microbial consortia has proven to benefit coral health in the presence of oil [32], marine pathogens, and increased temperatures [33]. The application of the multi-domain consortium resulted in degradation of *n*-alkanes and significant decrease of PAH hydrocarbon fractions. A specific strain of microorganism is usually unable to degrade several different hydrocarbon fractions of oil; rather, hydrocarbon degradation is more efficient when there is a set of microorganisms that degrade certain components [58]. This study provides evidence that a multi-domain consortium isolated from the coral microbiome was efficient in degrading different oil fractions. Furthermore, the detected oil degradation was associated with improved direct and indirect coral health metrics at the last sampling time of the experiment, compared to samples without the pBMC-BC inoculation.

The corals were, however, severely affected by Corexit 9500, in spite of the application of the beneficial consortium or the concomitant exposure to oil. Measurements of *F*_*v*_/*F*_*m*_ revealed a separation into two main groups, those containing the dispersant and those without it. This information, together with photodocumentation of dead, bleached, or damaged tissue in the presence of Corexit 9500, showed that the dispersant damaged the animals shortly after application in the experimental conditions (Fig. 2a, b).

In addition, this study demonstrated that exposure to Corexit 9500 caused a significant change in the associated bacteria community of calcifying cnidarians, which occurred in parallel with a negative impact on the host physiology. This new information on the effect of Corexit on the associated bacterial community of a marine calcifying organism adds to the list of known harmful effects of chemical dispersants on the physiology of several species from different ecosystems [20, 59, 60]. In corals, the damage ranges from obvious effects such as bleaching and tissue necrosis [20, 61] to more subtle consequences such as inhibition of fertilization and larval metamorphosis [62], both of which affect species perpetuation. Here, we observed these effects in a realistic, open-system experiment, and also revealed one more “invisible” impact that directly affects coral health: the effect of Corexit 9500 on the associated microbiome.

Over 200 microbial genera have been reported as able to facultatively degrade petroleum hydrocarbons [63]. Among these, the genera *Roseovarius* and *Erythrobacter* increased in abundance in the presence of dispersant. However, the presence of the dispersant also reduced the abundance of some other oil-degrading bacteria, such as *Thalossospira* and *Hyphomonas* [64–66] (Fig. 5).

This last genus, *Hyphomonas*, was also found to be a potential bioindicator of the presence of oil (Fig. 6). Different oil-degrading bacteria occurred in both the presence and absence of Corexit 9500, making it unclear whether the dispersant is affecting the capacity of the microbial population to remediate oil under the tested circumstances. In previous studies, chemical dispersants not only proved ineffective in promoting oil degradation but also retarded biodegradation [67].

Microorganisms have been used as bioindicators of different pollutants in marine ecosystems [32, 68]. The presence of dispersants also increased the number of bacteria that were found to be related to diseased and stressed corals. For instance, the genus *Ruegeria*, previously reported as associated with diseased [69] and stressed [70] corals, increased in the treatments containing dispersants. Additionally, members of the genus *Roseovarius*, which are also associated with diseased  [71–73] and stressed [70] corals, increased in the presence of dispersant over time (Figs. 5, 6 and S7). Other bioindicators of dispersants include *Shimia*, *Thalassobius*, *Erythrobacter*, and *Desulfovibrio*, all found to be related to diseased and stressed corals [70, 72, 74–76]. Taken together, these results suggest that disruption of the beneficial interactions of the associated microbial community could weaken the host, through an increase of commensal and opportunistic microbes, or as an immediate consequence of exposure to the dispersant.

At the family level, an OTU closely related to a member of Flavobacteriaceae was one of the dispersant bioindicators. Our data agree with the findings of McFarlin et al. [77], which showed that the family Flavobacteriaceae was enriched in the presence of Corexit, and therefore an indicator of Corexit [77]. Members of this family include well-known opportunistic and pathogenic species [78, 79] and are often overabundant in corals exposed to several stress factors [80, 81]. Through our results and reports in the literature, we can predict that Flavobacteriaceae may have been one of the groups of microorganisms involved in the initial process of dysbiosis leading to the death of the coral.

Although not classified as bioindicators, *Vibrio* OTUs increased in relative abundance in the presence of Corexit 9500 over time, which can be explained by the ability of some *Vibrio* species to metabolize dispersants [82]. Additionally, several species of *Vibrio* are pathogenic and opportunistic bacteria with many different groups of hosts, attacking humans, plants, and corals, among others [83]. They have many lysogenic islands that can be transferred horizontally intra- and inter-specifically  [84, 85], and their virulence can increase in stress conditions, such as a temperature increase [86]. In corals, *Vibrio* species are associated with several diseases [87–89]. We also observed an increase in members of the genus *Vibrio* after the exposure to Corexit 9500; these were the most abundant isolates in the presence of the dispersant [90]. These results suggest that chemical dispersants may affect coral health not only through their toxicity itself, but may also increase the abundance of opportunistic or pathogenic bacteria (i.e., members of the genus *Vibrio*), which may cause dysbiosis and disease.

 Despite the fact that most of the bioindicators of Corexit 9500 have been described as opportunistic pathogens, one dispersant bioindicator, a member of the genus *Labrenzia*, has been previously reported as showing potential beneficial characteristics for corals by producing antimicrobial compounds [91]. The genome analysis of a *Labrenzia* strain associated with coral revealed 4 halo acid dehalogenase-encoding genes and one haloalkane dehalogenase-encoding gene, which can be used to degrade a broad range of aromatic halogens, haloalcohols, and halo acids [92]. Identification of members of this genus as dispersant bioindicators may be useful in further development of BMCs specifically selected to protect corals against Corexit 9500. Species of this genus are both potential BMCs and oil degraders, which makes them candidates for future experiments on cleanup of petroleum contamination close to coral reefs.

On the other hand, bacteria previously correlated with healthy corals were also found to be bioindicators of the absence of dispersants, meaning that they were severely affected by the presence of Corexit 9500. Examples are a member of the genus *Thalassospira*,  previously correlated with healthy coral hosts  [93], and potentially involved with the phosphorus cycle [94]*, Parvularcula*, also associated with healthy corals [95], as well as the genus *Inquilinus*,  reported as important for heat tolerance in corals [96]. The well-known coral symbiont *Endozoicomonas* was also negatively affected by the presence of chemical dispersants. Members of this genus have been frequently associated with healthy corals [97–99] , and the different strain genomes revealed functional adaptation and plasticity [100], suggesting that the relationship between this bacterial genus and the host is important to the adaptation and survival of the holobiont.

This study addressed the impact of oil and Corexit 9500 on the coral physiology and microbiome, as well as the development of a bioremediation strategy that avoids the use of chemical dispersants in reef areas. As expected, the presence of Corexit 9500 impacted both the physiology and microbiome of the host shortly after application. In contrast, even though exposure to oil also impacted the coral health and physiology, it did not significantly change the microbiome structure. This result suggests that the natural microbiome of corals may be resilient to oil contamination up to a certain level, even when physiological parameters on the host side are affected. Previous research has shown that some resident coral-associated bacteria have the ability to degrade oil using it as a carbon source [101]. When confronted with an oil spill, oil-degrading bacteria can increase in abundance, as seen in deep-sea coral reefs impacted by the Deepwater Horizon oil spill [102]. The association with oil-degrading bacteria may be exploited as an important adaptation tool for the coral holobiont in areas experiencing oil spills, as it may increase the survivability of exposed corals [103]. Thus, administration of the multi-domain pBMC-MC consortium may further contribute to this adaptive response by increasing the abundance of oil-degrading bacteria in the holobiont. Indeed, the multi-domain pBMC-BC consortium was able to protect the corals from the negative effects of oil exposure, by increasing oil degradation and consequently improving host health, as measured by the Fv/Fm indirect health proxy and morphological traits. These results support the hypothesis that the bioremediation consortium could assist not only in degrading the oil in the water but also in maintaining the resilience of the natural coral microbiome against occasional oil spills. Thus, application of a multi-domain biodegrading consortium as an oil-spill response technique could be a useful alternative to dispersants, since it could provide two advantages: (1) filling the niche with probiotics that can prevent pathogenic organisms from colonizing coral reefs, and (2) helping to reduce hydrocarbon concentrations and their potential impacts on corals. As our data showed, physiological improvements to coral health can be achieved via a multi-domain consortium without causing major changes to the coral microbiome. It may be that the chosen microbial consortia consisted of microbes that are part of the rare biosphere which can perform critical functions, such as degrading oil without having high relative abundances, which has also been shown to occur in coastal seawater samples [104, 105]. Inoculation of probiotics can also contribute to the establishment and succession of other beneficial microbes, as demonstrated through the use of pre- and probiotics in humans [106, 107]. As the field of environmental probiotic research continues to grow, these rare taxa may be key in understanding how best to implement coral probiotics in the field without causing long-term changes to reef microbial communities.

Another possible reason for the low abundance of the consortium members is that, although the members do not increase in abundance in the holobiont tissue, they may have increased in abundance in the surrounding water. However, this hypothesis is merely speculative. Further studies should also include the characterization of microbial abundance in the surrounding water using metagenomics and metatranscriptomics approaches, along with more-detailed analysis and quantification of hydrocarbons. Through these approaches, we can address yet-unanswered questions, allowing us to better understand the molecular mechanisms and ecological principles underpinning the beneficial effects of these microbial consortia on the holobiont. In view of the correlation between inoculation and improved coral health, in the future we will refer to them as BMC-BCs and not pBMC-BC.

Currently, there are no known negative effects of readministering native beneficial bacteria back into a coral reef system to combat stress conditions. Nevertheless, many things remain to be learned about environmental probiotics and their application in natural systems. For instance, various obstacles must be overcome to make BMCs applicable and effective at large scale. Among these are consortium large-scale production and optimization; bioproduct maintenance during storage; delivery alternatives compatible with the actual conditions of offshore application; and logistical concerns, some of them extensively discussed by Peixoto and colleagues [49]. These challenges will be further addressed based on the results of ongoing studies. Ideally, before applying these developed technologies in the field, long-term experiments using realistic mesocosm systems, such as the present one, would be used to test their efficiency and map any potential risks. However, as time is increasingly short, urgent interventions must be put into practice, and the use of Beneficial Microorganisms for Corals (BMCs) is considered an extremely promising alternative.

The persistence of coral reefs depends on many changes that are needed in the near future. The scientific community and environmental organizations must try to minimize the local and global impacts that affect reef survival. Coral reefs in the South Atlantic, considered major reef refuges [108], are currently experiencing unprecedented impacts, resulting in mass die-offs in this area [109]. A recent mass-mortality event affected about 90% of the fire coral *M*. *alcicornis* at one site [109]. Investigation of the *M*. *alcicornis* microbiome and selection of probiotics that can help to mitigate the effects of oil spills and other stressors can contribute to the protection of this important, and now potentially threatened, reef builder in the South Atlantic. This study examined the response of the coral microbiome to exposure to a chemical dispersant, furthering the understanding of ecological interactions—such as symbiosis and pathogenicity—between the host and its associated microbes under adverse stress conditions. Innovative actions in environmentally friendly strategies to mitigate marine oil pollution without causing side effects are insufficient [15] but are still needed. Our results and other studies in this field can contribute immensely to inform local actions to protect coral reefs in the Anthropocene, such as the mass die-offs caused by global change.

## Conclusions

Our study concluded that the chemical dispersant Corexit 9500 was far more toxic to *M*. *alcicornis* than the oil itself, in a flow-through experiment simulating realistic conditions. This study can help companies and governmental agencies in their decision-making about the use of chemical or biological remediation, since we showed that BMC-BC minimizes the negative oil effects without being toxic to the coral.

This is also the first study to explore the effects of Corexit 9500 on the microbiome of calcifying cnidarians. Our results showed that Corexit 9500 caused a significant shift in the bacterial community associated with the hydrocoral *M*. *alcicornis*.

In addition, this study is a proof-of-concept that multi-domain BMC-BCs consortia can be used to mitigate the impacts of oil on coral reefs and adjacent areas. The results emphasize the importance of investigating the host-associated microbiome to protect corals from anthropogenic impacts, as well as the possibility of using beneficial microbes as a tool for conservation purposes.

### Supplementary Information

**Additional file 1.** Statistical analyses of all parameters, performed using R software.

**Additional file 2: Figure S1.** Photographs of all replicates for each treatment at T_0_, T_1_ and T_2_ on Coral Watch Health Card. A) Treatments without Corexit 9500; B) Treatments with Corexit 9500.

**Additional file 3: Figure S2.** Rarefaction curve representing the α-diversity analysis of the bacterial community, based on the partial sequences of 16S subunits of ribosomal RNA in all treatments over time.

**Additional file 4: Figure S3.** Boxplot showing the percentage of sequences coverage value from the bacterial community in all treatments.

**Additional file 5: Figure S4.** Estimation of OTU richness through the Shannon diversity index, in all treatments over the experimental period.

**Additional file 6: Figure S5.** Estimation of OTU richness through the Chao diversity index, in all treatments over the experimental period.

**Additional file 7: Figure S6.** Taxonomic comparison of bacterial phyla, based on the DNA sequences obtained from the partial sequence of the 16S subunit of ribosomal RNA in all treatments over time. Note: the pBMC-BC+Corexit9500 sample in T1 is missing, due to the loss of low-quality sequences.

**Additional file 8: Figure S7.** Taxonomic comparison of bacterial classes, based on the DNA sequences obtained from the partial sequence of the 16S subunit of ribosomal RNA in all treatments over time. Note: the pBMC-BC+Corexit9500 sample in T1 is missing, due to the loss of low-quality sequences.

**Additional file 9: Figure S8.** Taxonomic comparison of bacterial orders, based on the DNA sequences obtained from the fragment of the 16S subunit of ribosomal RNA in all treatments over time. Note: the pBMC-BC+Corexit9500 sample in T1 is missing, due to the loss of low-quality sequences.

**Additional file 10: Table S1.** Details of the conditions and sources used to isolate consortium members, as well as the NCBI deposit identification numbers.

**Additional file 11: Table S2.** Results of the three-way PERMANOVA test of β-diversity analyses conducted for all treatments and time periods of the experiment.

**Additional file 12: Table S3.** Pairwise three-way PERMANOVA test of β-diversity analyses, comparing all time periods of the experiment.

**Additional file 13.**

## Data Availability

All rRNA sequences or individual isolates were deposited in GenBank under the accession numbers shown in Table S1. All raw data for 16S rRNA amplicon sequencing are available from the BioSample database (Accession number SUB6046921). The 16S rRNA gene dataset is available under BioProject ID PRJNA556854 (https://www.ncbi.nlm.nih.gov/bioproject/PRJNA556854).

## Abbreviations

BMC: Beneficial microorganisms for corals
pBMC-BC: Putative BMC-bioremediator consortium
BMC-BC: BMC-bioremediator consortium
oWSF: Water-soluble fraction
oWIF: Water-insoluble fraction
BH: Bushnell-Haas
OD: Optical density
CFU: Colony-forming units
MA: Marine agar
MB: Marine broth
DOC: Dissolved organic carbon
PAH: Polycyclic aromatic hydrocarbon
OTU: Operative taxonomic unit
NMDS: Non-parametric multidimensional scaling

## References

[1] Braverman I (2018). Coral Whisperers: Scientists on the Brink.

[2] Hughes TP, Anderson KD, Connolly SR, Heron SF, Kerry JT, Lough JM, Baird AH, Baum JK, Berumen ML, Bridge TC, Claar DC, Eakin CM, Gilmour JP, Graham NAJ, Harrison H, Hobbs JPA, Hoey AS, Hoogenboom M, Lowe RJ, McCulloch MT, Pandolfi JM, Pratchett M, Schoepf V, Torda G, Wilson SK (2018). Spatial and temporal patterns of mass bleaching of corals in the Anthropocene. Science..

[3] Douglas AE (2003). Coral bleaching––how and why?. Mar Pollut Bull..

[4] Glynn PW (1993). Coral reef bleaching: ecological perspectives. Coral Reefs..

[5] Hughes TP, Kerry JT, Álvarez-Noriega M, Álvarez-Romero JG, Anderson KD, Baird AH, Babcock RC, Beger M, Bellwood DR, Berkelmans R, Bridge TC, Butler IR, Byrne M, Cantin NE, Comeau S, Connolly SR, Cumming GS, Dalton SJ, Diaz-Pulido G, Eakin CM, Figueira WF, Gilmour JP, Harrison HB, Heron SF, Hoey AS, Hobbs JPA, Hoogenboom MO, Kennedy EV, Kuo CY, Lough JM, Lowe RJ, Liu G, McCulloch MT, Malcolm HA, McWilliam MJ, Pandolfi JM, Pears RJ, Pratchett MS, Schoepf V, Simpson T, Skirving WJ, Sommer B, Torda G, Wachenfeld DR, Willis BL, Wilson SK (2017). Global warming and recurrent mass bleaching of corals. Nature..

[6] Pandolfi JM, Connolly SR, Marshall DJ, Cohen AL (2011). Projecting coral reef futures under global warming and ocean acidification. Science..

[7] Hoegh-Guldberg O, Poloczanska ES, Skirving W, Dove S (2017). Coral reef ecosystems under climate change and ocean acidification. Front Mar Sci..

[8] Wang L, Shantz AA, Payet JP, Sharpton TJ, Foster A, Burkepile DE, Vega Thurber R (2018). Corals and their microbiomes are differentially affected by exposure to elevated nutrients and a natural thermal anomaly. Front Mar Sci..

[9] Wooldridge SA (2009). Water quality and coral bleaching thresholds: formalising the linkage for the inshore reefs of the Great Barrier Reef. Australia. Mar Pollut Bull..

[10] Loya Y, Rinkevich B (1980). Effects of oil pollution on coral reef communities. Mar Ecol Prog Ser..

[11] White HK, Hsing P-Y, Cho W, Shank TM, Cordes EE, Quattrini AM, Nelson RK, Camilli R, Demopoulos AWJ, German CR, Brooks JM, Roberts HH, Shedd W, Reddy CM, Fisher CR (2012). Impact of the Deepwater Horizon oil spill on a deep-water coral community in the Gulf of Mexico. Proc Natl Acad Sci USA..

[12] Atlas RM, Hazen TC (2011). Oil biodegradation and bioremediation: a tale of the two worst spills in U.S. history. Environ Sci Technol..

[13] Escobar H (2019). Mystery oil spill threatens marine sanctuary in Brazil. Science..

[14] Peterson CH, Rice SD, Short JW, Esler D, Bodkin JL, Ballachey BE, Irons DB (2003). Long-term ecosystem response to the Exxon Valdez oil spill. Science..

[15] Villela HDM, Peixoto RS, Soriano AU, do Carmo FL (2019). Microbial bioremediation of oil contaminated seawater: a survey of patent deposits and the characterization of the top genera applied. Sci Total Environ.

[16] Rinkevich B, Loya Y (1979). The reproduction of the Red Sea coral *Stylophora pistillata*. I. Gonads and planulae. Mar Ecol Prog Ser..

[17] Branan N. Chemicals worse for corals than oil. Geotimes. 2007;52:8–9. http://www.geotimes.org/oct07/article.html?id=nn_corals.html.

[18] Haapkylä J, Ramade F, Salvat B. Oil pollution on coral reefs: A review of the state of knowledge and management needs. 2007;57(1/2):91–107. https://hal.archives-ouvertes.fr/hal-00172433/.

[19] Shafir S, Van Rijn J, Rinkevich B (2007). Short and Long Term Toxicity of Crude Oil and Oil Dispersants to Two Representative Coral Species. Environ Sci Technol..

[20] DeLeo DM, Ruiz-Ramos DV, Baums IB, Cordes EE (2016). Response of deep-water corals to oil and chemical dispersant exposure. Deep Sea Res Part II Top Stud Oceanogr..

[21] Peixoto R, Rosado PM, Leite DC, Rosado AS, Bourne DG (2017). Beneficial microorganisms for corals (BMC): proposed mechanisms for coral health and resilience. Front Microbiol..

[22] Rohwer F, Seguritan, Azam, Knowlton N (2002). Diversity and distribution of coral-associated bacteria. Mar Ecol Prog Ser.

[23] Rosenberg E, Koren O, Reshef L, Efrony R, Zilber-Rosenberg I (2007). The role of microorganisms in coral health, disease and evolution. Nat Rev Microbiol..

[24] Kimes NE, Van Nostrand JD, Weil E, Zhou J, Morris PJ (2010). Microbial functional structure of *Montastraea faveolata*, an important Caribbean reef-building coral, differs between healthy and yellow-band diseased colonies. Environ Microbiol..

[25] Raina J-B, Tapiolas D, Willis BL, Bourne DG (2009). Coral-associated bacteria and their role in the biogeochemical cycling of sulfur. Appl Environ Microbiol..

[26] Wegley L, Edwards R, Rodriguez-Brito B, Liu H, Rohwer F (2007). Metagenomic analysis of the microbial community associated with the coral *Porites astreoides*. Environ Microbiol..

[27] Ritchie KB. Bacterial symbionts of corals and *Symbiodinium*. In: Beneficial Microorganisms in Multicellular Life Forms. Springer; 2012. p. 139–150. 10.1007/978-3-642-21680-0_9.

[28] Dunlap WC, Shick JM (1998). Ultraviolet radiation-absorbing mycosporine-like amino acids in coral reef organisms: a biochemical and environmental perspective. J Phycol..

[29] Fine M, Loya Y (2002). Endolithic algae: an alternative source of photoassimilates during coral bleaching. Proc Biol Sci..

[30] Janouškovec J, Horák A, Barott KL, Rohwer FL, Keeling PJ (2012). Global analysis of plastid diversity reveals apicomplexan-related lineages in coral reefs. Curr Biol.

[31] Wilkins LGE, Leray M, O’Dea A, Yuen B, Peixoto RS, Pereira TJ (2019). Host-associated microbiomes drive structure and function of marine ecosystems. PLoS Biol..

[32] Santos H, Duarte GAS, Rachid CT da C, Chaloub RM, Calderon EN, Marangoni LF de B, et al. Impact of oil spills on coral reefs can be reduced by bioremediation using probiotic microbiota. Sci Rep. 2015;5:18268. 10.1038/srep18268, 1. 10.1038/srep18268 PMC4677405 26658023

[33] Rosado P, Leite DCA, Duarte GAS, Chaloub RM, Jospin G, Nunes da Rocha U (2019). Marine probiotics: increasing coral resistance to bleaching through microbiome manipulation. ISME J..

[34] Boonchan S, Britz ML, Stanley GA (2000). Degradation and mineralization of high-molecular-weight polycyclic aromatic hydrocarbons by defined fungal-bacterial cocultures. Appl Environ Microbiol..

[35] Lane DJ, Stackebrandt E, Goodfellow M (1991). 16S/23S rRNA sequencing. Nucleic acid techniques in bacterial systematics.

[36] White TJ, Bruns T, Lee S, Taylor J. Amplification and direct sequencing of fungal ribosomal RNA Genes for phylogenetics. 1990. PCR - Protocols and Applications - A Laboratory Manual p. 315–22.

[37] O’Donnell K (1993). Fusarium and its near relatives.

[38] Silva D, Duarte G, Villela HDM, Santos HF, Rosado PM, Rosado JG (2019). Adaptable mesocosm facility to study oil spill impacts on corals. Ecol Evol..

[39] CONAMA. RESOLUÇÃO no 472, de 27 de Novembro de 2015. 2015. http://www2.mma.gov.br/port/conama/legiabre.cfm?codlegi=718.

[40] United States Environmental Protection Agency. Method 3510C. Separatory funnel liquid-liquid extraction, Revision 3. 1996;:8. https://www.epa.gov/sites/production/files/2015-12/documents/3510c.pdf.

[41] United States Environmental Protection Agency. Method 8270D. Semivolatile organic compounds by gas chromatography/mass spectrometry (GC/MS), Revision 4. 2007;:62. https://www.epa.gov/sites/production/files/2015-07/documents/epa-8270d.pdf.

[42] Pinheiro J, Bates D, DebRoy S, Sarkar D, Heisterkamp S, Van Willigen B, et al. Package ‘nlme.’ Linear Nonlinear Mix Eff Model version. 2017;:1–3.

[43] Team RC (2017). R: A language and environment for statistical computing (Version 3.4. 2)[Computer software].

[44] Lenth R, Lenth MR (2018). Package ‘lsmeans’. Am Stat..

[45] Caporaso JG, Lauber CL, Walters WA, Berg-Lyons D, Lozupone CA, Turnbaugh PJ (2011). Global patterns of 16S rRNA diversity at a depth of millions of sequences per sample. Proc Natl Acad Sci USA.

[46] McCune B, Grace JB. Analysis of ecological communities. MjM Software Design, Gleneden Beach, Oregon. 2002. 10.1016/S0022-0981(03)00091-1.

[47] Dufrene M, Legendre P (1997). Species assemblages and indicator species: the need for a flexible asymmetrical approach. Ecol Monogr..

[48] Shaver EC, Burkepile DE, Silliman BR (2018). Local management actions can increase coral resilience to thermally-induced bleaching. Nat Ecol Evol..

[49] Peixoto R, Sweet M, Villela HDM, Cardoso PM, Thomas T, Voolstra CR, et al. Coral Probiotics: Premise, Promise, Prospects. Annu Rev Anim Biosci. 2021; 10.1146/annurev-animal-090120-115444. 10.1146/annurev-animal-090120-115444 33321044

[50] United Nations. A-68-970 Report of the Open Working Group of the General Assembly on Sustainable Development Goals. 2014. https://digitallibrary.un.org/record/784147?ln=en.

[51] National Academies of Sciences and Medicine E (2019). A research review of interventions to increase the persistence and resilience of coral reefs.

[52] do Carmo FL, dos Santos HF, Martins EF, van Elsas JD, Rosado AS, Peixoto RS (2011). Bacterial structure and characterization of plant growth promoting and oil degrading bacteria from the rhizospheres of mangrove plants. J Microbiol.

[53] Cury JC, Jurelevicius DA, Villela HDM, Jesus HE, Peixoto RS, Schaefer CEGR, Bícego MC, Seldin L, Rosado AS (2015). Microbial diversity and hydrocarbon depletion in low and high diesel-polluted soil samples from Keller Peninsula, South Shetland Islands. Antarct Sci..

[54] Schrezenmeir J, de Vrese M (2001). Probiotics, prebiotics, and synbiotics—approaching a definition. Am J Clin Nutr.

[55] Reshef L, Koren O, Loya Y, Zilber-Rosenberg I, Rosenberg E (2006). The coral probiotic hypothesis. Environ Microbiol..

[56] Corbo MR, Campaniello D, Speranza B, Altieri C, Sinigaglia M, Bevilacqua A (2018). Neutralisation of toxins by probiotics during the transit into the gut: challenges and perspectives. Int J Food Sci Technol..

[57] Kshatri J, Rao CV, Settaluri VS (2018). Neutralization of toxins in aqua culture using probiotics. Int J Pharm Sci Res.

[58] Kumari S, Regar RK, Manickam N (2018). Improved polycyclic aromatic hydrocarbon degradation in a crude oil by individual and a consortium of bacteria. Bioresour Technol..

[59] Bretherton L, Kamalanathan M, Genzer J, Hillhouse J, Setta S, Liang Y, Brown CM, Xu C, Sweet J, Passow U, Finkel ZV, Irwin AJ, Santschi PH, Quigg A (2019). Response of natural phytoplankton communities exposed to crude oil and chemical dispersants during a mesocosm experiment. Aquat Toxicol..

[60] Wise J, Wise JP (2011). A review of the toxicity of chemical dispersants. Rev Environ Health..

[61] Studivan MS, Hatch WI, Mitchelmore CL (2015). Responses of the soft coral *Xenia elongata* following acute exposure to a chemical dispersant. Springerplus..

[62] Negri AP, Hoogenboom MO (2011). Water contamination reduces the tolerance of coral larvae to thermal stress. PLoS One..

[63] Yakimov MM, Timmis KN, Golyshin PN (2007). Obligate oil-degrading marine bacteria. Curr Opin Biotechnol..

[64] Gallego S, Vila J, Tauler M, Nieto JM, Breugelmans P, Springael D, Grifoll M (2014). Community structure and PAH ring-hydroxylating dioxygenase genes of a marine pyrene-degrading microbial consortium. Biodegradation..

[65] Hara A, Syutsubo K, Harayama S (2003). Alcanivorax which prevails in oil-contaminated seawater exhibits broad substrate specificity for alkane degradation. Environ Microbiol.

[66] Li C, Lai Q, Li G, Liu Y, Sun F, Shao Z (2014). Multilocus sequence analysis for the assessment of phylogenetic diversity and biogeography in hyphomonas bacteria from diverse marine environments. PLoS One..

[67] Mulkins-Phillips GJ, Stewart JE (1974). Effect of four dispersants on biodegradation and growth of bacteria on crude oil. Appl Microbiol..

[68] Gillan DC, Danis B, Pernet P, Joly G, Dubois P (2005). Structure of sediment-associated microbial communities along a heavy-metal contamination gradient in the marine environment. Appl Environ Microbiol..

[69] Apprill A, Hughen K, Mincer T (2013). Major similarities in the bacterial communities associated with lesioned and healthy Fungiidae corals. Environ Microbiol..

[70] Geffen Y, Ron EZ, Rosenberg E (2009). Regulation of release of antibacterials from stressed scleractinian corals. FEMS Microbiol Lett..

[71] Cooney RP, Pantos O, Tissier MD, Barer MR, O’Donnell AG, Bythell JC. Characterization of the bacterial consortium associated with black band disease in coral using molecular microbiological techniques. Environ Microbiol. 2002;4:401–13. doi:10.1046/j.1462-2920.2002.00308.x. 10.1046/j.1462-2920.2002.00308.x 12123476

[72] Miller AW, Richardson LL (2011). A meta-analysis of 16S rRNA gene clone libraries from the polymicrobial black band disease of corals. FEMS Microbiol Ecol..

[73] Pantos O, Bythell JC (2006). Bacterial community structure associated with white band disease in the elkhorn coral *Acropora palmata* determined using culture-independent 16S rRNA techniques. Dis Aquat Organ..

[74] Pantos O, Cooney RP, Le Tissier MDA, Barer MR, O’Donnell AG, Bythell JC (2003). The bacterial ecology of a plague-like disease affecting the Caribbean coral *Montastrea annularis*. Environ Microbiol..

[75] Séré MG, Tortosa P, Chabanet P, Turquet J, Quod J-P, Schleyer MH (2013). Bacterial communities associated with *Porites* white patch syndrome (PWPS) on three Western Indian Ocean (WIO) coral reefs. PLoS One..

[76] Viehman S, Mills DK, Meichel GW, Richardson LL (2006). Culture and identification of *Desulfovibrio* spp. from corals infected by black band disease on Dominican and Florida Keys reefs. Dis Aquat Organ..

[77] McFarlin KM, Perkins MJ, Field JA, Leigh MB (2018). Biodegradation of Crude Oil and Coprexit 9500 in Artic Seawater. Front. Microbiol..

[78] Gignoux-Wolfsohn SA, Vollmer SV (2015). Identification of candidate coral pathogens on white band disease-infected staghorn coral. PLoS One..

[79] Sunagawa S, DeSantis TZ, Piceno YM, Brodie EL, DeSalvo MK, Voolstra CR (2009). Bacterial diversity and White Plague Disease-associated community changes in the Caribbean coral *Montastraea faveolata*. ISME J..

[80] Lee STM, Davy SK, Tang S-L, Fan T-Y, Kench PS (2015). Successive shifts in the microbial community of the surface mucus layer and tissues of the coral *Acropora muricata* under thermal stress. FEMS Microbiol Ecol.

[81] McDevitt-Irwin JM, Baum JK, Garren M, Vega Thurber RL (2017). Responses of coral-associated bacterial communities to local and global stressors. Front Mar Sci..

[82] Salter I, Zubkov MV, Warwick PE, Burkill PH (2009). Marine bacterioplankton can increase evaporation and gas transfer by metabolizing insoluble surfactants from the air-seawater interface. FEMS Microbiol Lett..

[83] Le Roux F, Wegner KM, Baker-Austin C, Vezzulli L, Osorio CR, Amaro C (2015). The emergence of *Vibrio* pathogens in Europe: ecology, evolution, and pathogenesis (Paris, 11-12th March 2015). Front Microbiol..

[84] Boyd EF, Moyer KE, Shi L, Waldor MK (2000). Infectious CTXPhi and the vibrio pathogenicity island prophage in *Vibrio mimicus*: evidence for recent horizontal transfer between *V. mimicus* and *V. cholerae*. Infect Immun..

[85] Jermyn WS, Boyd EF (2002). Characterization of a novel *Vibrio* pathogenicity island (VPI-2) encoding neuraminidase (nanH) among toxigenic *Vibrio cholerae* isolates. Microbiology..

[86] Ben-Haim Y, Zicherman-Keren M, Rosenberg E (2003). Temperature-regulated bleaching and Lysis of the coral *Pocillopora damicornis* by the novel pathogen *Vibrio coralliilyticus*. Appl Environ Microbiol..

[87] Banin E, Ben-Haim Y, Israely T, Loya Y, Rosenberg E. Effect of the environment on the bacterial bleaching of corals. In: Belkin S, editor. Environmental Challenges. Dordrecht: Springer; 2000. p. 337–352. 10.1007/978-94-011-4369-1_27.

[88] Ben-Haim Y, Rosenberg E (2002). A novel *Vibrio* sp. pathogen of the coral *Pocillopora damicornis*. Mar Biol..

[89] Rosenberg E, Ben-Haim Y (2002). Microbial diseases of corals and global warming. Environ Microbiol..

[90] Hamdan LJ, Fulmer PA. Effects of COREXIT® EC9500A on bacteria from a beach oiled by the Deepwater Horizon spill. Aquat Microb Ecol, DOI. 2011;63:101–9 10.3354/ame01482.

[91] Chen Y-H, Kuo J, Sung P-J, Chang Y-C, Lu M-C, Wong T-Y, Liu JK, Weng CF, Twan WH, Kuo FW (2012). Isolation of marine bacteria with antimicrobial activities from cultured and field-collected soft corals. World J Microbiol Biotechnol..

[92] Rodrigues GN, Lago-Leston A, Costa R, Keller-Costa T. Draft genome sequence of *Labrenzia* sp. strain EL143, a coral-associated alphaproteobacterium with versatile symbiotic living capability and strong halogen degradation potential. Genome Announc. 2018;6. DOI: 10.1128/genomeA.00132-18. 10.1128/genomeA.00132-18 PMC5843722 29519836

[93] Rubio-Portillo E, Kersting DK, Linares C, Ramos-Esplá AA, Antón J (2018). Biogeographic differences in the microbiome and pathobiome of the coral *Cladocora caespitosa* in the Western Mediterranean Sea. Front Microbiol..

[94] Thomas S, Burdett H, Temperton B, Wick R, Snelling D, McGrath JW (2010). Evidence for phosphonate usage in the coral holobiont. ISME J..

[95] Yu Z, Lai Q, Li G, Shao Z (2013). *Parvularcula dongshanensis* sp. nov., isolated from soft coral. Int J Syst Evol Microbiol.

[96] Ziegler M, Roik A, Porter A, Zubier K, Mudarris MS, Ormond R, Voolstra CR (2016). Coral microbial community dynamics in response to anthropogenic impacts near a major city in the central Red Sea. Mar Pollut Bull..

[97] Bayer T, Neave MJ, Alsheikh-Hussain A, Aranda M, Yum LK, Mincer T, Hughen K, Apprill A, Voolstra CR (2013). The Microbiome of the Red Sea Coral *Stylophora pistillata* is dominated by tissue-associated *Endozoicomonas* bacteria. Appl Environ Microbiol..

[98] Neave MJ, Michell CT, Apprill A, Voolstra CR (2017). *Endozoicomonas* genomes reveal functional adaptation and plasticity in bacterial strains symbiotically associated with diverse marine hosts. Sci Rep..

[99] Speck MD, Donachie SP. Widespread Oceanospirillaceae Bacteria in *Porites* spp. J Mar Sci. 2012;746720. 10.1155/2012/746720.

[100] Neave MJ, Rachmawati R, Xun L, Michell CT, Bourne DG, Apprill A, Voolstra CR (2017). Differential specificity between closely related corals and abundant *Endozoicomonas* endosymbionts across global scales. ISME J..

[101] Röthig T, Yum LK, Kremb SG, Roik A, Voolstra CR (2017). Microbial community composition of deep-sea corals from the Red Sea provides insight into functional adaption to a unique environment. Sci Rep..

[102] Simister RL, Antzis EW, White HK (2016). Examining the diversity of microbes in a deep-sea coral community impacted by the Deepwater Horizon oil spill. Deep Sea Res Part II Top Stud Oceanogr..

[103] Al-Dahash LM, Mahmoud HM (2013). Harboring oil-degrading bacteria: a potential mechanism of adaptation and survival in corals inhabiting oil-contaminated reefs. Mar Pollut Bull..

[104] Sauret C, Séverin T, Vétion G, Guigue C, Goutx M, Pujo-Pay M, Conan P, Fagervold SK, Ghiglione JF (2014). ‘Rare biosphere’ bacteria as key phenanthrene degraders in coastal seawaters. Environ Pollut..

[105] Pascoal F, Magalhães C, Costa R (2020). The link between the ecology of the prokaryotic rare biosphere and its biotechnological potential. Front Microbiol..

[106] Dahiya DK, Puniya M, Shandilya UK, Dhewa T, Kumar N, Kumar S (2017). Gut microbiota modulation and its relationship with obesity using prebiotic fibers and probiotics: a review. Front Microbiol..

[107] Shin D, Chang SY, Bogere P, Won K, Choi J-Y, Choi Y-J, Lee HK, Hur J, Park BY, Kim Y, Heo J (2019). Beneficial roles of probiotics on the modulation of gut microbiota and immune response in pigs. PLoS One..

[108] Mies M, Francini-Filho RB, Zilberberg C, Garrido AG, Longo GO, Laurentino E (2020). South Atlantic coral reefs are major global warming refugia and less susceptible to bleaching. Front Mar Sci..

[109] Duarte GAS, Villela HDM, Deocleciano M, Silva D, Barno A, Cardoso PM, Vilela CLS, Rosado P, Messias CSMA, Chacon MA, Santoro EP, Olmedo DB, Szpilman M, Rocha LA, Sweet M, Peixoto RS (2020). Heat Waves Are a Major Threat to Turbid Coral Reefs in Brazil. Front Mar Sci..

