# Correction: Multi-domain probiotic consortium as an alternative to chemical remediation of oil spills at coral reefs and adjacent sites

**DOI:** 10.1186/s40168-024-01845-6

**Published:** 2024-06-10

**Authors:** Denise P. Silva, Helena D. M. Villela, Henrique F. Santos, Gustavo A. S. Duarte, José Roberto Ribeiro, Angela M. Ghizelini, Caren L. S. Vilela, Phillipe M. Rosado, Carolline S. Fazolato, Erika P. Santoro, Flavia L. Carmo, Dalton S. Ximenes, Adriana U. Soriano, Caio T. C. C. Rachid, Rebecca L. Vega Thurber, Raquel S. Peixoto

**Affiliations:** 1 LEMM, Laboratory of Molecular Microbial Ecology, Institute of Microbiology Paulo de Góes, Federal University of Rio de Janeiro (UFRJ), Rio de Janeiro, Brazil; 2 Department of Marine Biology, Fluminense Federal University (UFF), Niterói, Brazil; 3 Processes Laboratory, Leopoldo Américo Miguez de Mello Research Center (CENPES), Petrobras, Rio de Janeiro, Brazil; 4 Environmental Treatments, Wastes and Water Resources, Leopoldo Américo Miguez de Mello Research Center (CENPES), Petrobras, Rio de Janeiro, Brazil; 5 LABEM, Paulo de Góes Institute of Microbiology, Federal University of Rio de Janeiro (UFRJ), Rio de Janeiro, Brazil; 6 Department of Microbiology, Oregon State University, Nash Hall 226, OSU, Corvallis, OR 97331 USA; 7 Division of Biological and Environmental Science and Engineering (BESE), Red Sea Research Center, King Abdullah University of Science and Technology (KAUST), Thuwal, 23955-6900 Kingdom of Saudi Arabia

**Correction** **:** **Microbiome 9, 118 (2021)**

**https://doi.org/10.1186/s40168-021-01041-w**

Following publication of the original article [1], the authors stated that in the "**Availability of data and materials**" section, they included the provisional NCBI code (SUB6046921) for DNA sequences. However, this code currently is not available. To ensure data accessibility, they would like to include the following sentence for clarity:

**"The 16S rRNA gene dataset is available under BioProject ID PRJNA556854 (** https://www.ncbi.nlm.nih.gov/bioproject/PRJNA556854 **)"**

The original article has been updated.
