# Supplementary material for: Multi-domain probiotic consortium as an alternative to chemical remediation of oil spills at coral reefs and adjacent sites
Source: Microbiome. 2021 May 21;9:118. doi: 10.1186/s40168-021-01041-w (PMC8138999; doi:10.1186/s40168-021-01041-w)
Supplement: Supplementary file 2 — Additional file 1. Statistical analyses of all parameters, performed using R software. [file 40168_2021_1041_MOESM2_ESM.pdf]

Repeated Measures Analysis

06 May 2019

- Read in data and summarize
- Repeated measures ANOVA
    - Fv/Fm
    - Salinity
    - pH
    - DOC
    - Ammonium
    - Nitrate
    - Phosphate
    - PAH

Read in data and summarize

```
options(scipen=999)
dat <- read.csv('./Dados - 2 rodada - all treatments.csv')
dat2 <- read.csv('./Fv_Fm - 2 rodada - all treatments - 20190424.csv')
# Remove observations
dat[dat$Treatments == 'oWSF+pBMC-BC' & dat$ID == 'P', 4:length(dat)] <- NA
dat2[dat2$Treatments == 'oWSF+pBMC-BC' & dat2$ID == 'P', ] <- NA

#Make Time a categorical variable
#Fv_Fm was measured more often than the other parameters
dat$Time <- as.factor(dat$Time)
dat2$Time <- as.factor(dat2$Days)
summary(dat)
```

```
##                         Treatments       ID       Time      Salinity
##  Control                     :12   A      : 3   0:32   Min.   :35.70
##  Corexit 9500                :12   AA     : 3   1:32   1st Qu.:36.10
##  oWSF                        :12   B      : 3   2:32   Median :36.40
##  oWSF+Corexit 9500           :12   BB     : 3          Mean   :36.56
##  oWSF+pBMC-BC                :12   C      : 3          3rd Qu.:36.70
##  oWSF+pBMC-BC+Corexit 9500:12   CC     : 3          Max.   :38.10
##  (Other)                     :24   (Other):78          NA's   :3
##        pH           Ammonium         Nitrate          Phosphate
##  Min.   :7.800   Min.   : 11.10   Min.   :  0.00   Min.   :  0.00
##  1st Qu.:8.040   1st Qu.: 28.10   1st Qu.:  0.00   1st Qu.:  9.00
##  Median :8.220   Median : 40.50   Median :  0.00   Median : 26.31
##  Mean   :8.257   Mean   : 96.51   Mean   : 33.09   Mean   : 50.03
##  3rd Qu.:8.490   3rd Qu.: 93.40   3rd Qu.: 41.10   3rd Qu.: 58.50
##  Max.   :8.800   Max.   :532.20   Max.   :358.60   Max.   :391.00
##  NA's   :3       NA's   :3        NA's   :3        NA's   :3
##       DOC                TPH                PAH
##  Min.   :   0.00   Min.   :    0.00   Min.   :   0.000
##  1st Qu.:  35.21   1st Qu.:    0.00   1st Qu.:   0.148
##  Median :  77.35   Median :   50.17   Median :   1.843
##  Mean   : 134.43   Mean   : 3278.31   Mean   : 693.816
##  3rd Qu.: 182.00   3rd Qu.: 2957.00   3rd Qu.:  53.666
##  Max.   :1035.00   Max.   :49110.00   Max.   :8045.189
##  NA's   :3         NA's   :3          NA's   :3
```

```
summary(dat2)
```

```
##                         Treatments       ID           Days
##  Control                     :20   A      :  5   Min.   : 1.0
##  Corexit 9500                :20   AA     :  5   1st Qu.: 4.0
##  oWSF                        :20   B      :  5   Median : 7.0
##  oWSF+Corexit 9500           :20   BB     :  5   Mean   : 6.8
##  oWSF+pBMC-BC+Corexit 9500:20   C      :  5   3rd Qu.: 9.0
##  (Other)                     :55   (Other):130   Max.   :13.0
##  NA's                        : 1   NA's   :  1   NA's   :1
##      Fv_Fm           Time
##  Min.   :0.0000   1   :31
##  1st Qu.:0.0400   4   :31
##  Median :0.5370   7   :31
##  Mean   :0.3643   9   :31
##  3rd Qu.:0.6072   13  :31
##  Max.   :0.6530   NA's: 1
##  NA's   :2
```

Repeated measures ANOVA

Fv/Fm

Fv/Fm was measured more often than other physical/chemical parameters and the analysis refers to days rather than times

Treatments with dispersant (Corexit 9500) by day 4 and onward had significantly lower Fv/Fm ratios when compared with day 1. In addition, dispersant treatments were different from all other treatments for the remainder of the experiment. Fv/Fm in the oil treatment was different on day 13 than at the beginning of the experiment (day 1).

```
library(nlme)
library(emmeans)
library(DT);library(knitr)

#Create model  with Treatment, Time, Treatment and Time interaction
#Include ID as a random effect
model <- lme(Fv_Fm ~ Time*Treatments, random = ~1|ID, data=dat2, na.action = na.omit)
anova(model)
```

```
##                  numDF denDF   F-value p-value
## (Intercept)          1    91 2299.6102  <.0001
## Time                 4    91  116.9652  <.0001
## Treatments           7    23  119.9930  <.0001
## Time:Treatments     28    91   13.4514  <.0001
```

```
#summary(model)

marginal <- emmeans(model, pairwise ~ Time * Treatments)
plot(marginal, comparisons=T)
```

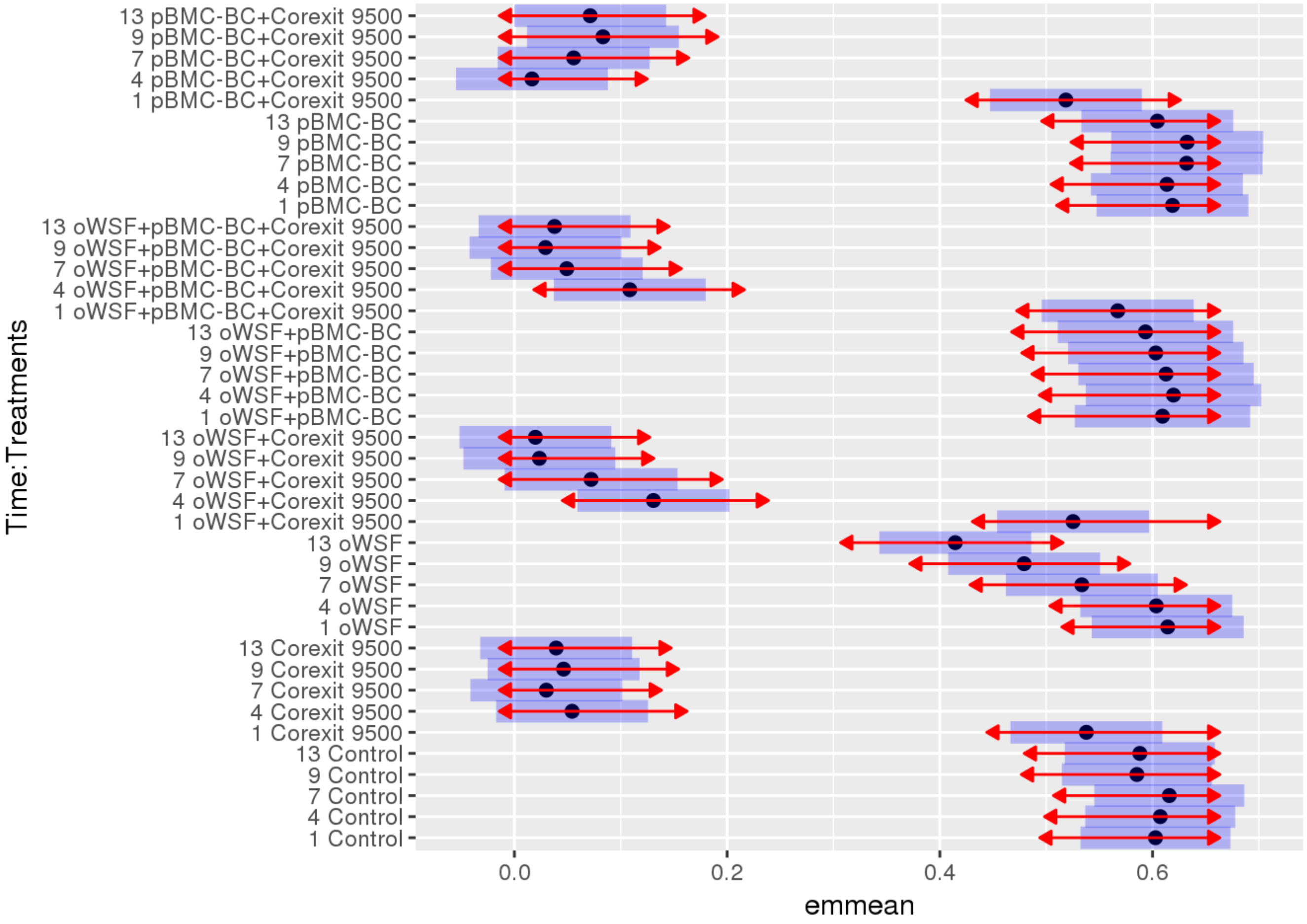


```
lsmip(model, Treatments~Time)
```

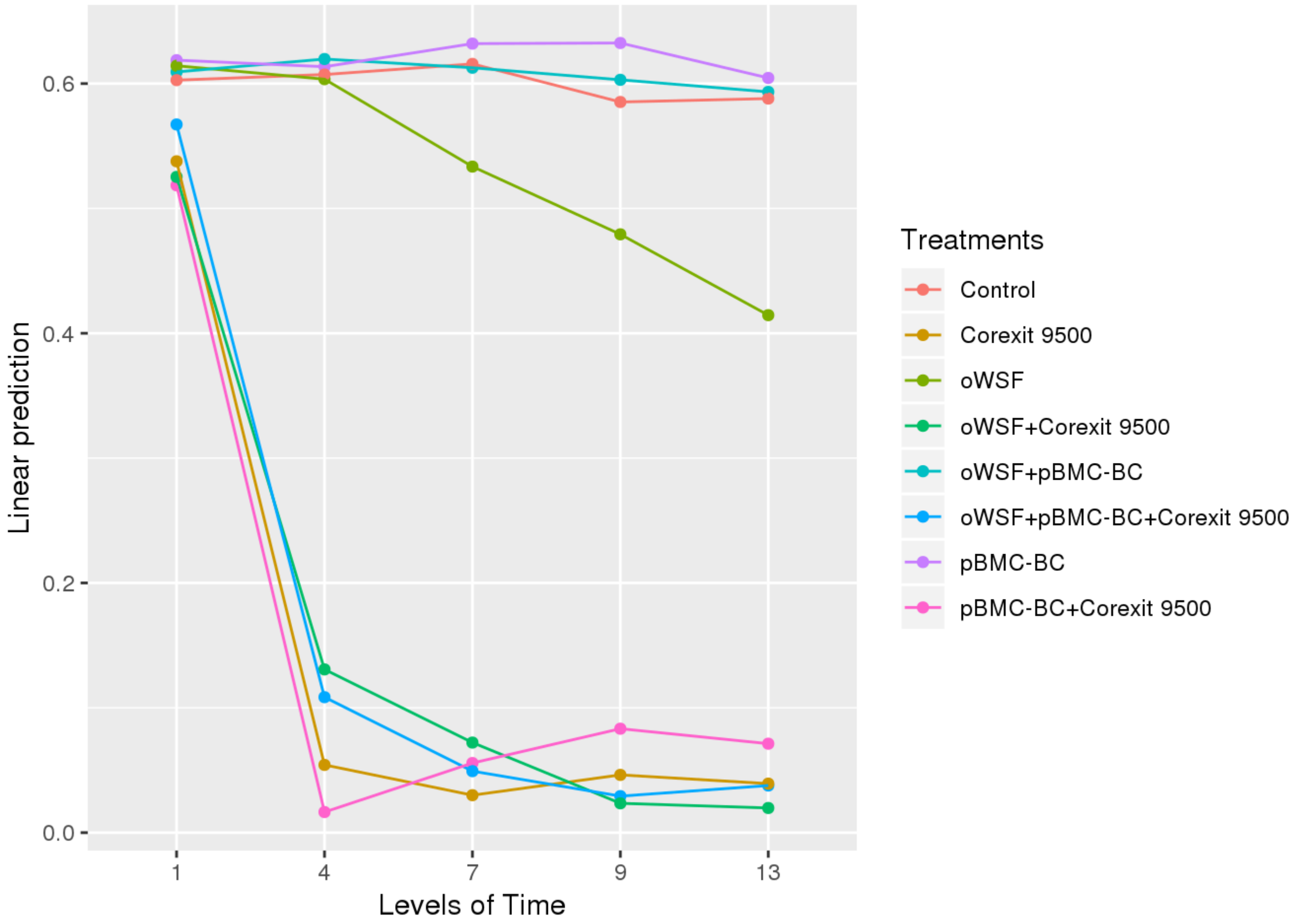


```
kable(CLD(marginal$emmeans), row.names = F)
```

Time	Treatments	emmean	SE	df	lower.CL	upper.CL	.group
4	pBMC-BC+Corexit 9500	0.0165000	0.0345117	23	-0.0548930	0.0878930	1
13	oWSF+Corexit 9500	0.0197500	0.0345117	23	-0.0516430	0.0911430	1
9	oWSF+Corexit 9500	0.0235000	0.0345117	23	-0.0478930	0.0948930	1
9	oWSF+pBMC-BC+Corexit 9500	0.0292500	0.0345117	23	-0.0421430	0.1006430	1
7	Corexit 9500	0.0300000	0.0345117	23	-0.0413930	0.1013930	1
13	oWSF+pBMC-BC+Corexit 9500	0.0377500	0.0345117	23	-0.0336430	0.1091430	1
13	Corexit 9500	0.0392500	0.0345117	23	-0.0321430	0.1106430	1
9	Corexit 9500	0.0462500	0.0345117	23	-0.0251430	0.1176430	1
7	oWSF+pBMC-BC+Corexit 9500	0.0492500	0.0345117	23	-0.0221430	0.1206430	1
4	Corexit 9500	0.0542500	0.0345117	23	-0.0171430	0.1256430	1
7	pBMC-BC+Corexit 9500	0.0557500	0.0345117	23	-0.0156430	0.1271430	1
13	pBMC-BC+Corexit 9500	0.0712500	0.0345117	23	-0.0001430	0.1426430	1
7	oWSF+Corexit 9500	0.0721437	0.0392823	23	-0.0091179	0.1534053	1
9	pBMC-BC+Corexit 9500	0.0832500	0.0345117	23	0.0118570	0.1546430	1
4	oWSF+pBMC-BC+Corexit 9500	0.1085000	0.0345117	23	0.0371070	0.1798930	1

Time	Treatments	emmean	SE	df	lower.CL	upper.CL	.group
4	oWSF+Corexit 9500	0.1307500	0.0345117	23	0.0593570	0.2021430	1
13	oWSF	0.4145000	0.0345117	23	0.3431070	0.4858930	2
9	oWSF	0.4792500	0.0345117	23	0.4078570	0.5506430	23
1	pBMC-BC+Corexit 9500	0.5185000	0.0345117	23	0.4471070	0.5898930	23
1	oWSF+Corexit 9500	0.5252500	0.0345117	23	0.4538570	0.5966430	23
7	oWSF	0.5335000	0.0345117	23	0.4621070	0.6048930	23
1	Corexit 9500	0.5377500	0.0345117	23	0.4663570	0.6091430	23
1	oWSF+pBMC-BC+Corexit 9500	0.5672500	0.0345117	23	0.4958570	0.6386430	23
9	Control	0.5852500	0.0345117	30	0.5147676	0.6557324	23
13	Control	0.5880000	0.0345117	30	0.5175176	0.6584824	23
13	oWSF+pBMC-BC	0.5933333	0.0398507	23	0.5108959	0.6757708	23
1	Control	0.6027500	0.0345117	30	0.5322676	0.6732324	23
9	oWSF+pBMC-BC	0.6030000	0.0398507	23	0.5205625	0.6854375	23
4	oWSF	0.6035000	0.0345117	23	0.5321070	0.6748930	3
13	pBMC-BC	0.6045000	0.0345117	23	0.5331070	0.6758930	23
4	Control	0.6072500	0.0345117	30	0.5367676	0.6777324	23
1	oWSF+pBMC-BC	0.6093333	0.0398507	23	0.5268959	0.6917708	23
7	oWSF+pBMC-BC	0.6126667	0.0398507	23	0.5302292	0.6951041	23
4	pBMC-BC	0.6135000	0.0345117	23	0.5421070	0.6848930	23
1	oWSF	0.6142500	0.0345117	23	0.5428570	0.6856430	3
7	Control	0.6157500	0.0345117	30	0.5452676	0.6862324	23
1	pBMC-BC	0.6187500	0.0345117	23	0.5473570	0.6901430	23
4	oWSF+pBMC-BC	0.6196667	0.0398507	23	0.5372292	0.7021041	23
7	pBMC-BC	0.6320000	0.0345117	23	0.5606070	0.7033930	3
9	pBMC-BC	0.6325000	0.0345117	23	0.5611070	0.7038930	3

```
#DT::datatable(data.frame(marginal$contrasts), options = list(pageLength = 20))
```

Salinity

The interaction term was not significant. Salinity increased over time and was higher at T2 than on T0 or T1. Among treatments, post hoc analysis showed that oWSF+pBMC-BC had significantly lower salinity than pBMC-BC+Corexit 9500.

```
#Create model  with Treatment, Time, Treatment and Time interaction
#Include ID as a random effect
model <- lme(Salinity ~ Time*Treatments, random = ~1|ID, data=dat, na.action = na.omit)
anova(model)
```

```
##                 numDF denDF  F-value p-value
## (Intercept)         1    46 684499.1  <.0001
## Time                2    46     78.9  <.0001
## Treatments          7    23      2.8  0.0300
## Time:Treatments    14    46      1.6  0.1144
```

```
#summary(model)

Time <- emmeans(model, ~ Time)
plot(Time, comparisons = T)
```

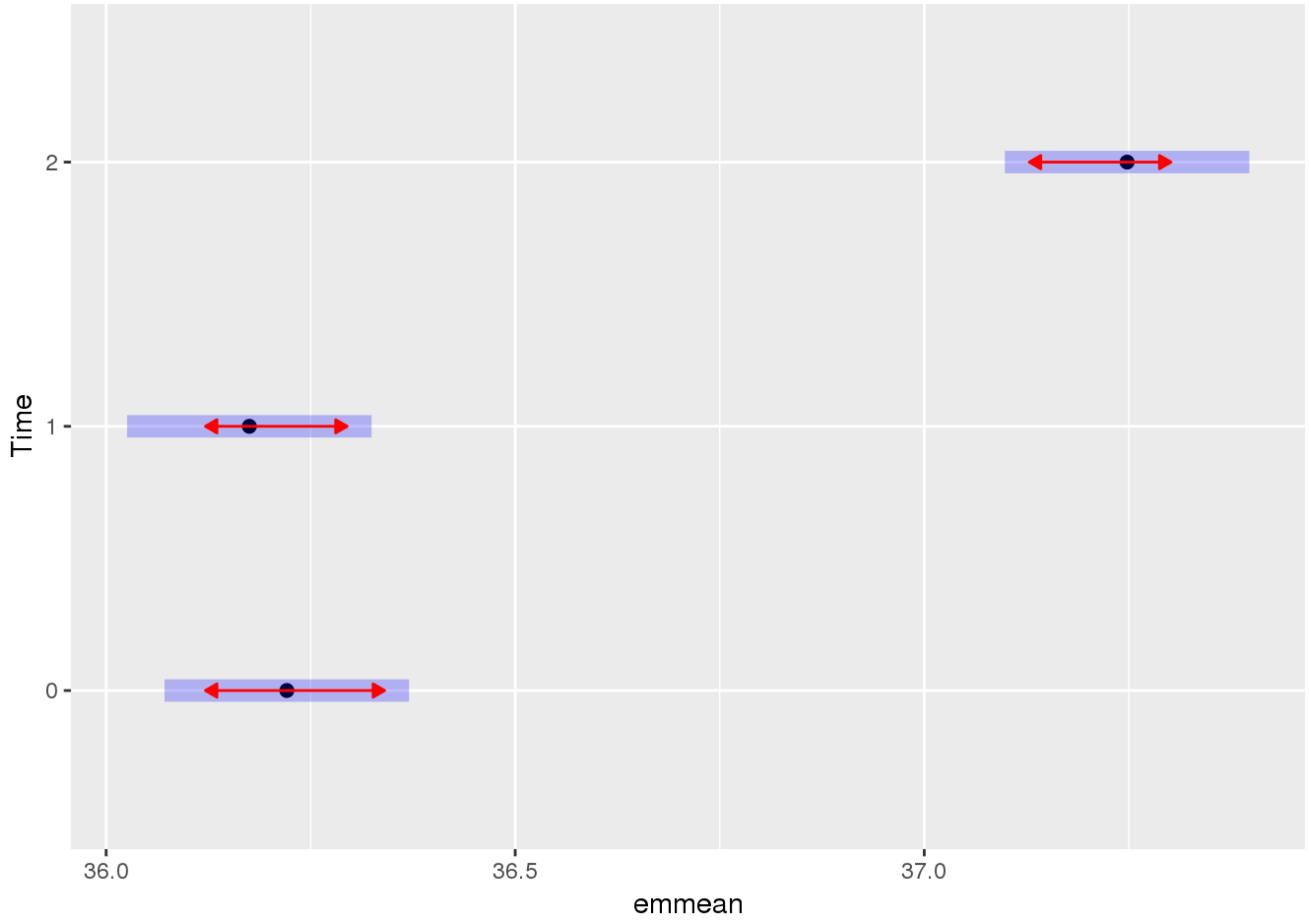


```
kable(CLD(Time), row.names = F)
```

Time	emmean	SE	df	lower.CL	upper.CL	.group
1	36.17500	0.0722287	23	36.02558	36.32442	1
0	36.22083	0.0722287	23	36.07142	36.37025	1

Time	emmean	SE	df	lower.CL	upper.CL	.group
2	37.24792	0.0722287	23	37.09850	37.39733	2

```
Treatments <- emmeans(model, ~ Treatments)
plot(Treatments, comparisons = T)
```

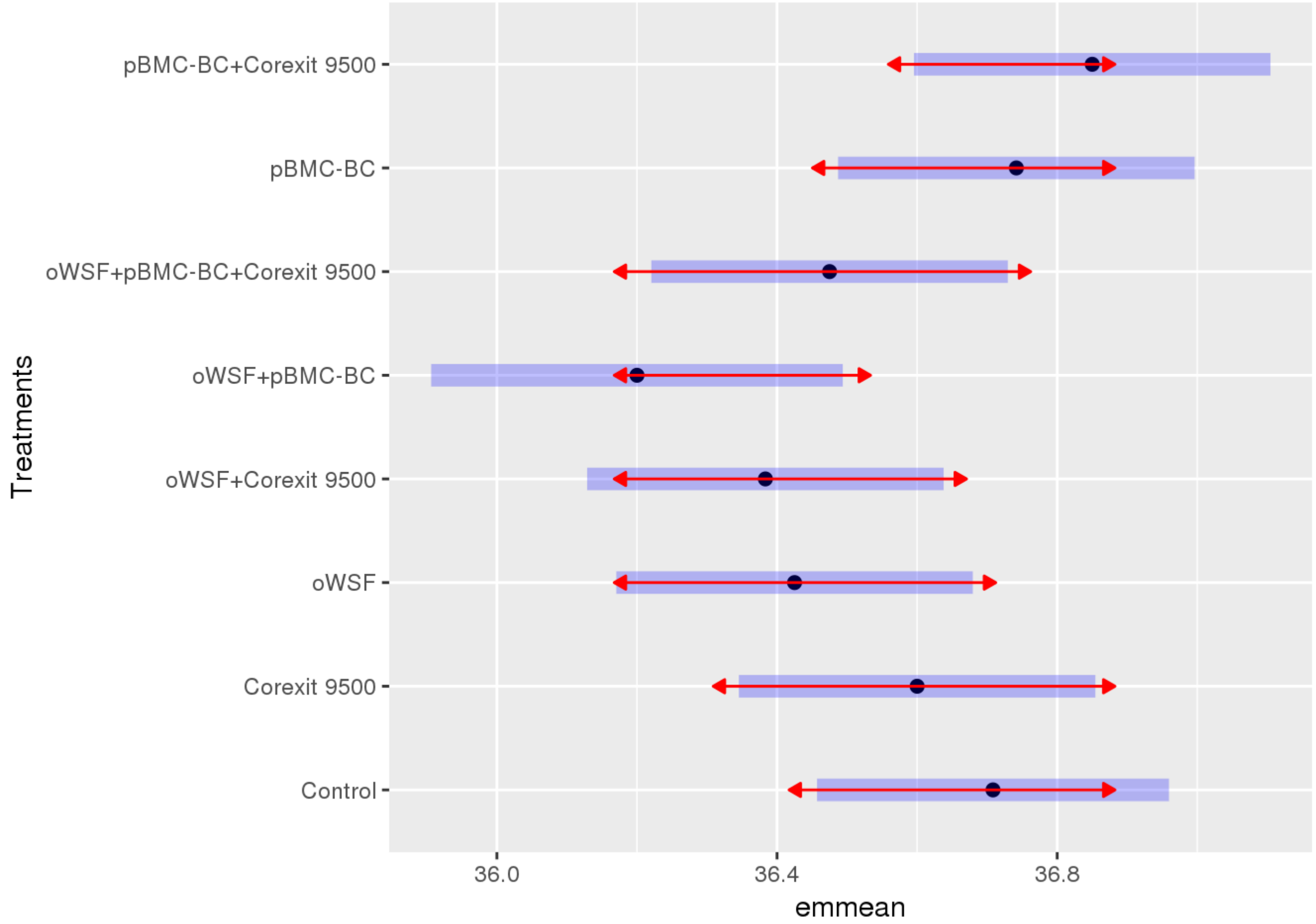


```
kable(CLD(Treatments), row.names = F)
```

Treatments	emmean	SE	df	lower.CL	upper.CL	.group
oWSF+pBMC-BC	36.20000	0.1420462	23	35.90615	36.49385	1
oWSF+Corexit 9500	36.38333	0.1230157	23	36.12886	36.63781	12
oWSF	36.42500	0.1230157	23	36.17052	36.67948	12
oWSF+pBMC-BC+Corexit 9500	36.47500	0.1230157	23	36.22052	36.72948	12
Corexit 9500	36.60000	0.1230157	23	36.34552	36.85448	12
Control	36.70833	0.1230157	30	36.45710	36.95956	12
pBMC-BC	36.74167	0.1230157	23	36.48719	36.99614	12
pBMC-BC+Corexit 9500	36.85000	0.1230157	23	36.59552	37.10448	2

```
lsmip(model, Treatments~Time)
```

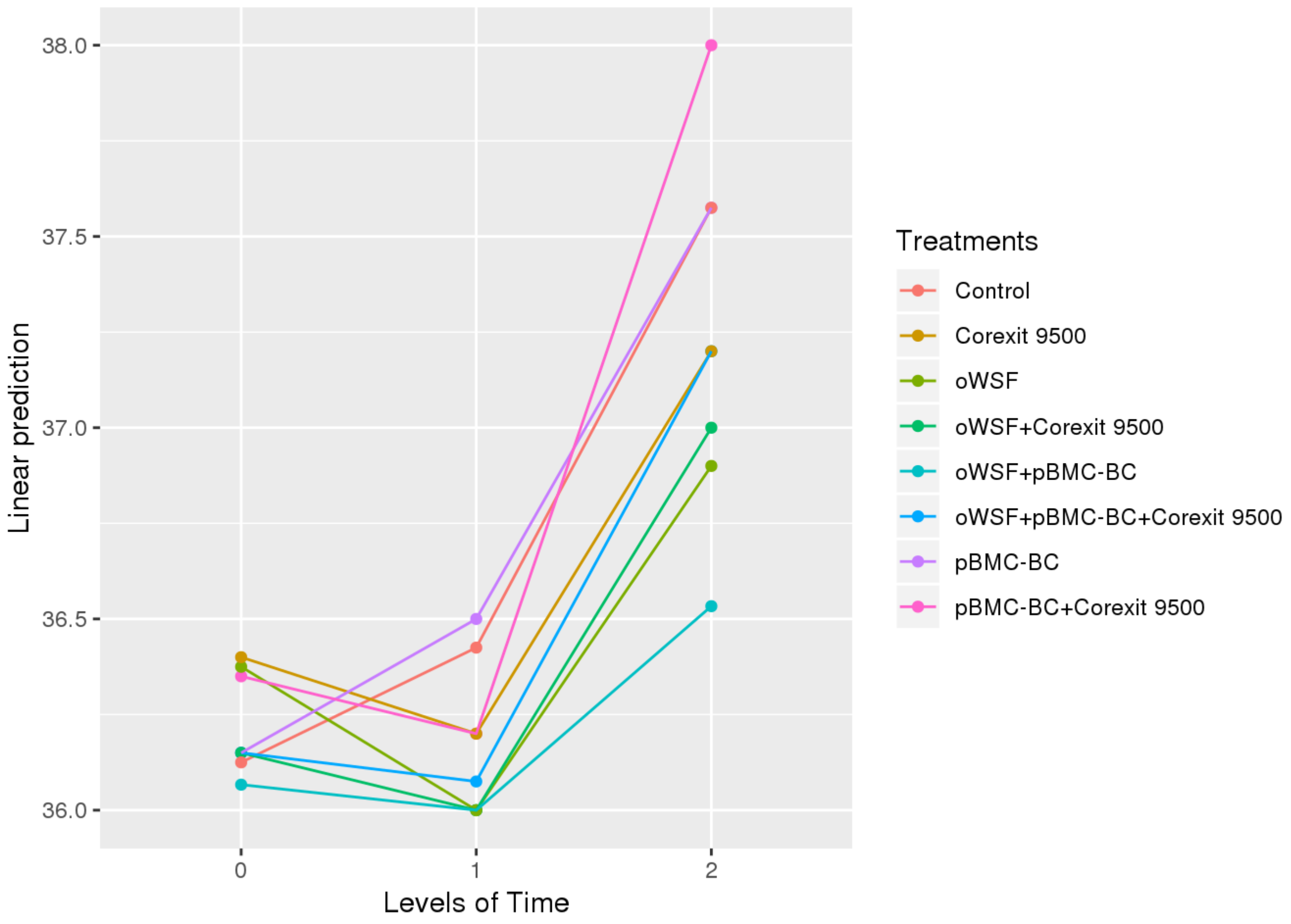

pH

The ANOVA interaction term was not significant. However, Treatments was significant. Post hoc analysis showed that oWSF+Corexit 9500, Corexit 9500, and oWSF+pBMC-BC+Corexit 9500 had significantly lower pH values than Control, oWSF+pBMC-BC, and pBMC-BC.

```
#Create model with Treatment, Time, Treatment and Time interaction
#Include ID as a random effect
model <- lme(pH ~ Time*Treatments, random = ~1|ID, data=dat, na.action = na.omit)
anova(model)
```

```
##                  numDF denDF   F-value p-value
## (Intercept)          1    46 280773.82  <.0001
## Time                 2    46      2.03  0.1426
## Treatments           7    23     32.02  <.0001
## Time:Treatments     14    46      0.58  0.8631
```

```
#summary(model)

marginal <- emmeans(model, ~ Treatments)
plot(marginal, comparisons=T)
```

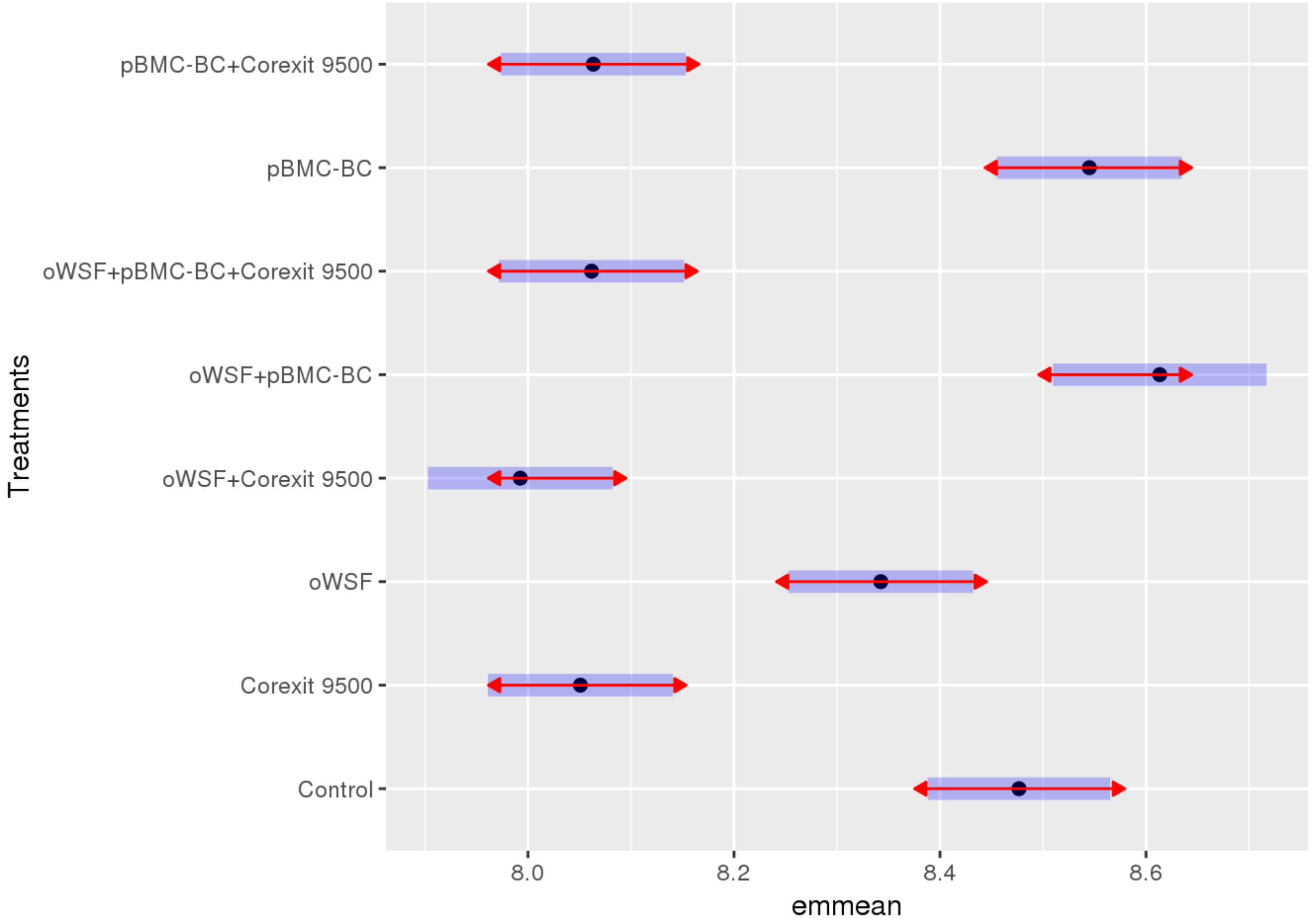


```
lsmip(model, Treatments~Time)
```

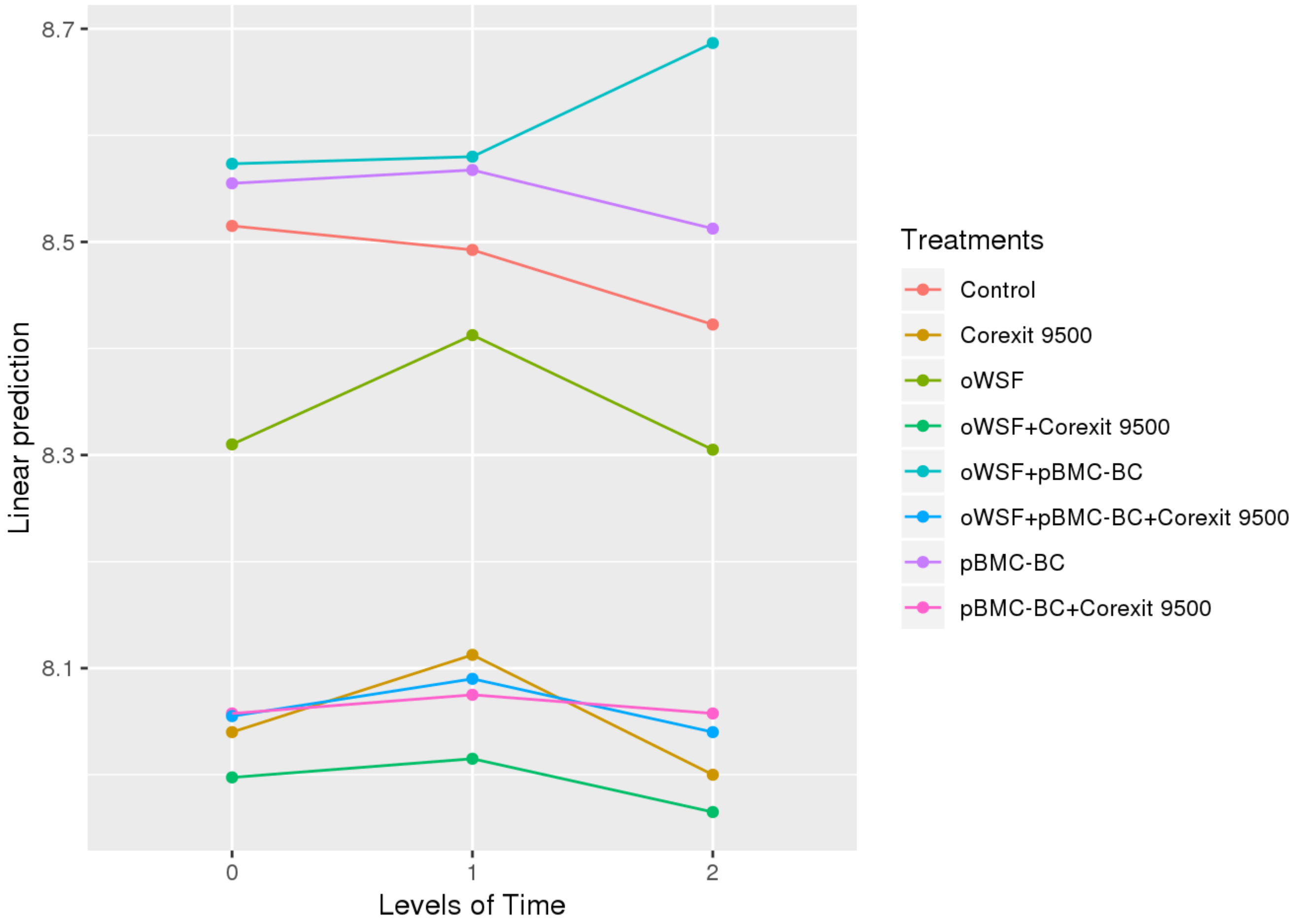


```
kable(CLD(marginal), row.names = F)
```

Treatments	emmean	SE	df	lower.CL	upper.CL	.group
oWSF+Corexit 9500	7.992500	0.0433810	23	7.902760	8.082240	1
Corexit 9500	8.050833	0.0433810	23	7.961093	8.140574	1
oWSF+pBMC-BC+Corexit 9500	8.061667	0.0433810	23	7.971926	8.151407	1
pBMC-BC+Corexit 9500	8.063333	0.0433810	23	7.973593	8.153074	1
oWSF	8.342500	0.0433810	23	8.252759	8.432241	2
Control	8.476667	0.0433810	30	8.388071	8.565263	23
pBMC-BC	8.545000	0.0433810	23	8.455260	8.634740	23
oWSF+pBMC-BC	8.613333	0.0500921	23	8.509710	8.716957	3

```
#DT::datatable(data.frame(marginal), options = list(pageLength = 20))
```

DOC

DOC differed over time (ANOVA) and post hoc analysis showed that T1 and T2 had significantly higher DOC than T0.

```
dat$DOC <- log(dat$DOC+1)
#Create model  with Treatment, Time, Treatment and Time interaction
#Include ID as a random effect
model <- lme(DOC ~ Time*Treatments, random = ~1|ID, data=dat, na.action = na.omit)
anova(model)
```

```
##                   numDF denDF  F-value p-value
## (Intercept)           1    46 431.7891  <.0001
## Time                  2    46  11.2364  0.0001
## Treatments            7    23   1.5146  0.2118
## Time:Treatments      14    46   1.2213  0.2933
```

```
#summary(model)

marginal <- emmeans(model, ~ Time)
plot(marginal, comparisons=T)
```

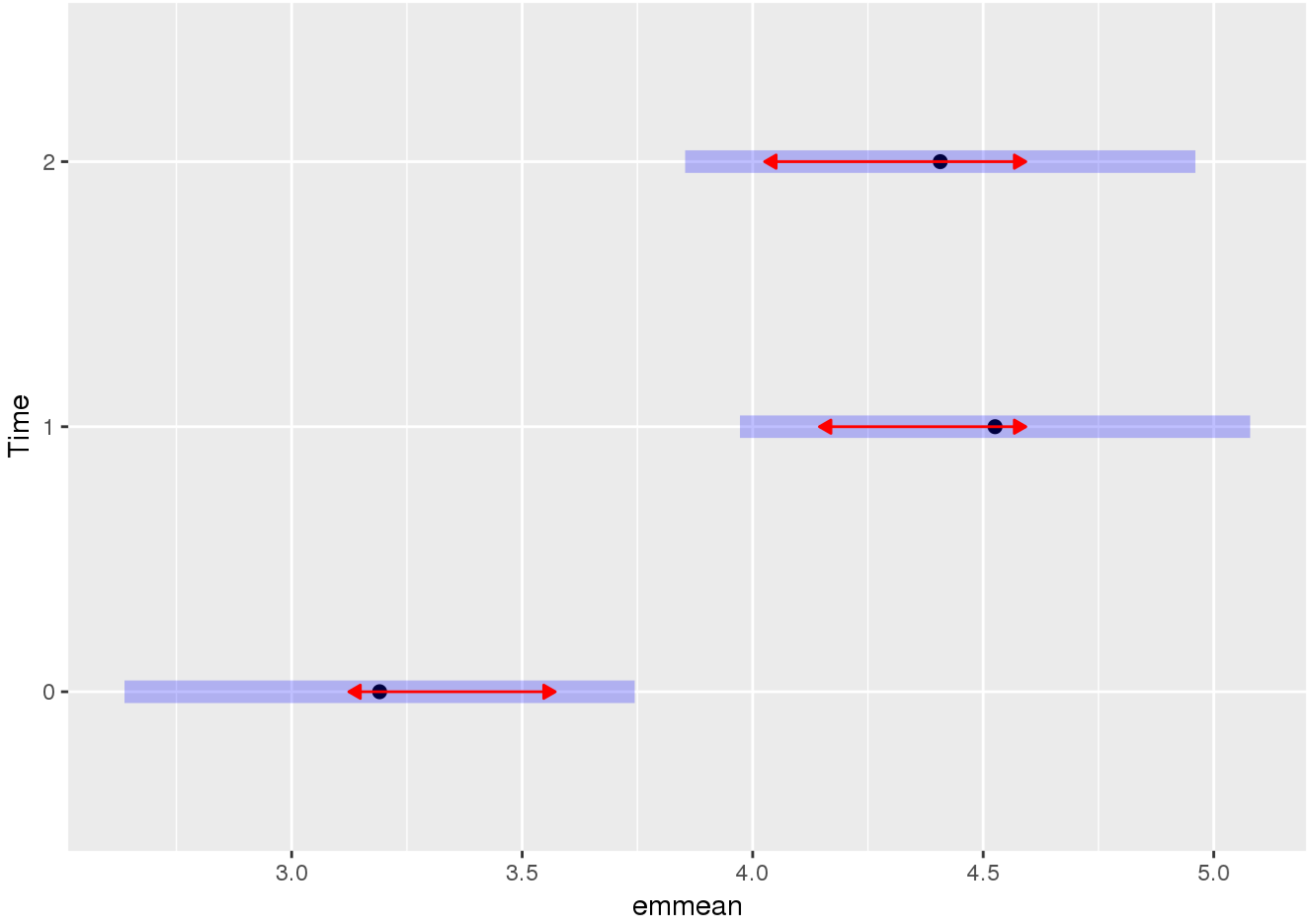


```
lsmip(model, Treatments~Time)
```

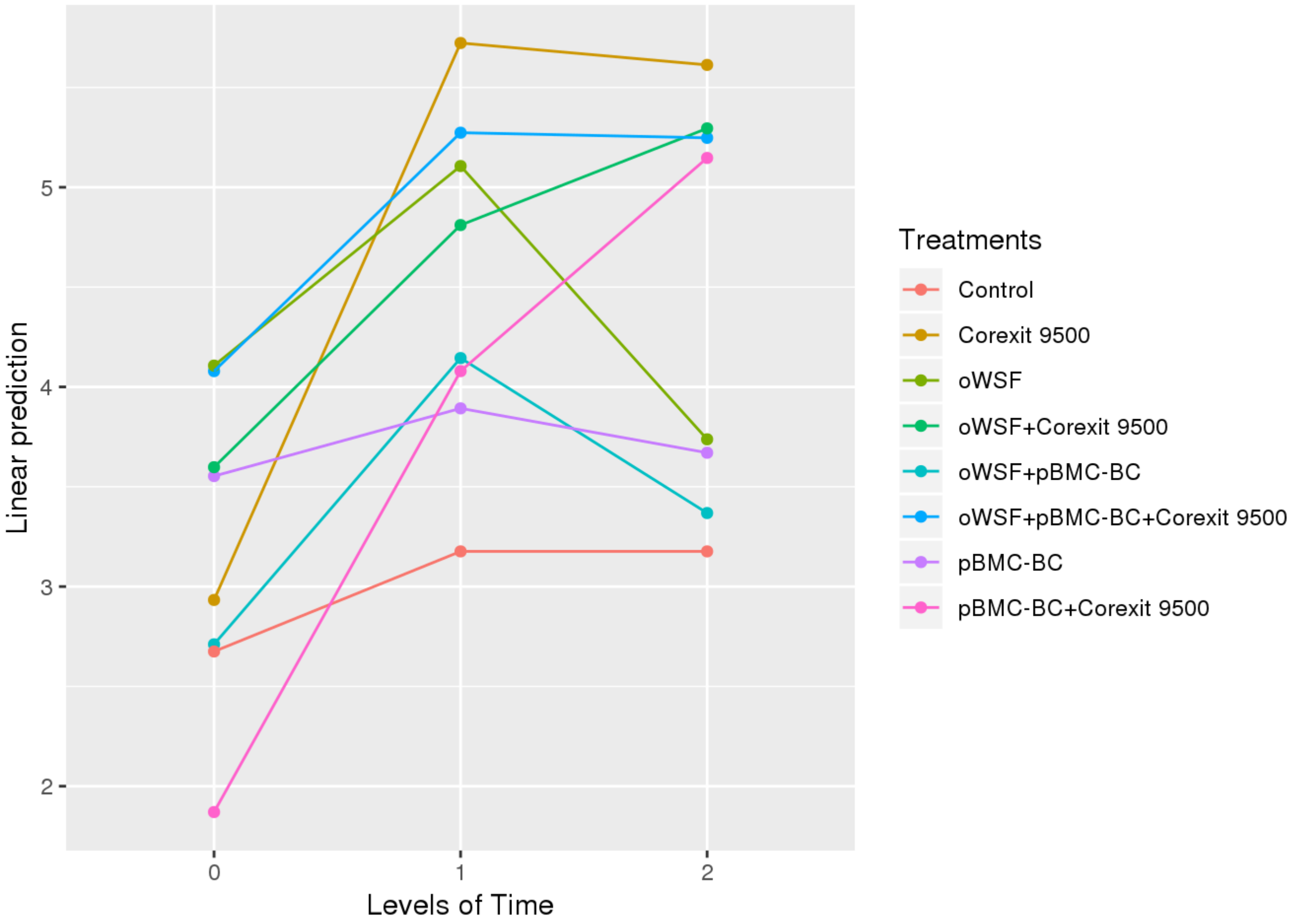


```
kable(CLD(marginal), row.names = F)
```

Time	emmean	SE	df	lower.CL	upper.CL	.group
0	3.190768	0.2674706	23	2.637463	3.744073	1
2	4.406819	0.2674706	23	3.853514	4.960124	2
1	4.525680	0.2674706	23	3.972375	5.078985	2

```
#DT::datatable(data.frame(marginal), options = list(pageLength = 20))
```

Ammonium

Ammonium differed over time (ANOVA) and post hoc analysis showed ammonium increased from T0 to T1, but then decreased by T2. Ammonium differed significantly between each time.

```
#Create model with Treatment, Time, Treatment and Time interaction
#Include ID as a random effect
model <- lme(Ammonium ~ Time*Treatments, random = ~1|ID, data=dat, na.action = na.omit)
anova(model)
```

```
##                  numDF denDF  F-value p-value
## (Intercept)          1    46 54.33377  <.0001
## Time                 2    46 14.86190  <.0001
## Treatments           7    23  1.33429  0.2794
## Time:Treatments     14    46  1.07213  0.4060
```

```
#summary(model)

marginal <- emmeans(model, ~ Time)
plot(marginal, comparisons=T)
```

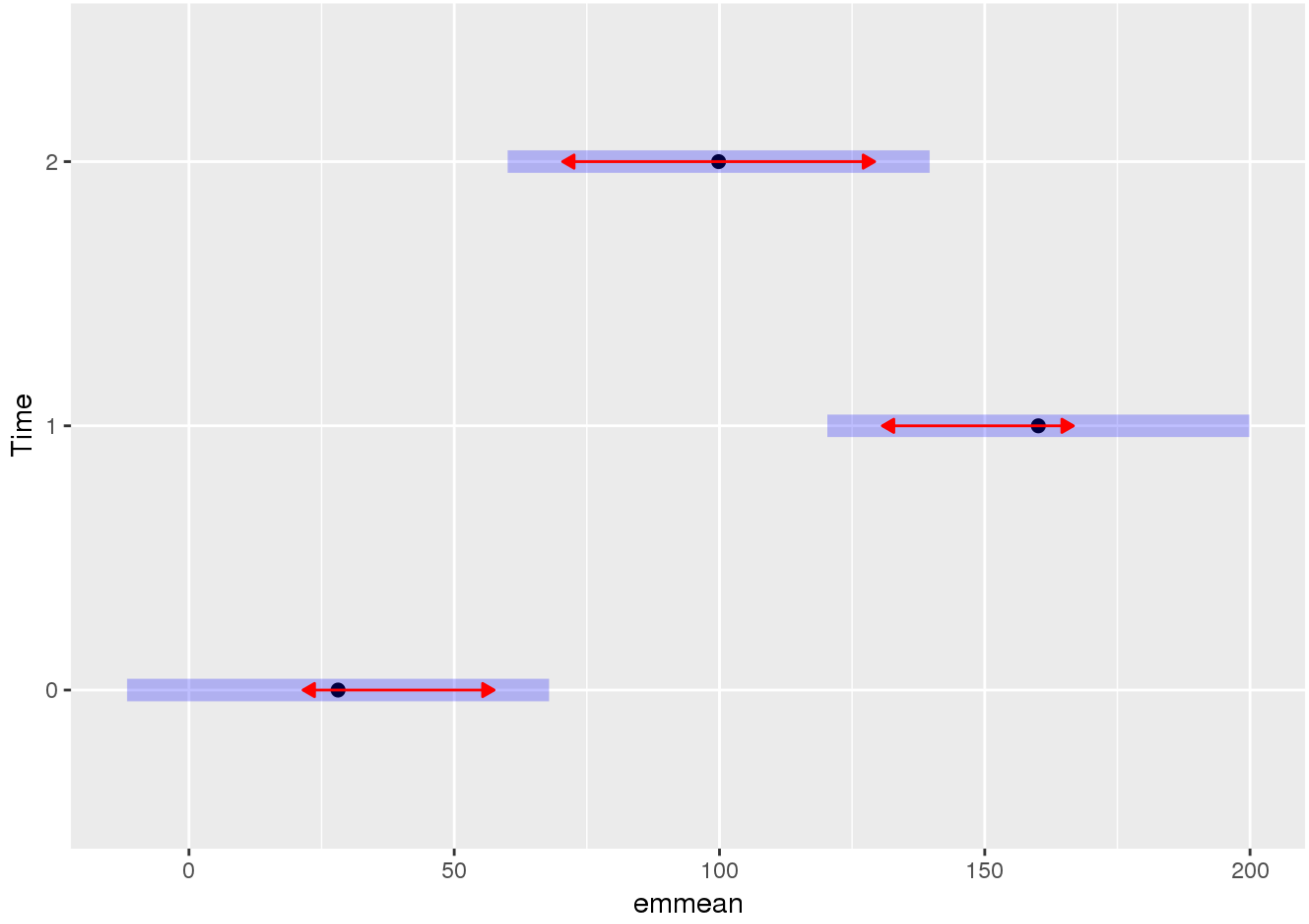


```
lsmip(model, Treatments~Time)
```

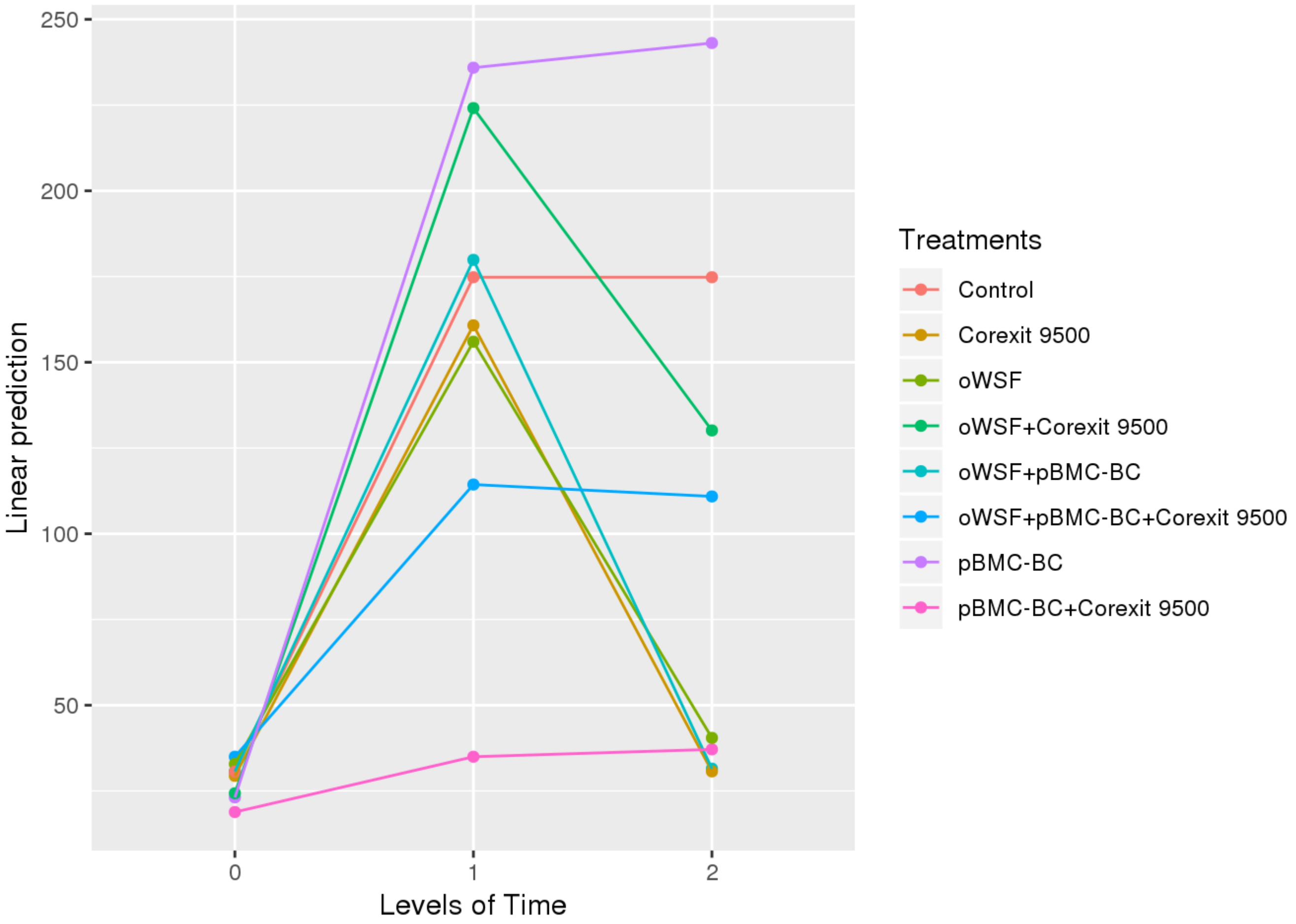


```
kable(CLD(marginal), row.names = F)
```

Time	emmean	SE	df	lower.CL	upper.CL	.group
0	28.10833	19.22297	23	-11.65741	67.87408	1
2	99.83750	19.22297	23	60.07175	139.60325	2
1	160.09896	19.22297	23	120.33321	199.86470	3

```
#DT::datatable(data.frame(marginal$contrasts), options = list(pageLength = 20))
```

Nitrate

Nitrate concenrtation differed over time (ANOVA). Post hoc analysis showed that increased from T0 to T1 but then decreased by T2. T0 and T1 differed from one another but T2 did not differ from either T0 or T1.

```
#Create model with Treatment, Time, Treatment and Time interaction
#Include ID as a random effect
model <- lme(Nitrate ~ Time*Treatments, random = ~1|ID, data=dat, na.action = na.omit)
anova(model)
```

```
##                   numDF denDF   F-value p-value
## (Intercept)           1    46 22.468590  <.0001
## Time                  2    46  6.880828  0.0024
## Treatments            7    23  0.353333  0.9198
## Time:Treatments      14    46  0.723966  0.7396
```

```
#summary(model)

marginal <- emmeans(model, ~ Time)
plot(marginal, comparisons=T)
```

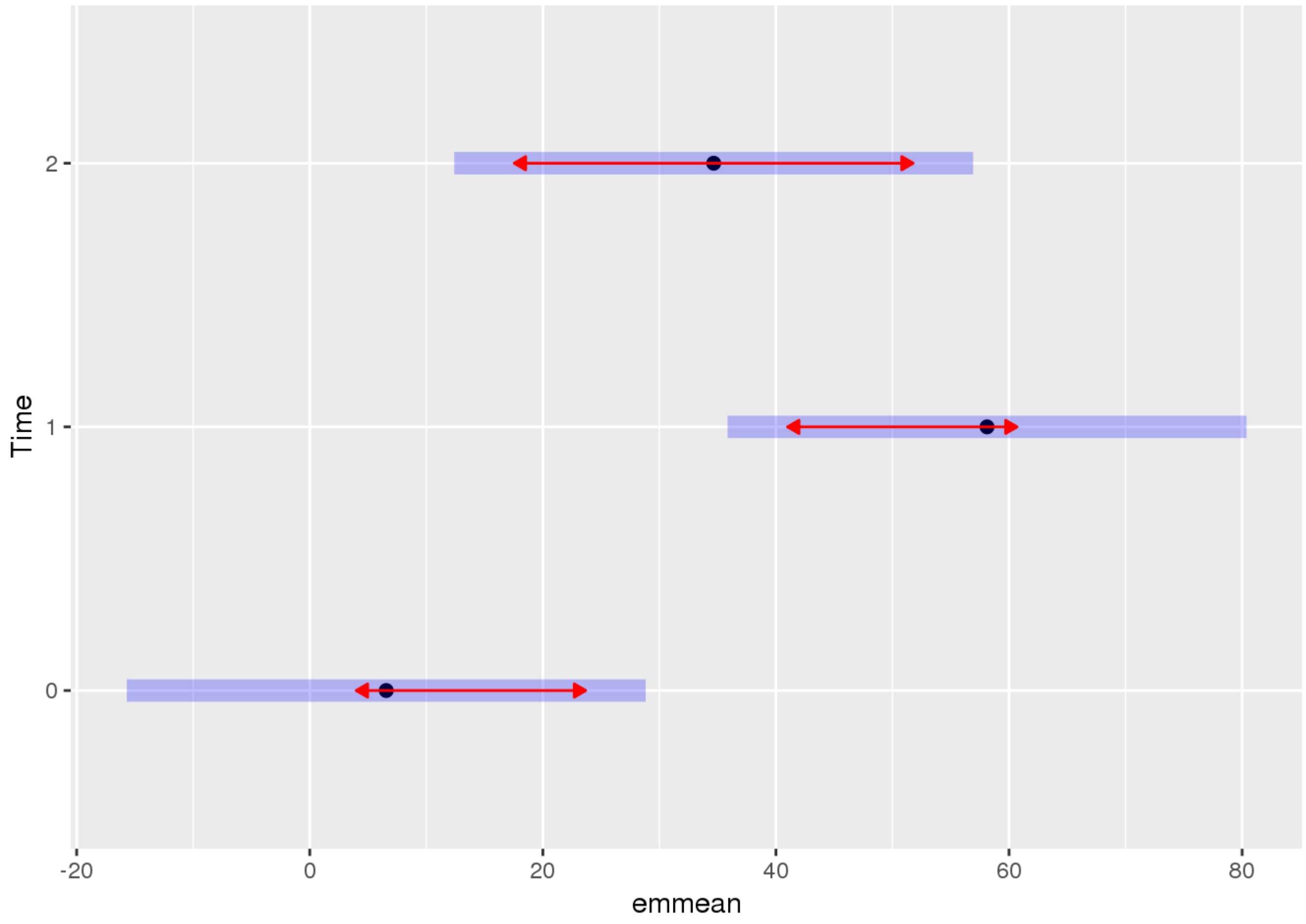


```
lsmip(model, Treatments~Time)
```

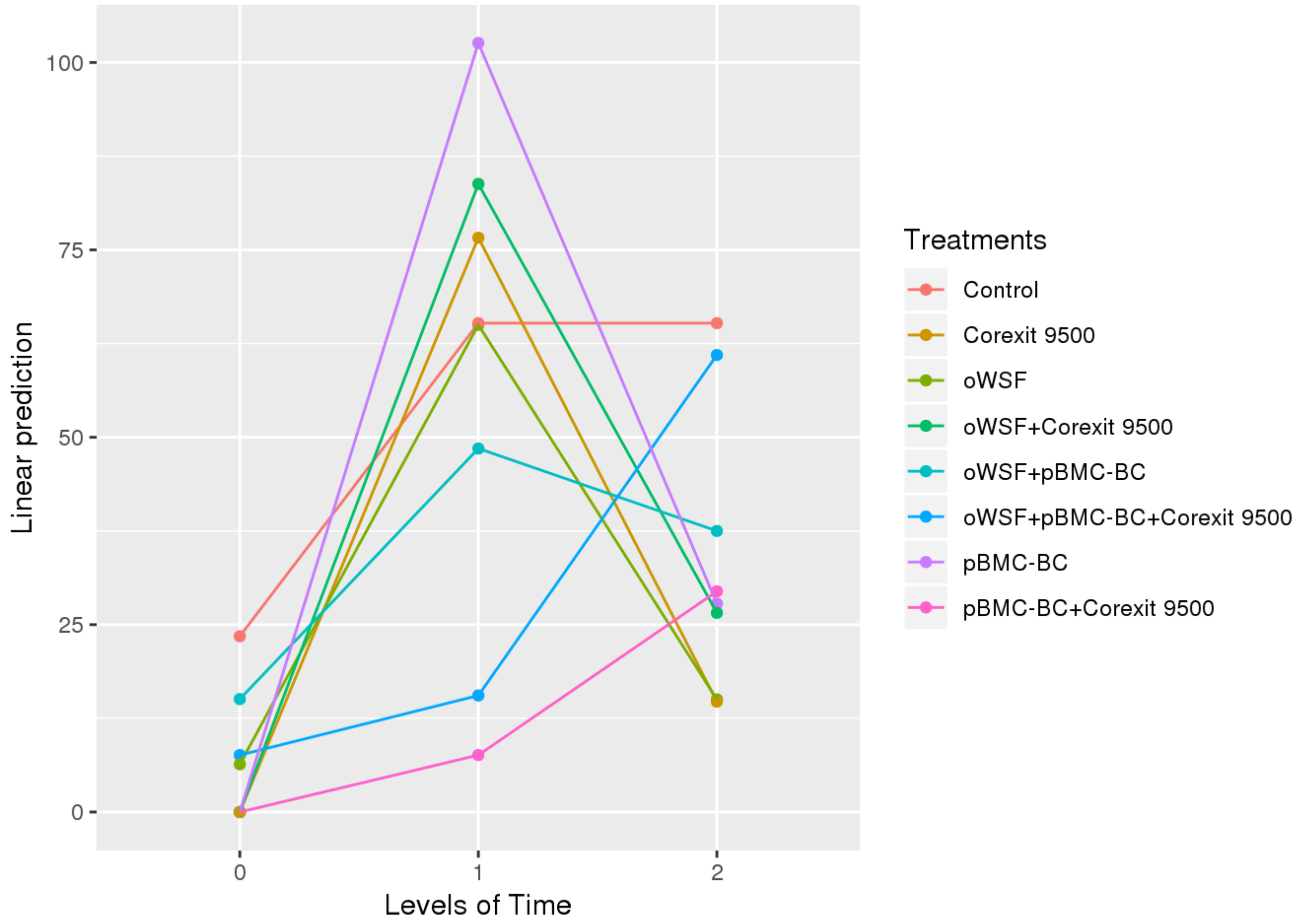


```
kable(CLD(marginal), row.names = F)
```

Time	emmean	SE	df	lower.CL	upper.CL	.group
0	6.561458	10.76017	23	-15.69766	28.82057	1
2	34.659375	10.76017	23	12.40026	56.91849	12
1	58.112500	10.76017	23	35.85339	80.37161	2

```
#DT::datatable(data.frame(marginal$contrasts), options = list(pageLength = 20))
```

Phosphate

ANOVA showed significant interactions among terms. Phosphate at T1 and T2 were significantly higher than at T0 within the dispersant-alone treatment (Corexit 9500).

```
#Create model with Treatment, Time, Treatment and Time interaction
#Include ID as a random effect
model <- lme(Phosphate ~ Time*Treatments, random = ~1|ID, data=dat, na.action = na.omit)
anova(model)
```

```
##                   numDF denDF   F-value p-value
## (Intercept)           1    46 108.13327  <.0001
## Time                  2    46  20.20636  <.0001
## Treatments            7    23   5.58713  0.0007
## Time:Treatments      14    46   3.37453  0.0009
```

```
#summary(model)

marginal <- emmeans(model, pairwise ~ Time * Treatments)
plot(marginal, comparisons=T)
```

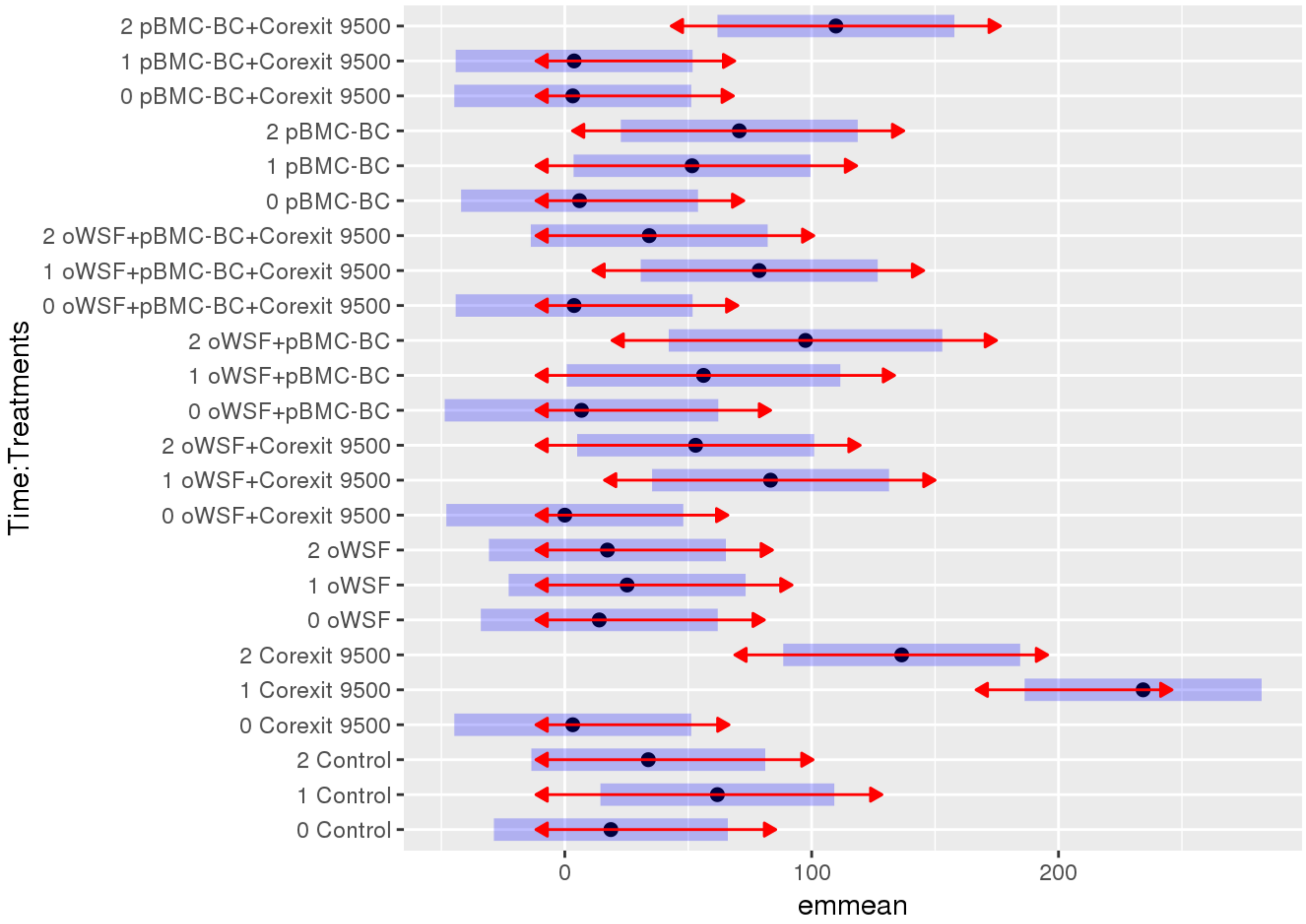


```
lsmip(model, Treatments~Time)
```

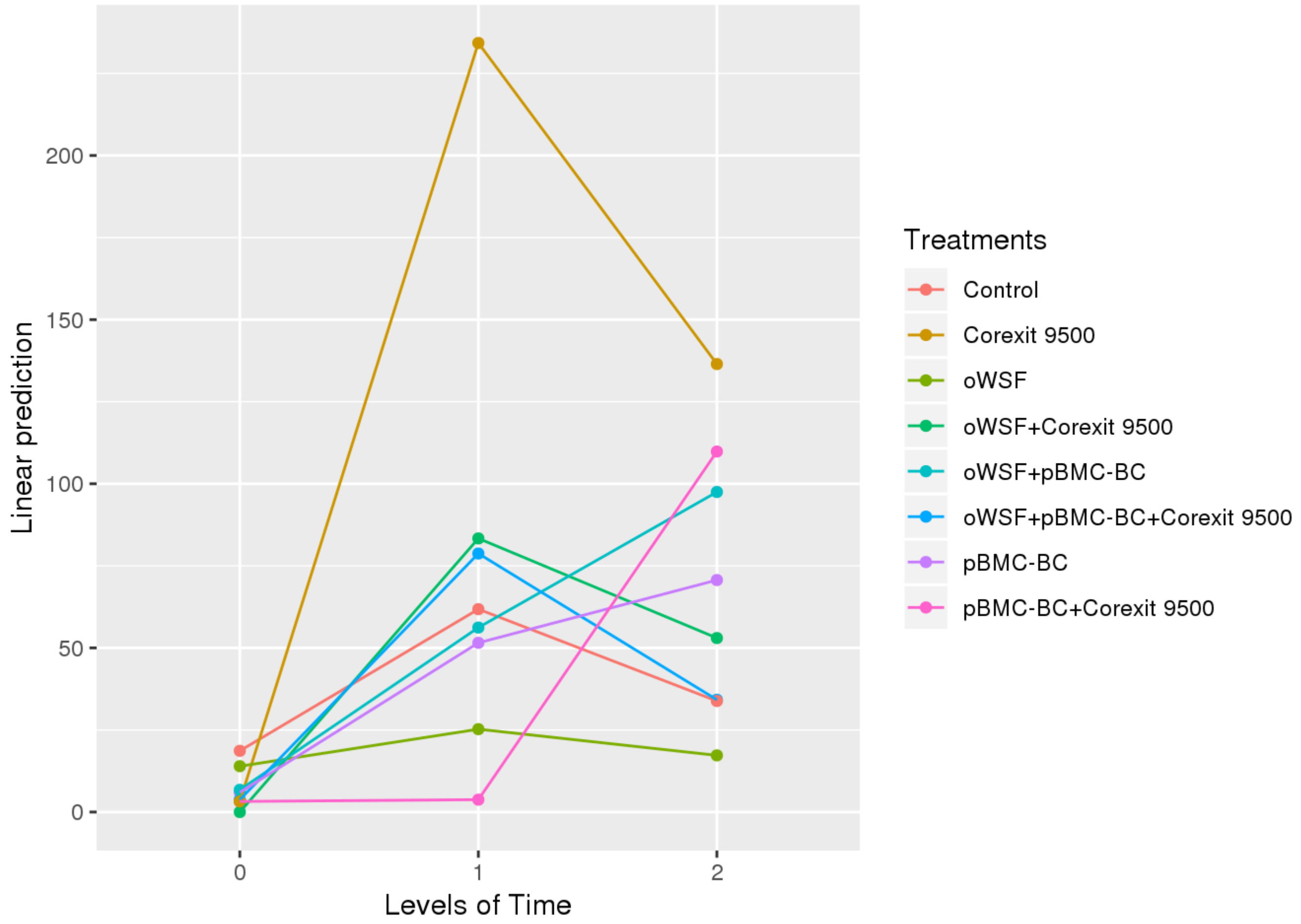


```
kable(CLD(marginal$emmeans), row.names = F)
```

Time	Treatments	emmean	SE	df	lower.CL	upper.CL	.group
0	oWSF+Corexit 9500	0.000000	23.19803	23	-47.9887806	47.98878	1
0	Corexit 9500	3.225000	23.19803	23	-44.7637806	51.21378	1
0	pBMC-BC+Corexit 9500	3.225000	23.19803	23	-44.7637806	51.21378	12
1	pBMC-BC+Corexit 9500	3.775000	23.19803	23	-44.2137806	51.76378	12
0	oWSF+pBMC-BC+Corexit 9500	3.775000	23.19803	23	-44.2137806	51.76378	12
0	pBMC-BC	5.975000	23.19803	23	-42.0137806	53.96378	12
0	oWSF+pBMC-BC	6.766667	26.78678	23	-48.6460041	62.17934	12
0	oWSF	13.962500	23.19803	23	-34.0262806	61.95128	12
2	oWSF	17.260000	23.19803	23	-30.7287806	65.24878	12
0	Control	18.650000	23.19803	30	-28.7266970	66.02670	12
1	oWSF	25.245000	23.19803	23	-22.7437806	73.23378	12
2	Control	33.825000	23.19803	30	-13.5516970	81.20170	12
2	oWSF+pBMC-BC+Corexit 9500	34.200000	23.19803	23	-13.7887806	82.18878	12
1	pBMC-BC	51.550000	23.19803	23	3.5612194	99.53878	12
2	oWSF+Corexit 9500	53.000000	23.19803	23	5.0112194	100.98878	12

Time	Treatments	emmean	SE	df	lower.CL	upper.CL	.group
1	oWSF+pBMC-BC	56.200000	26.78678	23	0.7873292	111.61267	12
1	Control	61.800000	23.19803	30	14.4233030	109.17670	12
2	pBMC-BC	70.675000	23.19803	23	22.6862194	118.66378	12
1	oWSF+pBMC-BC+Corexit 9500	78.750000	23.19803	23	30.7612194	126.73878	12
1	oWSF+Corexit 9500	83.350000	23.19803	23	35.3612194	131.33878	12
2	oWSF+pBMC-BC	97.500000	26.78678	23	42.0873292	152.91267	123
2	pBMC-BC+Corexit 9500	109.825000	23.19803	23	61.8362194	157.81378	123
2	Corexit 9500	136.475000	23.19803	23	88.4862194	184.46378	23
1	Corexit 9500	234.275000	23.19803	23	186.2862194	282.26378	3

```
#DT::datatable(data.frame(marginal$contrasts), options = list(pageLength = 20))
```

PAH

Levels of PAH increased significantly in treatments of oil with dispersants (both oWSF+Corexit 9500 and oWSF+pBMC-BC+Corexit 9500) at T1. However, by T2, PAH levels within these treatments had lowered to levels comparable to T0.

```
#Create model with Treatment, Time, Treatment and Time interaction
#Include ID as a random effect
model <- lme(PAH ~ Time*Treatments, random = ~1|ID, data=dat, na.action = na.omit)
anova(model)
```

```
##                 numDF denDF  F-value p-value
## (Intercept)         1    46 51.30686  <.0001
## Time                2    46  7.85888  0.0012
## Treatments          7    23 20.86227  <.0001
## Time:Treatments    14    46  3.31448  0.0011
```

```
#summary(model)

marginal <- emmeans(model, pairwise ~ Time * Treatments)
plot(marginal, comparisons=T)
```

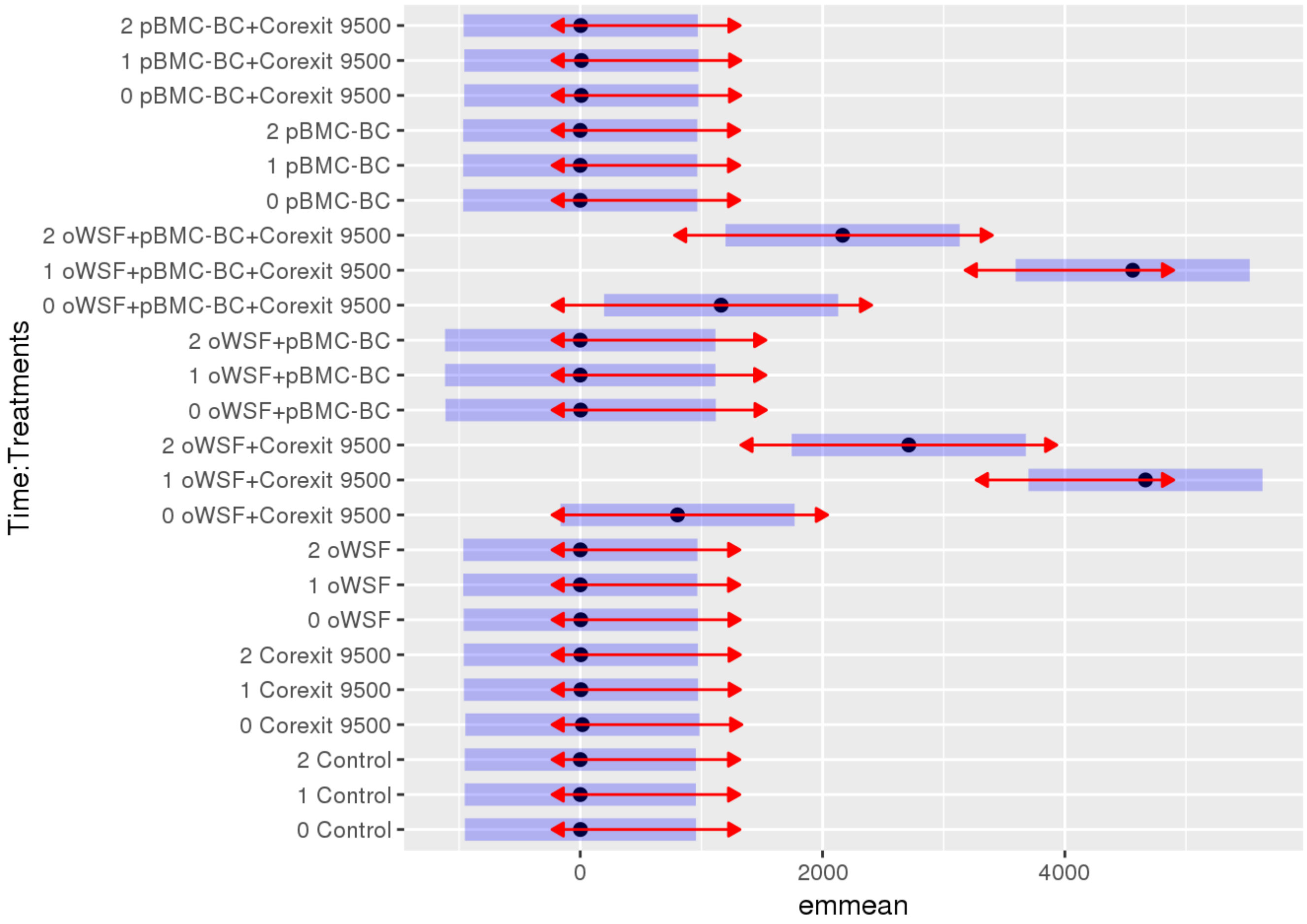


```
lsmip(model, Treatments~Time)
```

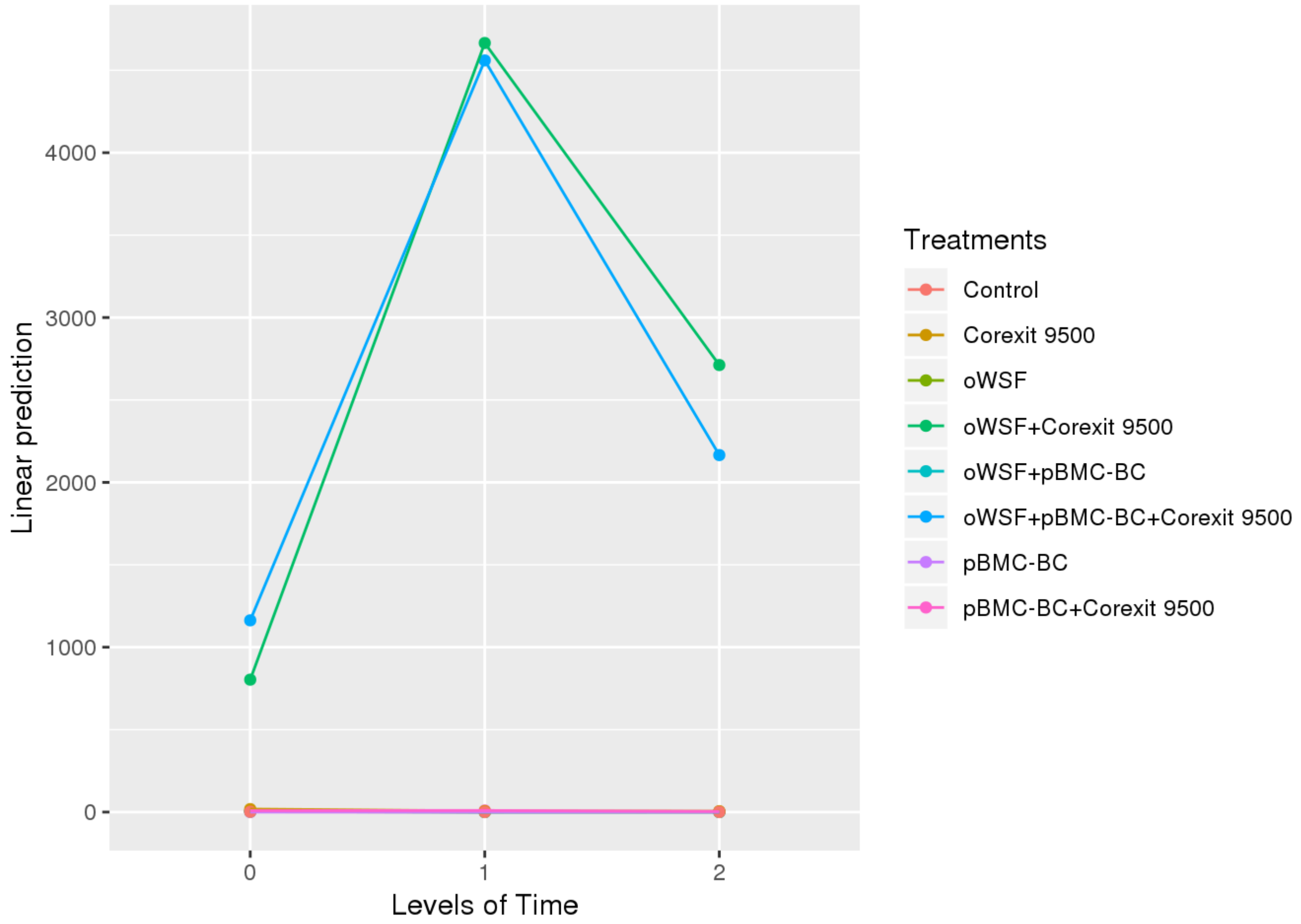


```
kable(CLD(marginal$emmeans), row.names = F)
```

Time	Treatments	emmean	SE	df	lower.CL	upper.CL	.group
1	oWSF+pBMC-BC	0.0340000	539.3090	23	-1115.6117	1115.6797	12
1	pBMC-BC	0.0615000	467.0553	23	-966.1160	966.2390	1
0	pBMC-BC	0.0862500	467.0553	23	-966.0912	966.2637	1
2	pBMC-BC	0.1067500	467.0553	23	-966.0707	966.2842	1
2	oWSF+pBMC-BC	0.1413333	539.3090	23	-1115.5043	1115.7870	12
1	Control	0.4850000	467.0553	30	-953.3692	954.3392	1
2	Control	0.5837500	467.0553	30	-953.2704	954.4379	1
1	oWSF	0.6197500	467.0553	23	-965.5577	966.7972	1
2	oWSF	1.2617500	467.0553	23	-964.9157	967.4392	1
0	Control	1.6350000	467.0553	30	-952.2192	955.4892	1
0	oWSF+pBMC-BC	3.3910000	539.3090	23	-1112.2547	1119.0367	12
2	pBMC-BC+Corexit 9500	3.9465000	467.0553	23	-962.2310	970.1240	1
0	oWSF	4.3650000	467.0553	23	-961.8125	970.5425	1
2	Corexit 9500	4.9907500	467.0553	23	-961.1867	971.1682	1
1	Corexit 9500	5.9947500	467.0553	23	-960.1827	972.1722	1

Time	Treatments	emmean	SE	df	lower.CL	upper.CL	.group
0	pBMC-BC+Corexit 9500	8.6052500	467.0553	23	-957.5722	974.7827	12
1	pBMC-BC+Corexit 9500	9.3965000	467.0553	23	-956.7810	975.5740	12
0	Corexit 9500	17.7292500	467.0553	23	-948.4482	983.9067	12
0	oWSF+Corexit 9500	803.1927500	467.0553	23	-162.9847	1769.3702	12
0	oWSF+pBMC-BC+Corexit 9500	1162.9485000	467.0553	23	196.7710	2129.1260	12
2	oWSF+pBMC-BC+Corexit 9500	2165.5532500	467.0553	23	1199.3758	3131.7307	123
2	oWSF+Corexit 9500	2711.7595000	467.0553	23	1745.5820	3677.9370	23
1	oWSF+pBMC-BC+Corexit 9500	4559.7662500	467.0553	23	3593.5888	5525.9437	3
1	oWSF+Corexit 9500	4665.4685000	467.0553	23	3699.2910	5631.6460	3

```
#DT::datatable(data.frame(marginal$contrasts), options = list(pageLength = 20))
```
