# Supplementary figures and images for: Multi-domain probiotic consortium as an alternative to chemical remediation of oil spills at coral reefs and adjacent sites
Source: Microbiome. 2021 May 21;9:118. doi: 10.1186/s40168-021-01041-w (PMC8138999; doi:10.1186/s40168-021-01041-w)

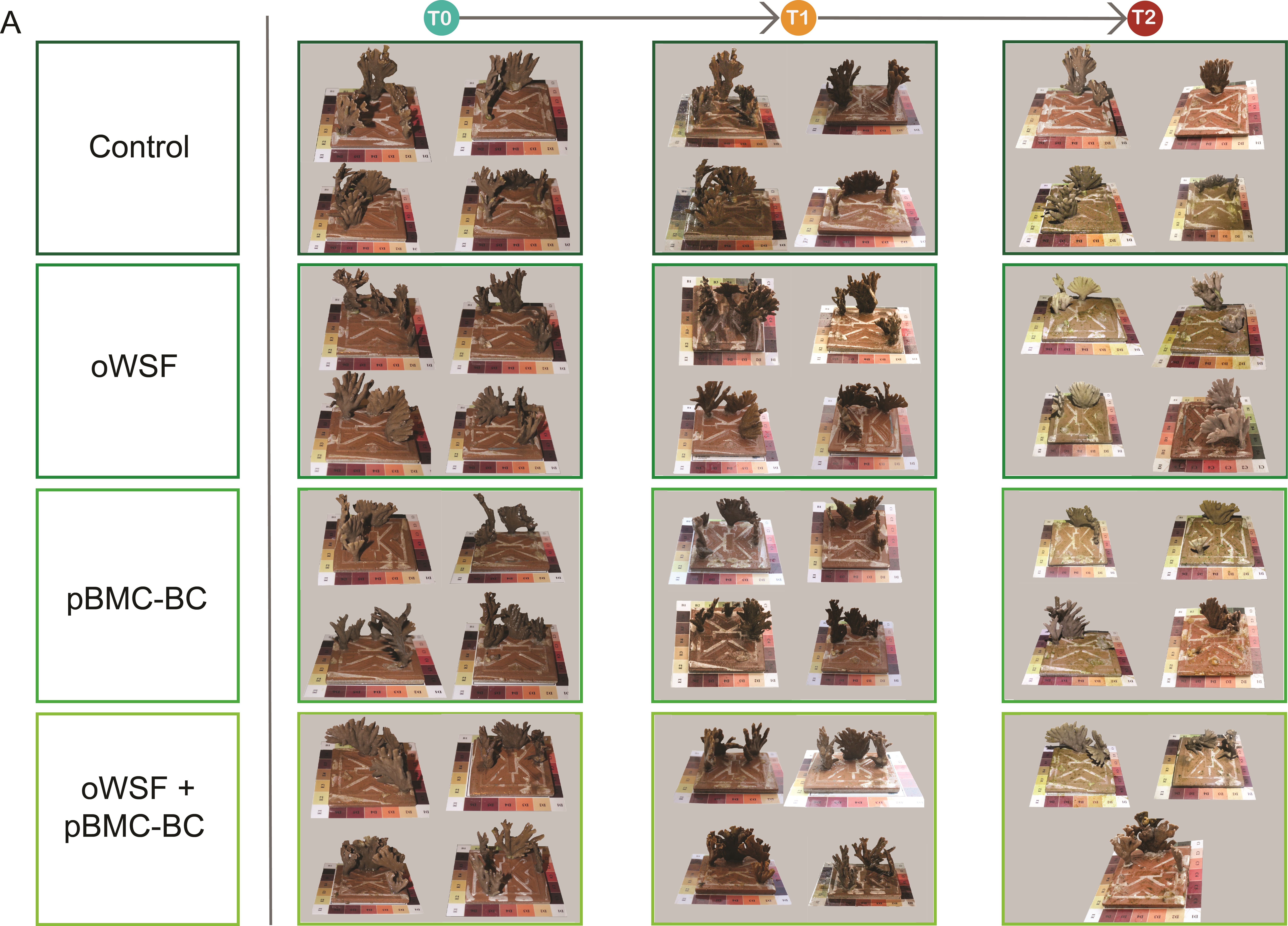

Supplement: Supplementary file 3 — Additional file 2: Figure S1. Photographs of all replicates for each treatment at T0, T1 and T2 on Coral Watch Health Card. A) Treatments without Corexit 9500; B) Treatments with Corexit 9500. [file 40168_2021_1041_MOESM3_ESM.zip › Figure S1_A.jpg]

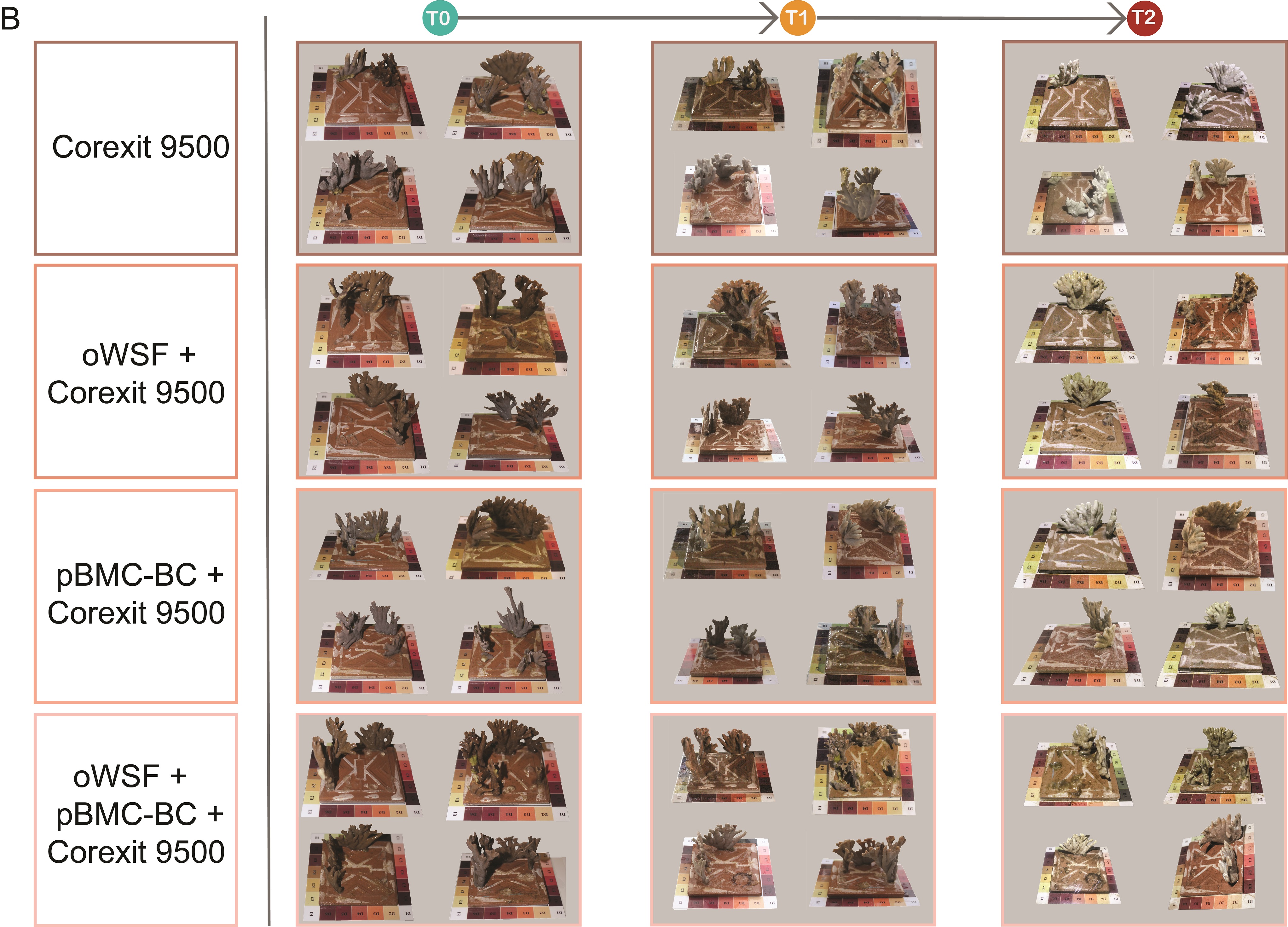

Supplement: Supplementary file 3 — Additional file 2: Figure S1. Photographs of all replicates for each treatment at T0, T1 and T2 on Coral Watch Health Card. A) Treatments without Corexit 9500; B) Treatments with Corexit 9500. [file 40168_2021_1041_MOESM3_ESM.zip › Figure S1_B.jpg]

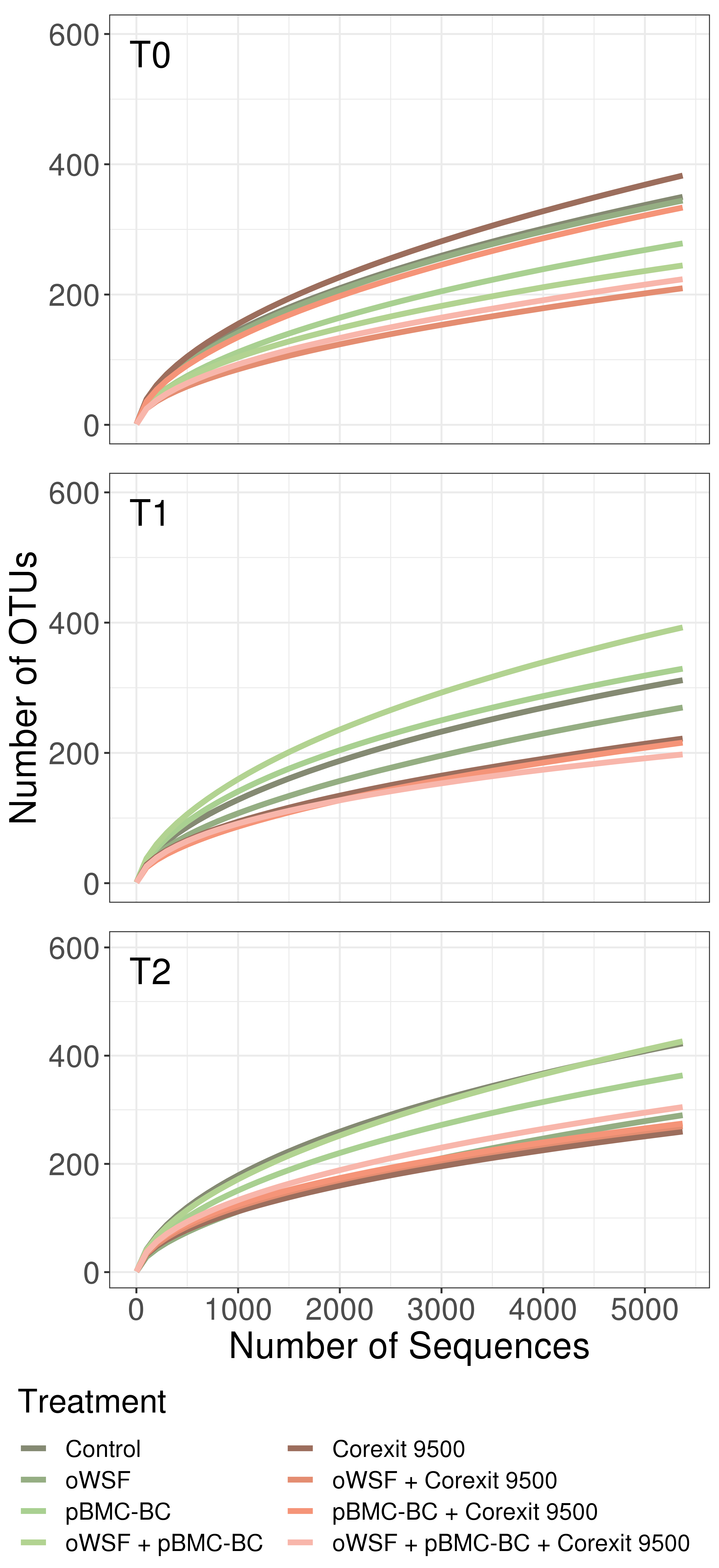

Supplement: Supplementary file 4 — Additional file 3: Figure S2. Rarefaction curve representing the α-diversity analysis of the bacterial community, based on the partial sequences of 16S subunits of ribosomal RNA in all treatments over time. [file 40168_2021_1041_MOESM4_ESM.png]

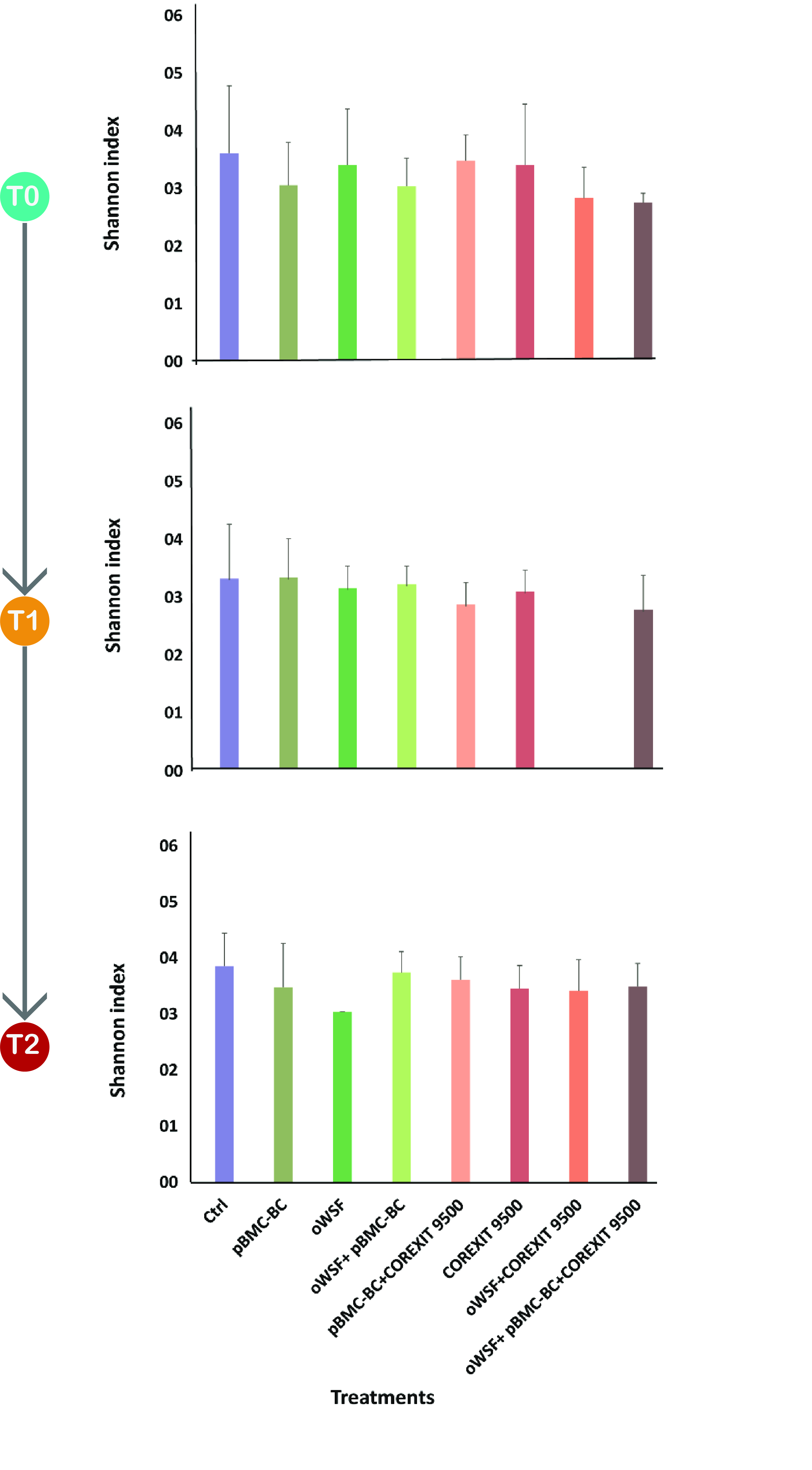

Supplement: Supplementary file 6 — Additional file 5: Figure S4. Estimation of OTU richness through the Shannon diversity index, in all treatments over the experimental period. [file 40168_2021_1041_MOESM6_ESM.jpg]

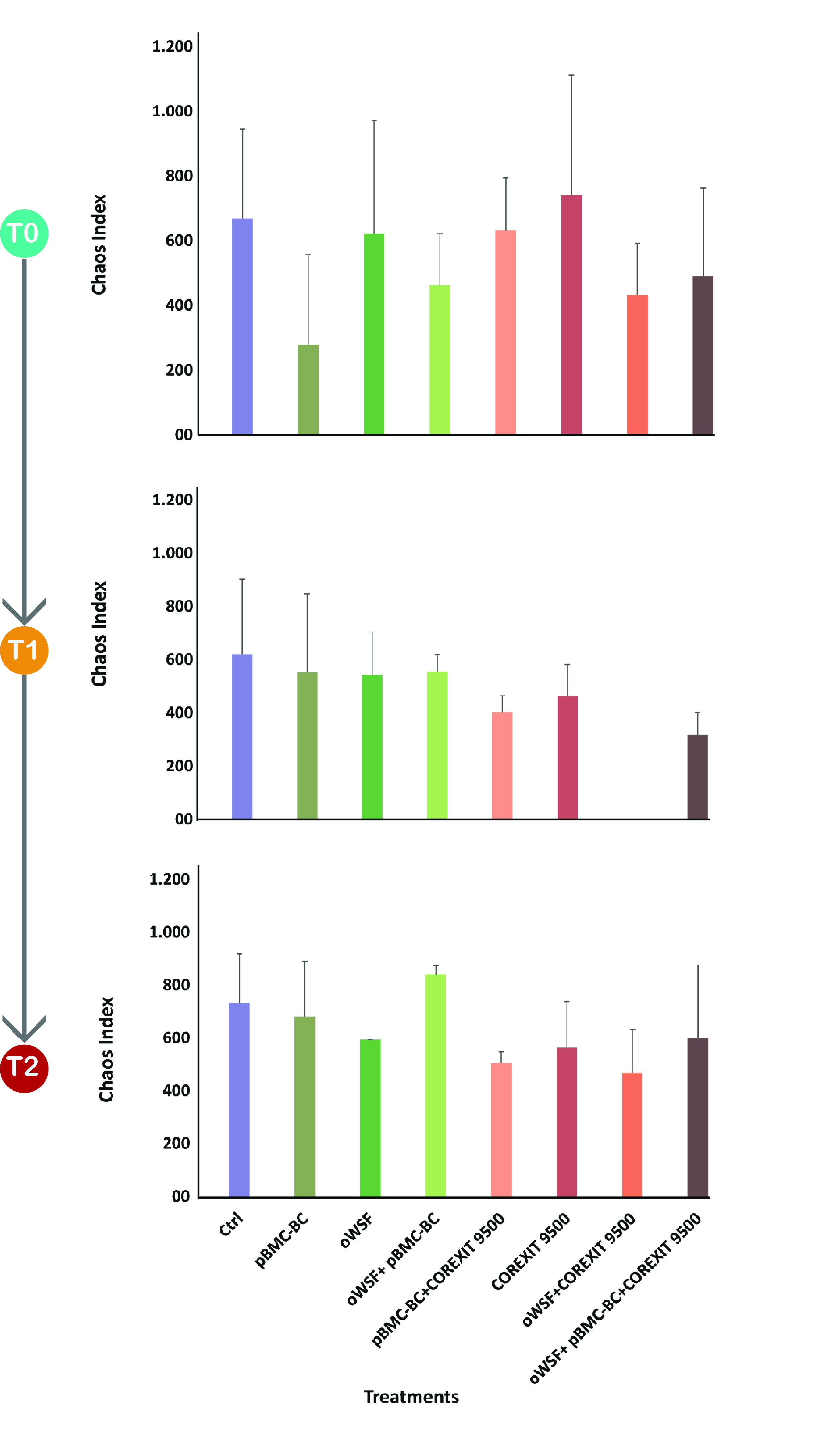

Supplement: Supplementary file 7 — Additional file 6: Figure S5. Estimation of OTU richness through the Chao diversity index, in all treatments over the experimental period. [file 40168_2021_1041_MOESM7_ESM.jpg]

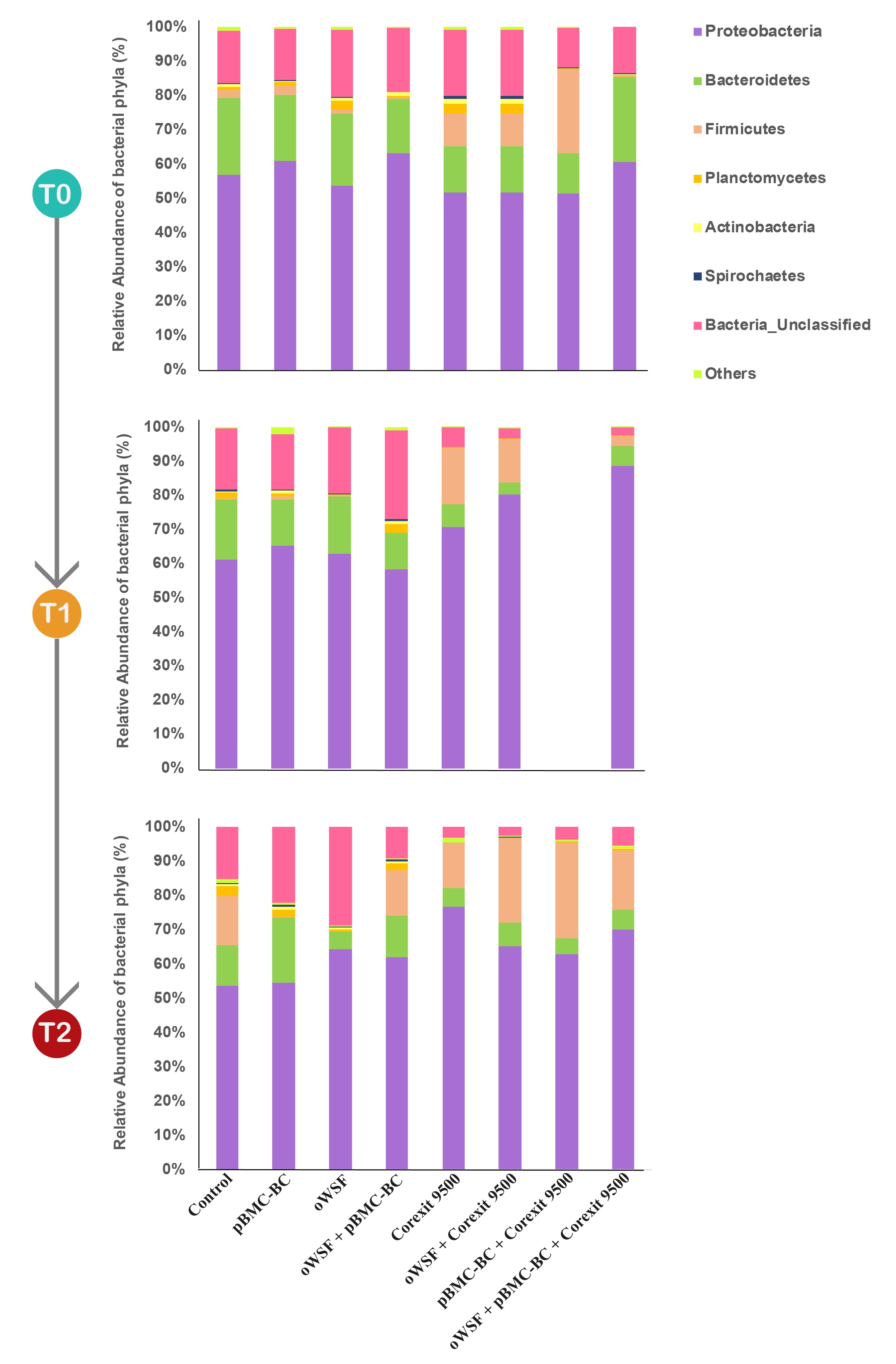

Supplement: Supplementary file 8 — Additional file 7: Figure S6. Taxonomic comparison of bacterial phyla, based on the DNA sequences obtained from the partial sequence of the 16S subunit of ribosomal RNA in all treatments over time. Note: the pBMC-BC+Corexit9500 sample in T1 is missing, due to the loss of low-quality sequences. [file 40168_2021_1041_MOESM8_ESM.jpg]

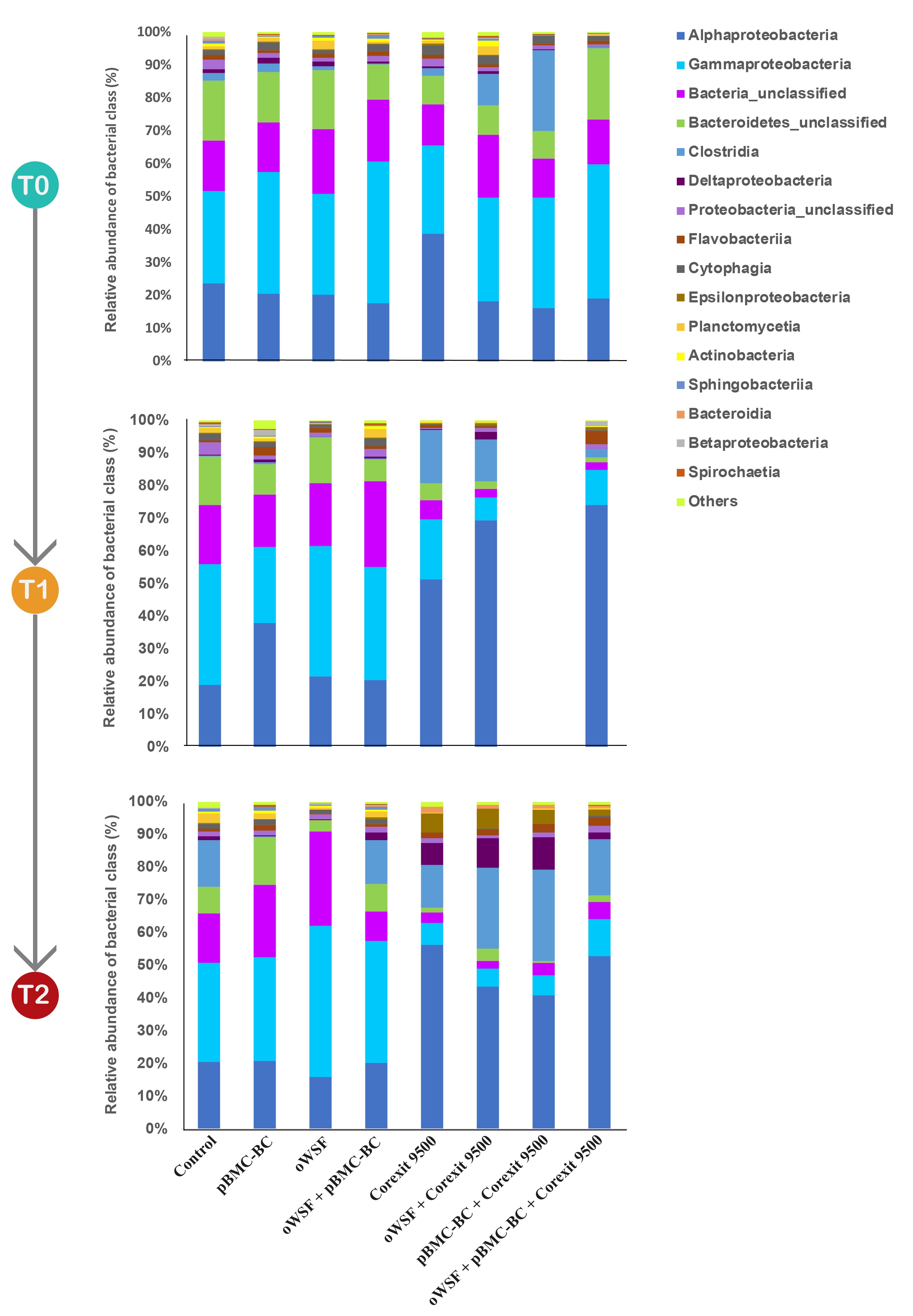

Supplement: Supplementary file 9 — Additional file 8: Figure S7. Taxonomic comparison of bacterial classes, based on the DNA sequences obtained from the partial sequence of the 16S subunit of ribosomal RNA in all treatments over time. Note: the pBMC-BC+Corexit9500 sample in T1 is missing, due to the loss of low-quality sequences. [file 40168_2021_1041_MOESM9_ESM.jpg]

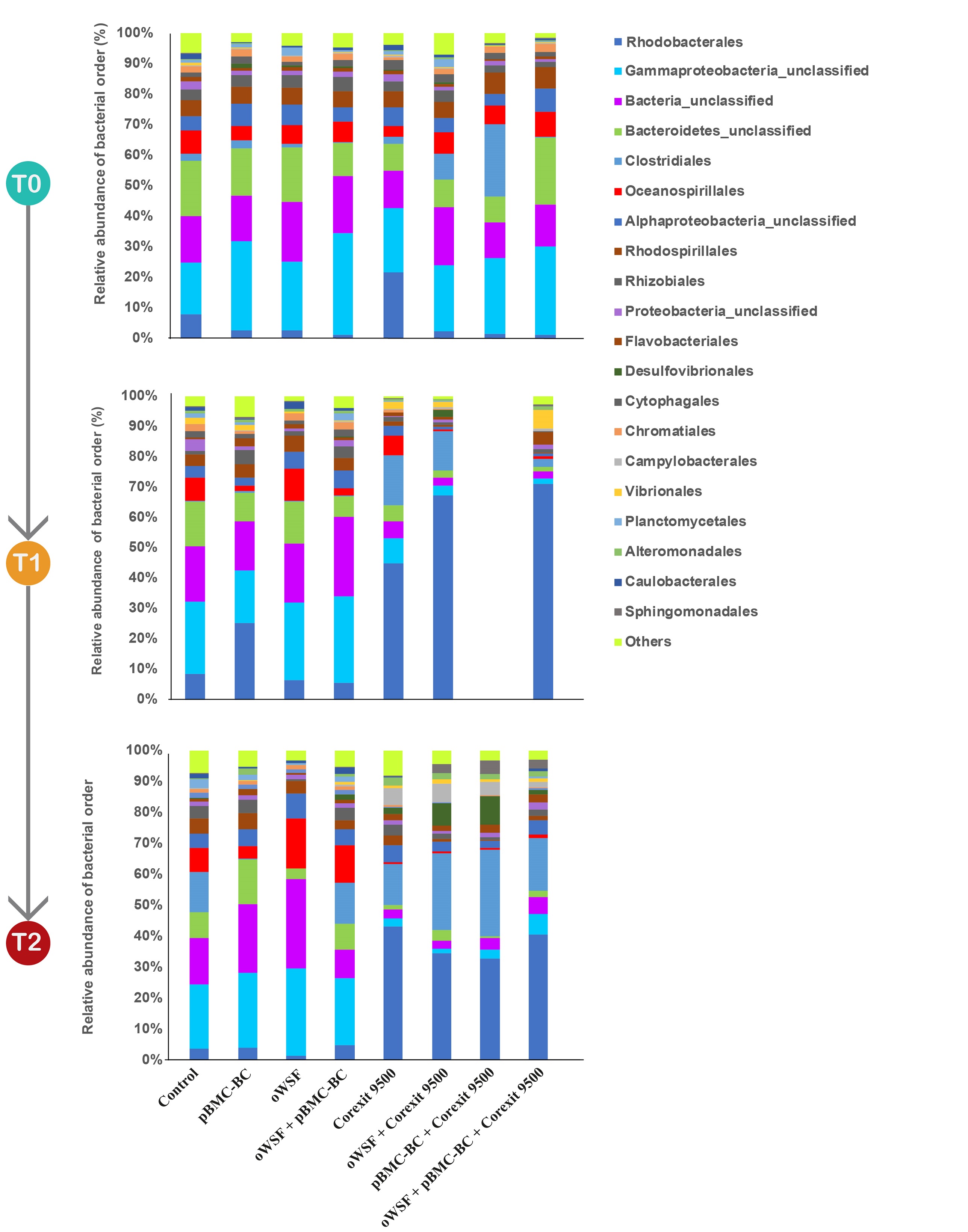

Supplement: Supplementary file 10 — Additional file 9: Figure S8. Taxonomic comparison of bacterial orders, based on the DNA sequences obtained from the fragment of the 16S subunit of ribosomal RNA in all treatments over time. Note: the pBMC-BC+Corexit9500 sample in T1 is missing, due to the loss of low-quality sequences. [file 40168_2021_1041_MOESM10_ESM.jpg]
