# Supplementary material for: Multi-domain probiotic consortium as an alternative to chemical remediation of oil spills at coral reefs and adjacent sites
Source: Microbiome. 2021 May 21;9:118. doi: 10.1186/s40168-021-01041-w (PMC8138999; doi:10.1186/s40168-021-01041-w)
Supplement: Supplementary file 14 — Additional file 13. [file 40168_2021_1041_MOESM14_ESM.docx]

Supplementary Methods for:

**Multi-domain probiotic consortium as an alternative to chemical remediation of oil spill at coral reefs and adjacent sites**

Denise P. da Silva. ^1+^, Helena D.M Villela. ^1+^, Henrique F. dos Santos^3^, Gustavo A. S. Duarte^1,2^, José Roberto Ribeiro^1^, Angela Michelato^1^, Caren L.S. Vilela^1^, Phillipe M. Rosado ^1^; Carolline S. Fazolato B.^1^; Erika P. Santoro^1^, Flavia L. Carmo^1^ Dalton S. Ximenes^4^, Adriana U. Soriano^4^, Caio T. C.C. Rachid.^5^, Rebecca L. Vega Thurber^6*^, Raquel S.Peixoto^1, 2,7*^

^+^Both authors contributed equally to this work

^1^LEMM, Laboratory of Molecular Microbial Ecology, Institute of Microbiology Paulo de Góes, Federal University of Rio de Janeiro (UFRJ), Brazil.

^2^ IMAM-AquaRio – Rio de Janeiro Aquarium Research Center, Rio de Janeiro, Brazil

^3^ Department of Marine Biology, Fluminense Federal University (UFF), Brazil

^4^ Biotechnology Section, Leopoldo Américo Miguez de Mello Research Center, Petrobras, Rio de Janeiro, Brazil.

^5^LABEM, Institute of Microbiology Paulo de Góes, Federal University of Rio de Janeiro (UFRJ), Brazil.

^6^ Department of Microbiology, Oregon State University, Oregon, USA.

^7^ Genome Center, University of California Davis, USA

*Corresponding authors

- Raquel Silva Peixoto

Phone: +55 2125626740; e-mail: raquelpeixoto@micro.ufrj.br

Address: Federal University of Rio de Janeiro, Av Carlos Chagas Filho, 373 CCS, Bl E, Rio de Janeiro, RJ, 21941902, Brazil;

- Rebecca Vega Thurber

Phone: +1 541-737-1851; e-mail: rebecca.vega-thurber@oregonstate.edu

Address: Nash Hall 226, OSU, Corvallis, OR 97331, USA.

**Materials and Methods**

The supplementary materials are divided in two parts: **Part I** contains the development of the specific media to select oil-degrading multi-domain microbial consortium and **Part II** describes coral collection for experimental exposure and 16S rRNA gene sequencing library preparation methods.

**Part I:**

**Media preparation**

To develop of an oil-degrading multi-domain microbial consortium we first prepared selective media. Crude oil was added in 500 mL of sterile saline solution (0.85%), to a final concentration of 2%. The mixture was placed in a 2 L culture flask under constant agitation of 180 rpm at 24 ^°^C for 48 h. Oil water soluble and insoluble fractions (oWSF and oWIF, respectively) were separated using a pump and a sterile 25 mL pipette. For bacterial isolation, BH agar medium (Bushnell-Haas) (Sigma- Aldrich, Missouri USA) was supplemented with oWSF or oWIF, independently, as the only carbon source. In addition, two salinity concentrations were used in these media: 2.5 % and 8 % NaCl. A total of 4 types of media were used: BH-oWSF 2.5 % NaCl, BH-oWSF 8 % NaCl, BH-oWIF 2.5 % NaCl and BH-oWIF 8 % NaCl. Media for filamentous fungi isolation was BH broth (Sigma-Aldrich) supplemented with glucose 0.1 % and yeast extract 0.05 % (BH-G). oWSF and oWIF at concentration of 10% were filtered and each membrane was added to BH-G to select the strains able to grow in the presence of oil. All four BH agar media used for bacterial isolation and one supplemented with malt extract 2 % were used for spreading plates. All eight types of isolation media for fungi contained amoxicillin 0.05 % and chloramphenicol 0.04 %. The MS medium (malt extract 2 %, agar 2 %, NaCl 2.5 %) and MS broth were used to cultivate yeasts and filamentous fungi strains.

**Oil -degrading consortium identification**

Selected bacterial strains were submitted to genomic DNA extraction followed by PCR amplification. Two milliliters of each strain were grown in MB medium (Marine Broth 2216, Himedia Laboratories, Mumbai, India), and genomic DNA was extracted with the Wizard Genomic DNA Purification kit (Promega, California, USA). Primers 27f (5′AGAGTTTGATCATGGCTCAG 3′) and 1492r (5′ GTTTACCTTGTTACGACTT 3′) [35] were used for amplification of the 16S rRNA gene. PCR was performed in a final volume of 50 μL, using 5 μL of buffer (10X), 2 mM MgCl_2_, 0.2 mM dNTPs, 5 mM of each primer, 10 ng of genomic DNA and 2.5 U of Taq DNA polymerase (Promega, USA). The thermal cycling steps were a first denaturation cycle at 94 °C for 4 min; 35 cycles at 94 °C for 1 min followed by a 50 °C for 1 min and 72 °C for 2.5 min; a final extension cycle for 10 min at 72 °C.

For filamentous fungi and yeast DNA extraction and PCR, a loop of biomass of each strain was used for extraction. We evaluated the D1/D2 regions of the subunit of the 28S ribosomal RNA gene and the internal transcribed spacer (ITS) region. Primers for filamentous fungi, were: ITS1 (5’ TCCGTAGGTGAACCTGCGG 3’) and ITS4 (5’ TCCTCCGCTTATTGATATGC 3’) [36], and ITS+D1D2/LSU for yeast, using ITS1 and NL4 (5’ GGTCCGTGTTTCAAGACGG 3’) [37]. Amplification reactions followed the same protocol used for bacteria, but the thermal cycling conditions were: first step of denaturation at 94º C, followed by 30 cycles of 94 ºC for 30 s, 52 ºC for 30 s and 72 ºC for 1 min, and a final step of 72 ºC for 5 min.

Amplicon integrity was verified by 1% agarose gel electrophoresis, and gel purified from using the GFX PCR DNA and Gel Band Purification kit according to the manufacture recommendations (GE Healthcare, Illinois, USA). Quantification of purified PCR products was performed using a Qubit® 2.0 Fluorometer dsDNA type fluorometer (Invitrogen, USA). Amplicons were sequenced (Macrogen Inc., Seoul, South Korea), with the primers 27f (5′-AGA GTT TGA TCA TGG CTC AG-3′), 1492r (5′-GTT TAC CTT GTT ACG ACT T-3′), 532f (5′-CGT GCC AGC AGC CGC GGT AA-3′) and 907r (5′-CCG TCA ATT CMT TTG AGT TT-3′) [35] for bacteria strains, ITS1 and ITS4 for filamentous fungi and ITS1, ITS4, NL1 (5´-GCATATCAATAAGCGGAGGAAAAG- 3´) and NL4 for yeast strains. Low-quality sequences were removed after processing with the Ribosomal Database Project Sanger Pipeline (RDP; <http://pyro.cme.msu.edu>), and further assembled into contigs with the program Bioedit 7.0.5.355.

**Part II:**

**Experimental design**

Collection of experimental corals

Four individual colonies of the hydrocoral *Millepora alcicornis* were collected in different sampling sites at João Fernandes Beach, Armação dos Búzios, Rio de Janeiro, Brazil (22°44'29.95"S; 41°52'35.62"W). During transport, the fragments were kept submerged in renewed seawater, under shade cloth with a blocking capacity of 70 % of the solar incidence, and at the same water temperature as the collection site. The four genotypes of the sampled *M. alcicornis* were fragmented into nubbins of approximately 5 cm in length and 6 g of mass, glued on tiles with a drop of cyanoacrylate (Super Bonder Glue 3, Loctite, São Paulo, Brazil), and placed in aquariums. To guarantee the validation of random effect, one coral nubbin from each one of the four genotypes collected were placed in same aquariums.

**Bacterial community associated with the hydrocoral *M. alcicornis***

Sample processing and total DNA extraction

To evaluate the diversity of the microbial communities associated with *M. alcicornis* hydrocoral under the impact of different remediation strategies, 0.5 g of coral fragment of each replicate was macerated with mortar and pestle using liquid nitrogen. Total DNA extraction from the coral was performed using the Qiagen DNAeasy Power Soil kit (Qiagen, Hilden, Germany). following the manufacturer's protocol. The extracted DNA was quantified by fluorimetry, with Qubit® 2.0 Fluorometer dsDNA type fluorometer (Invitrogen, California, USA).

High throughput sequencing and library preparation for 16S rRNA gene amplicon analysis

Bacterial communities were analyzed by sequencing the V4 variable region of the 16S rRNA gene. Single-step PCR was performed with primers 515F (5' GTGCCAGCMGCCGCGGTAA 3 ') and 806R (5' GGACTACHVGGGTWTCTAAT 3 ') [45] using AccuStart ToughMix II (Quanta BioSciences, Gaithersburg, Maryland, USA). Amplification occurred under the conditions: 94 °C for 3 min, followed by 35 cycles of 94 °C for 45 s, 50 °C for 60 s and 72 °C for 90 s, followed by the final elongation step at 72 °C for 10 min. After PCR amplification, samples were purified by the Agencourt Ampure XP protocol (Roche Molecular Systems, California, USA) following the manufacturer's protocol. The multiplexed libraries were submitted to the Center for Genome Research and Biotechnology (CGRB) at Oregon State University, Corvallis - Oregon USA - for sequencing on the Illumina MiSeq platform.

**Bioinformatics Analysis**

The raw data were analyzed using Mothur version.1.39.5. After checking for barcodes and primers in the forward and reverse sequences, data were paired and merged into contigs after verification. Contigs of less than 290 base pairs (bp) and greater than 352 bp, with no ambiguity and containing more than 8 homopolymers, were removed. The sequences were aligned using the SILVA database as a reference. *Screen-seqs* (Mothur version.1.39.5) were performed to remove poorly aligned sequences and *filter-seqs* to remove columns without sequence information. These sequences were pre-clustered using the *pre.cluster* command to reduce noise allowing up to two differences between the sequences with the parameter difs = 2. To improve the quality of the sequences the chimeras were detected and eliminated through the *chimera.vsearch* and *remove.seqs* commands. Sequences were sorted with the *classify.seqs* command using the Ribosomal Database Project (RDP) 16S rRNA version 16 as a reference and with a bootstrap value of 80. All chloroplast, mitochondria, Eukarya, and Archaea sequence identifications were removed, and the sequences were grouped in Operative Taxonomic Units (OTUs) with dissimilarity of 3 %. All the samples were normalized to the same number of sequences (5371).
